# Supplementary material for: Cohort profile: Oxford Pain, Activity and Lifestyle (OPAL) Study, a prospective cohort study of older adults in England
Source: BMJ Open. 2020 Sep 3;10(9):e037516. doi: 10.1136/bmjopen-2020-037516 (PMC7473632; doi:10.1136/bmjopen-2020-037516)
Supplement: Supplementary data [file bmjopen-2020-037516supp001.pdf]

Supplementary Data

Supplemental Figure S1. Flow chart of baseline participants in the OPAL cohort study

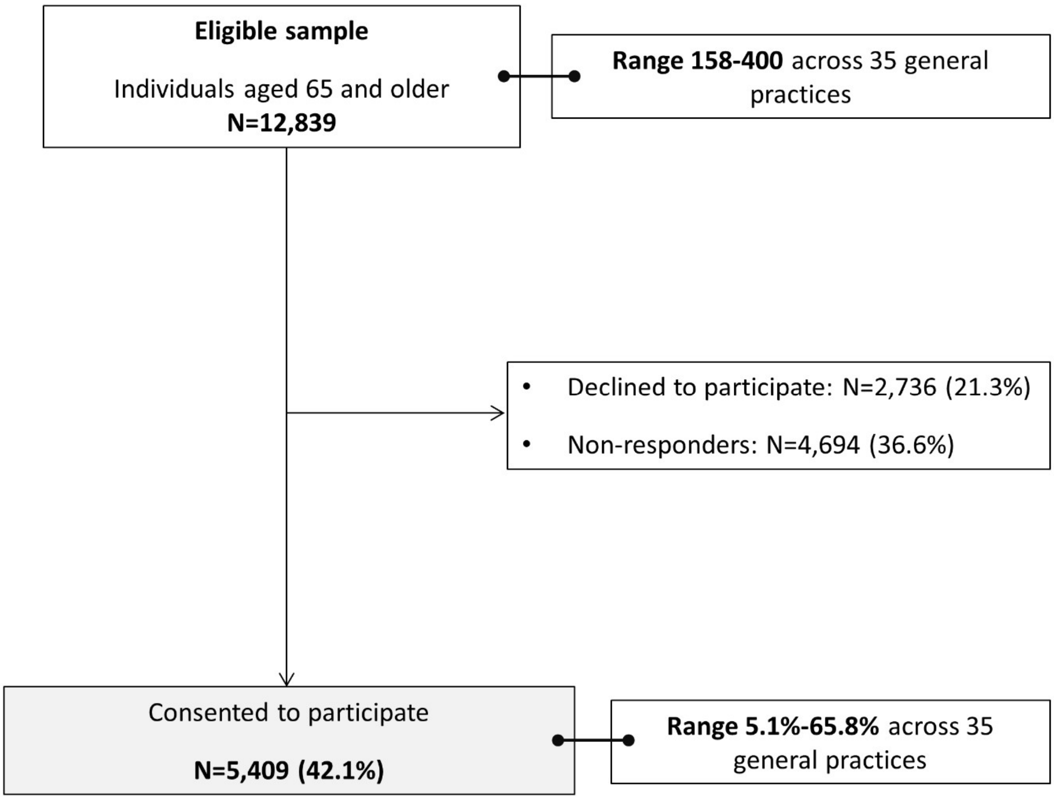

Sanchez Santos MT, *et al. BMJ Open* 2020; 10:e037516. doi: 10.1136/bmjopen-2020-037516

Supplemental Table S1. Variables used in the OPAL and the ELSA cohort studies.

Variable	Question(s), answer(s) posed to OPAL study	Question(s), answer(s) posed to ELSA study	Name (label) of the variable used for the comparison study
age	Date of birth and date of completion of questionnaire	Age in 5 year bands<br>• 65-69<br>• 70-74<br>• 75-79<br>• 80-84<br>• 85+	'ageg5' (Age variable in 5 year bands) (Derived variable from Institute for fiscal studies (IFS))
Sex	Gender: Male and Female	Sex: Male and Female	'indsex' (Sex variable)
Work status	Which of the following best describes your CURRENT work status?<br>• Retired	Which of the following best describes your CURRENT work status?<br>• Retired	'wpdes' (Best description of current situation)
Relationship status	What is your current relationship status?<br>• Married/Civil Union	What is your current legal marital status?<br>• Married/Civil partner	'dimarr' (Marital status - combined marriage/civil partnership)
Weight	What is your weight?<br>• In Kilograms	Weight measurement<br>• In Kilograms<br>Note: Participants with weight of 37 kg or lower were excluded from the analysis of this variable (n=282) due to the lowest cut-off used in the OPAL cohort study	'estwt' (Final measured or estimated weight (kg))
Smoking status	Which of the following describes your current cigarette smoking status?<br>• Never<br>• Ex-smoker<br>• Current smoker	Smoker status (past or present):<br>• Never<br>• Ex-smoker<br>• Current smoker	'smokerstat' (Derived variable from IFS (non-financial))
Chronic health conditions	*Has your doctor or nurse ever told you that you have any of the following conditions?*	*Our records show that in the last interview you said that you had been told by a doctor that you had any of the following conditions.*<br><br>*Do you still have the condition?*	*Diagnosed last interview AND confirms previous chronic condition*<br><br>*OR*<br><br>*Chronic condition since last interview*

		Since last interview, has a doctor ever told you that you have any of the conditions on this card?	
Heart problems	• Angina or heart troubles	• Angina<br>• A heart attack (including myocardial infarction or coronary thrombosis)<br>• Congestive heart failure<br>• A heart murmur<br>• An abnormal heart rhythm<br>• Any other heart trouble	*Angina*: 'hedawan', 'hedacan', 'hediman'<br>*Heart attach*: 'hedawmi', 'hedacmi', 'hedimmi'<br>Congestion heart failure: 'hedawhf', 'hedachf', 'hedimhf'<br>*Heart murmur*: 'hedawhm', 'hedachm', 'hedimhm'<br>*Abnormal heart rhythm*: 'hedawar', 'hedacar', 'hedimar'<br>*Other*: 'hedaw95', 'hedac95', 'hedia95'
Diabetes	• Diabetes (Types I or II)	• Diabetes or high blood sugar	'hedawdi', 'hedacdi', 'hedimdi'
High blood pressure	• High blood pressure	• High blood pressure or hypertension	'hedawbp', 'hedacbp', 'hedimbp'
Stroke	• Stroke	• A stroke (cerebral vascular disease)	'hedawst', 'hedacst', 'hedimst'
Arthritis	• Arthritis	• Arthritis (including osteoarthritis, or rheumatism)	'hedbwar', 'hedbdar', 'hedibar'
Dementia	• Dementia	• Dementia, senility, or any other serious memory impairment	'hedbwde', 'hedbdde', 'hedibde'
Osteoporosis	• Osteoporosis	• Osteoporosis, sometimes called thin or brittle bones	'hedbwos', 'hedbdos', 'hedibos'
Chronic lung disease	• Chronic lung disease or Asthma	• Chronic lung disease such as chronic bronchitis or emphysema<br>• Asthma	*Chronic lung disease*: 'hedbwlu', 'hedbdlu', 'hediblu'<br><br>*Asthma*: 'hedbwas', 'hedbdas', 'hedibas'

**Variable**	**Question, answer(s) posed to OPAL study**	**Question, answer(s) posed to England 2011 Census**	**Variable used for the comparison study**
Ethnicity	To which of these ethnic groups do you consider you belong?<br>• Non-white (Mixed, Indian, Pakistani, Bangladeshi, Black/Black British, Chinese and other ethnic group)	What is your ethnic group?<br>• Non-white (Mixed/multiple ethnic groups, Asian/Asian British, Black/African/Caribbean/Black British and other ethnic group)	Ethnicity - divided into white and non-white

Sanchez Santos MT, *et al. BMJ Open* 2020; 10:e037516. doi: 10.1136/bmjopen-2020-037516

Supplemental Table S2. Characteristics of OPAL participants and non-participants

Characteristic	Eligible (N=12,839)	Responders		Non-Responders (N=4,694)	Non-participants (N=7,430)
		Consented (N=5,409)	Declined (N=2,736)		
Age*, mean (SD)	75.5 (7.2)	74.9 (6.8)	77.0 (7.4)	75.4 (7.4)	75.9 (7.4)
Age* groups, n (%)					
65-69	3,611 (28.1)	1,601 (29.6)	598 (21.9)	1,412 (30.1)	2,010 (27.1)
70-74	3,124 (24.3)	1,426 (26.4)	598 (21.9)	1,100 (23.4)	1,698 (22.9)
75-79	2,690 (21.0)	1,155 (21.4)	615 (22.5)	920 (19.6)	1,535 (20.7)
80+	3,414 (26.6)	1,227 (22.7)	925 (33.8)	1,262 (26.9)	2,187 (29.4)
Sex, n (%)*					
Male	5,943 (47.7)	2,625 (48.5)	1,159 (43.7)	2,226 (49.0)	3,385 (47.1)
Female	6,506 (52.3)	2,784 (51.5)	1,492 (56.3)	2,313 (51.0)	3,805 (52.9)

We did not have sex available for one site, so it was excluded for the analysis (N=390).
*The age of eligible individuals was calculated based on date when the questionnaire was sent and date of birth.

Supplemental Figure S2. Age distribution between participants (dark blue) and non-participants (pink) in the OPAL cohort study by general practice.

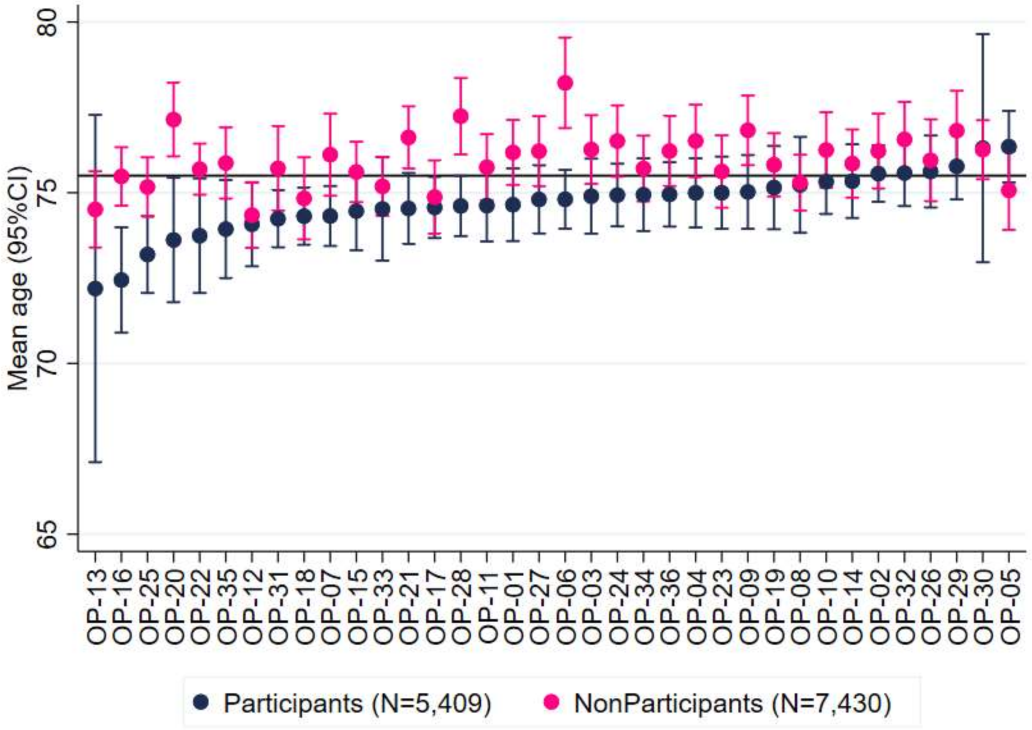

Sanchez Santos MT, *et al. BMJ Open* 2020; 10:e037516. doi: 10.1136/bmjopen-2020-037516

Supplemental Figure S3. Sex distribution between participants (dark blue) and non-participants (pink) in the OPAL cohort study by general practice.

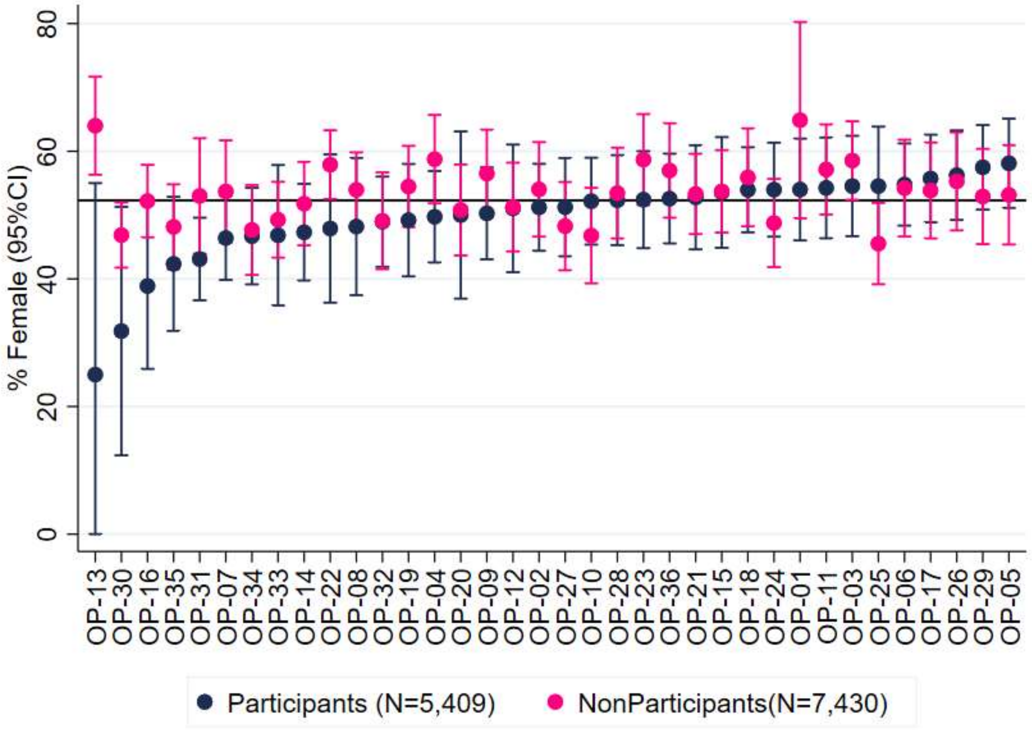

Supplemental Table S3. Area deprivation and ethnicity based on each general practice.

General practice	Eligible individuals	%Response rate	Practice IMD 2015 decile (1 More deprived to 10 Least deprived)	Estimated proportion of non-white ethnic groups in practice population		
				%Mixed	%Asian	%Black
OP-01	390	38.5%	-	-	-	-
OP-02	381	54.3%	10	2.3	5.8	1.8
OP-03	400	38.5%	4	4.4	12.8	4.1
OP-04	381	49.1%	10	2.1	5.0	1.7
OP-05	349	54.7%	10	3.4	9.1	1.8
OP-06	396	58.1%	10	1.7	2.1	1.3
OP-07	371	59.8%	10	1.4	3.6	0.0
OP-08	361	23.0%	1	6.9	36.8	21.3
OP-09	385	48.1%	5	4.6	15.7	4.0
OP-10	378	54.8%	10	1.1	1.1	0.0
OP-11	342	44.7%	6	4.3	12.7	6.8
OP-12	295	32.5%	2	4.7	6.1	5.0
OP-13	158	5.1%	1	3.9	62.0	19.1
OP-14	391	42.7%	6	4.4	13.9	6.5
OP-15	356	35.7%	2	2.5	5.9	2.7
OP-16	351	15.4%	1	6.1	31.4	16.7
OP-17	370	54.3%	7	2.2	5.3	2.0
OP-18	376	57.2%	10	1.4	3.7	0.0
OP-19	359	34.5%	5	4.4	10.9	6.3
OP-20	245	22.9%	6	2.2	7.4	3.4
OP-21	386	37.3%	3	0.0	1.1	0.0
OP-22	394	18.0%	1	1.5	2.5	1.7
OP-23/36*	350/366	47.4%/53.0%	8	2.0	3.2	0.0
OP-24	377	46.7%	7	0.0	1.2	0.0
OP-25	345	31.9%	3	3.9	15.2	4.4
OP-26	353	54.4%	8	0.0	0.0	0.0
OP-27	363	44.6%	-	-	-	-
OP-28	382	50.5%	10	0.0	1.2	0.0
OP-29	396	55.4%	9	1.0	1.8	0.0
OP-30	389	5.7%	-	-	-	-
OP-31	342	65.8%	8	0.0	1.1	0.0
OP-32	359	53.5%	9	0.0	0.0	0.0
OP-33	351	22.5%	4	7.2	26.9	33.8
OP-34	360	46.4%	8	1.4	2.8	0.0
OP-35	301	28.2%	3	7.2	29.6	28.2

IMD=Index of Multiple deprivation. 8 general practices had a response rate below (red) and 13 above (green) to the expected rate (<30% and >50%, respectively). Information found on the following government website: https://fingertips.phe.org.uk/profile/general-practice (Accessed August 2019). *Two different random samples of individuals were selected from the same general practice.

Supplemental Table S4. Age and ethnicity distribution in the OPAL and population estimates 2011 Census in England by sex

	**OPAL (Observed %)**			**2011 England Census (%)**		
	**Overall**	**Female**	**Male**	**Overall**	**Female**	**Male**
Age						
65-69	29.3	28.8	29.9	29.0	26.8	31.7
70-74	26.4	26.4	26.5	23.6	22.3	25.2
75-79	21.5	22.2	20.7	19.3	19.0	19.7
80 and over	22.8	22.7	23.0	28.2	31.9	23.5
Ethnicity, non-white	5.1	4.1	6.0	4.7	4.5	5.1
N	5,409	2,784 (51.5)	2,625 (48.5)	8,660,529	4,815,690 (55.6)	3,844,839 (44.4)

The 2011 England Census data were collected from: https://www.ons.gov.uk/

Supplemental Table S5. Characteristics of **women** in the OPAL and ELSA cohort studies by age groups

**Characteristics**	**OPAL (Observed %)**				**ELSA (Estimated % [95%CI])**			
	**65-69**	**70-74**	**75-79**	**80+**	**65-69**	**70-74**	**75-79**	**80+**
Relationship status								
Married/Civil Union	66.0	62.5	52.4	30.7	69.0 [65.8-72.1]	64.6 [60.8-68.2]	54.9 [50.4-59.2]	30.1 [26.6-33.9]
Work status, Retired	75.9	87.3	92.7	91.9	77.3 [74.2-80.0]	88.5 [85.7-90.8]	90.2 [87.3-92.6]	93.2 [91.1-94.9]
Weight (kg), mean (SD)	70.7 (14.8)	69.7 (14.7)	68.4 (13.1)	65.2 (13.0)	73.9 [72.8-75.1]	72.8 [71.5-74.1]	70.3 [69.0-71.6]	66.2 [65.0-67.4]
Smoking status,								
Ex-Smoker	38.3	40.2	35.3	34.9	46.7 [43.3-50.2]	56.8 [52.9-60.7]	49.8 [45.3-54.2]	53.5 [49.5-57.4]
Current	5.2	4.8	5.0	1.6	11.6 [9.5-14.1]	9.1 [7.0-11.8]	7.1 [4.9-10.1]	3.9 [2.6-5.9]
Health conditions,								
Heart problems	8.6	12.4	15.7	27.7	18.5 [16.0-21.4]	21.2 [18.1-24.6]	25.7 [22.0-29.7]	34.3 [30.6-38.2]
Diabetes	8.7	11.3	11.5	14.3	11.4 [9.3-13.8]	14.1 [11.6-17.1]	13.4 [10.6-16.7]	16.9 [14.1-20.2]
High Blood pressure	32.5	42.4	48.7	54.4	36.5 [33.2-39.9]	41.3 [37.5-45.3]	50.7 [46.2-55.1]	57.6 [53.6-61.4]
Stroke	2.1	2.2	2.9	6.7	3.3 [2.3-4.8]	4.3 [2.9-6.2]	7.8 [5.7-10.6]	11.2 [9.0-13.9]
Arthritis	45.6	51.8	55.0	58.6	49.9 [46.4-53.3]	54.3 [50.4-58.2]	58.3 [53.8-62.6]	62.4 [58.4-66.1]
Dementia	0.1	0.3	0.7	1.7	0.3 [0.1-1.1]	1.5 [0.8-2.8]	1.5 [0.7-3.2]	5.7 [4.1-7.8]
Osteoporosis	9.7	11.0	15.2	19.2	13.6 [11.4-16.2]	18.1 [15.2-21.4]	16.8 [13.8-20.4]	21.2 [18.2-24.6]
Chronic lung disease	10.1	9.4	10.2	7.1	6.8 [5.3-8.8]	8.2 [6.3-10.7]	8.7 [6.4-11.7]	6.1 [4.6-8.2]
Unweighted N	801	734	618	631	888	679	534	720

ELSA=The English Longitudinal Study of Ageing, a national probability sample of non-institutionalised older people. Wave 8 (2016-2017) was used for this analysis. For variable definitions, see Supplemental Table S1 and for ELSA data management, see Stata do-file “Data_management_wave8_Dec2019.do”. Data were weighted to correct for non-response in the ELSA cohort study

Sanchez Santos MT, *et al. BMJ Open* 2020; 10:e037516. doi: 10.1136/bmjopen-2020-037516

Supplemental Table S6. Characteristics of **men** in the OPAL and ELSA cohort studies by age groups

**Characteristics**	**OPAL (Observed %)**				**ELSA (Estimated % [95%CI])**			
	**65-69**	**70-74**	**75-79**	**80+**	**65-69**	**70-74**	**75-79**	**80+**
Relationship status								
Married/Civil Union	75.8	75.9	72.1	63.7	76.5 [72.9-79.8]	78.0 [74.3-81.2]	72.6 [68.1-76.7]	64.2 [59.6-68.6]
Work status, Retired	71.1	81.8	90.6	94.5	74.1 [70.5-77.5]	88.0 [85.2-90.4]	93.9 [91.3-95.8]	97.3 [95.4-98.4]
Weight (kg), mean (SD)	85.0 (15.6)	83.7 (15.4)	81.5 (13.8)	78.1 (12.6)	87.3 [86.0-88.7]	84.5 [83.1-85.8]	81.6 [80.3-83.0]	78.6 [77.3-79.8]
Smoking status,								
Ex-Smoker	49.0	54.0	55.4	56.4	61.8 [57.8-65.6]	64.0 [59.9-67.9]	66.9 [62.2-71.3]	75.0 [70.8-78.7]
Current	8.8	5.2	4.6	2.5	9.7 [7.4-12.6]	10.2 [7.7-13.2]	8.2 [5.9-11.4]	2.3 [1.3-4.0]
Health conditions,								
Heart problems	18.4	25.0	27.7	32.2	20.2 [17.1-23.7]	28.5 [24.9-32.4]	35.1 [30.6-39.8]	40.2 [35.8-44.9]
Diabetes	16.1	15.4	16.1	17.1	14.5 [11.8-17.6]	18.0 [14.9-21.5]	19.4 [15.7-23.7]	15.6 [12.5-19.2]
High Blood pressure	44.3	48.7	48.0	49.4	39.5 [35.6-43.5]	47.5 [43.4-51.6]	49.8 [45.0-54.6]	51.9 [47.2-56.5]
Stroke	2.9	4.2	6.6	8.8	5.2 [3.6-7.4]	6.6 [4.8-9.1]	8.7 [6.3-11.7]	16.8 [13.5-20.6]
Arthritis	31.1	32.6	37.5	43.5	31.6 [27.9-35.4]	37.0 [33.1-41.1]	40.6 [36.0-45.4]	41.5 [37.0-46.2]
Dementia	0.4	0.4	0.7	2.0	0.5 [0.2-1.6]	1.7 [0.8-3.3]	2.3 [1.2-4.4]	4.9 [3.3-7.3]
Osteoporosis	1.4	2.3	3.5	3.7	2.0 [1.2-3.5]	5.7 [4.0-8.1]	3.6 [2.1-5.9]	3.5 [2.1-5.7]
Chronic lung disease	7.5	7.8	7.9	8.3	7.4 [5.4-10.0]	10.2 [7.9-13.1]	11.6 [8.8-15.2]	8.1 [5.9-10.9]
Unweighted N	784	696	542	603	659	624	458	503

ELSA=The English Longitudinal Study of Ageing, a national probability sample of non-institutionalised older people. Wave 8 (2016-2017) was used for this analysis. For variable definitions, see Supplemental Table S1 and for ELSA data management, see Stata do-file “Data_management_wave8_Dec2019.do”. Data were weighted to correct for non-response in the ELSA cohort study
