# Supplementary material for: Cohort profile: Oxford Pain, Activity and Lifestyle (OPAL) Study, a prospective cohort study of older adults in England
Source: BMJ Open. 2020 Sep 3;10(9):e037516. doi: 10.1136/bmjopen-2020-037516 (PMC7473632; doi:10.1136/bmjopen-2020-037516)
Supplement: Supplementary data [file bmjopen-2020-037516supp002.pdf]

Data_management_wave8_Dec2019 - Printed on 23/06/2020 15:19:32

```
1   ********************************************************************************
2   * University of Oxford
3   * ELSA
4   * December 2019
5   ********************************************************************************
6
7   *Data: wave_8_elsa_data_eul_v2.dta
8
9   *Variables needed for health comparison from Wave 8
10  *Identifier
11  * idauniq   Variable label = Unique individual serial number
12  * idahhw8   Variable label = Analytical wave-specific individual
13  * perid     Variable label = Person ID
14  * samptyp   Variable label = Sampling status
15  * w8xwgt    Variable label = Wave 8 cross-sectional weight
16  * w8indout  Variable label = Individual outcome code
17
18  *Demography
19  * Derived variables are denoted with "(D)" at the beginning of the variable label:
20  * indager   Variable label = (D) Definitive age variable collapsed at 90+: priority diag,
    dhage
21  * indsex    Variable label = (D) Definitive sex variable: priority disex, dhsex
22  * fqethnmr  Variable label = (D) Ethnicity recoded into white and non-white (consolidated)
23  * wpdes     Variable label = Best description of current situation (retired)
24  * dimarr    Variable label = (D) Respondent current legal marital status - combined
    marriage/civil partnership
25  * estwt     Variable label = (D) Weight: final measured or estimated weight (kg)
26
27  *Health-related variables
28  *--Heart problems
29  *Angina
30  * hedawan   Variable label = Diagnosed angina fed forward
31  * hedacan   Variable label = Whether confirms angina diagnosis
32  * hediman   Variable label = Cardiovascular disease: angina diagnosis newly reported
    (merged)
33  *Heart attack
34  * hedawmi   Variable label =  Diagnosed heart attack fed forward
35  * hedacmi   Variable label =  Whether confirms heart attack diagnosis
36  * hedimmi   Variable label = Cardiovascular disease: heart attack diagnosis newly
    reported (merged)
37  *Congestive heart failure
38  * hedawhf   Variable label =  Diagnosed congestive heart failure fed forward
39  * hedachf   Variable label =  Whether confirms congestive heart failure diagnosis
40  * hedimhf   Variable label =  Cardiovascular disease: congestive heart failure diagnosis
    newly reported (merged)
41  *Heart murmur
42  * hedawhm   Variable label =  Diagnosed heart murmur fed forward
43  * hedachm   Variable label =  Whether confirms heart murmur diagnosis
44  * hedimhm   Variable label =  Cardiovascular disease: heart murmur diagnosis newly
    reported (merged)
45  *Abnormal heart rhythm
46  * hedawar   Variable label =  Diagnosed abnormal heart rhythm fed forward
47  * hedacar   Variable label =  Whether confirms abnormal heart rhythm diagnosiss
48  * hedimar   Variable label =  Cardiovascular disease: abnormal heart rhythm diagnosis
    newly reported (merged)
49  *Other heart disease
50  * hedaw95   Variable label =  Diagnosed other heart disease fed forward
51  * hedac95   Variable label =  Whether confirms other heart disease diagnosis
52
53  *--Diabetes
54  *Diabetes or high blood sugar
55  * hedawdi   Variable label = Diagnosed diabetes or high blood sugar fed forward
56  * hedacdi   Variable label = Whether confirms diabetes or high blood sugar diagnosis
57  * hedimdi   Variable label = Cardiovascular disease: diabetes or high blood sugar
    diagnosis newly reported (merged)
58  *--High blood pressure
59  *High blood pressure
60  * hedawbp   Variable label = Diagnosed high blood pressure fed forward
61  * hedacbp   Variable label = Whether confirms high blood pressure diagnosis
62  * hedimbp   Variable label = Cardiovascular disease: high blood pressure diagnosis newly
    reported (merged)
63  *--Stroke
64  *Stroke
65  * hedawst   Variable label = Diagnosed stroke fed forward
66  * hedacst   Variable label = Whether confirms stroke diagnosis
```

Data_management_wave8_Dec2019 - Printed on 23/06/2020 15:19:32

```
67   * hedimst   Variable label = Cardiovascular disease: stroke diagnosis newly reported
     (merged)
68   *--Arthritis
69   *Arthritis
70   * hedbwar   Variable label = Chronic: diagnosed arthritis fed forwar
71   * hedbdar   Variable label = Whether confirms arthritis diagnosis
72   * hedibar   Variable label = Chronic: arthritis diagnosis newly reported
73   *--Osteoporosis
74   *Osteoporosis
75   * hedbwos   Variable label = Chronic: diagnosed osteoporosis fed forward
76   * hedbdos   Variable label = Whether confirms osteoporosis diagnosis
77   * hedibos   Variable label = Chronic: osteoporosis diagnosis newly reported
78   *--Dementia
79   *Dementia
80   * hedbwde   Variable label = Chronic: diagnosed dementia fed forward
81   * hedbdde   Variable label = Whether confirms dementia diagnosis
82   * hedibde   Variable label = Chronic: dementia diagnosis newly reported
83   *--Chronic lung disease
84   *Chronic lung disease
85   * hedbwlu   Variable label = Chronic: diagnosed lung disease fed forward
86   * hedbdlu   Variable label = Whether confirms lung disease diagnosis
87   * hediblu   Variable label = Chronic: lung disease diagnosis newly reported
88   *Asthma
89   * hedbwas   Variable label = Chronic: diagnosed asthma fed forward
90   * hedbdas   Variable label = Whether confirms asthma diagnosis
91   * hedibas   Variable label = Chronic: asthma diagnosis newly reported
92
93   ************************************************************************
94
95   *Data: wave_8_elsa_data_eul_v2.dta ((IFS derived databaset))
96
97   *Variables needed for health comparison from Wave 8
98   *Identifier
99   * idauniq   Variable label = Unique individual serial number
100  * idahhw8   Variable label = Analytical wave-specific individual
101  * w8xwgt    Variable label = Wave 8 cross-sectional weight
102
103  *Demography
104  * ageg5     Variable label = age band - 5 year bands  (8 way split)
105  * sex       Variable label = sex: copy of indsex/dhsex
106  * elsa      Variable label = Sampling status
107  * inst      Variable label = whether in an institution
108  * nonwhite  Variable label = ethic origin (white/non-white)
109  * marstat   Variable label = marital status - couple1 combined with dimar
110  * smoker    Variable label = whether current smoker
111  * smokerstat Variable label = smoker status (past or present)
112
113
114  ************************************************************************
115  version 15.1
116  clear all
117
118  cd "\Data" /* Change working directory */
119
120  *---REVIEWERS COMMENTS.
121
122  use "wave_8_elsa_data_eul_v2.dta", clear
123  tab indsex
124  /* 8445 */
125
126  keep idauniq idahhw8 perid samptyp w8xwgt indager indsex fqethnmr wpdes dimarr estwt
     hedawan hedacan hediman hedawmi hedacmi hedimmi ///
127           hedawhf hedachf hedimhf hedawhm hedachm hedimhm hedawar hedacar hedimar hedaw95
     hedac95 hedia95 hedawdi hedacdi hedimdi ///
128           hedawbp hedacbp hedimbp hedawst hedacst hedimst hedbwar hedbdar hedibar hedbwos
     hedbdos hedibos ///
129           hedbwde hedbdde hedibde hedbwlu hedbdlu hediblu hedbwas hedbdas hedibas
130
131
132  merge 1:1 idauniq idahhw8 using "wave_8_elsa_ifs_dvs_eul_v1.dta", keepusing(wgt ageg5 sex
     elsa inst nonwhite smoker smokerstat)
133  tab _merge
134  drop _merge
135  save "ELSA Comparison.dta",replace
136
```

Data_management_wave8_Dec2019 - Printed on 23/06/2020 15:19:32

```
tab indsex
/* 8445 */

*------
*---EXCLUSION CRITERIA
*- 1. Noncore members (elsa=0 or samptyp=)
*- 2. Younger than 65 (age<65)
*- 3. Living in an institution (inst=1)

*N=8,445

*-CORE MEMBERS
tab1 elsa samptyp, m
tab samptyp elsa
*N=7,223

*-AGE
tab ageg5, m
*N=5,478


*-LIVING IN A INSTITUTION
tab inst, m
*N=58
*N=8,387

gen exclusion=1 if inst==1
replace exclusion=2 if ageg5<4
replace exclusion=3 if elsa==0
label var exclusion "Exclusion criteria"
label define exclusion 1 "Living in a institution" 2"Aged<65" 3"NoCore Members", replace
label values exclusion exclusion
tab exclusion, m


keep if exclusion==.
*N=5,065

***CLEANING AND TRANSFORMING VARIABLES

*ID
rename idauniq id
gen psu=id

*-AGE INTO 5 YEAR BANDS
tab ageg5, m

gen w8age4g=ageg5
recode w8age4g 4=1 5=2 6=3 7=4 8=4
label var w8age4g "Age into 4 groups"
label define w8age4g 1 "65-69" 2 "70-74" 3"75-79" 4"80 or more", replace
label values w8age4g w8age4g
tab w8age4g, m

tab w8age4g ageg5, m
drop ageg5

*-SEX
tab indsex

rename indsex w8sex

*1=male; 2=female
label var w8sex "Sex"

label define w8sex 1 "Male" 2 "Female", replace
label values w8sex w8sex
tab w8sex

*-ETHNICITY
tab fqethnmr nonwhite, m

rename fqethnmr w8white
recode w8white 1=1 2=0

```

Data_management_wave8_Dec2019 - Printed on 23/06/2020 15:19:32

```
*-MARITAL STATUS
tab dimarr, m
gen w8marstat=0
replace w8marstat=1 if  dimarr==2 | dimarr==3
replace w8marstat=. if  dimarr==-8
label var w8marstat "Current legal marital status - Married/civil partnership yes/no"
label define w8marstat 0"Other" 1"Married/civil", replace
label values w8marstat w8marstat
tab w8marstat

*-WORK STATUS
tab wpdes, m

gen w8retired=0
replace w8retired=1 if wpdes==1
label define w8retired 0"Other" 1"Retired", replace
label values w8retired w8retired
label var w8retired "Best description of current situation - Retired yes/no"
tab w8retired

*-WEIGHT
replace estwt=. if estwt<38 /***** CUT-OFF USED IN OPAL study *****/

rename estwt w8weightKg

*-SMOKE
tab smokerstat smoker
tab smokerstat, m

gen w8smokerstat=.
replace w8smokerstat=1 if smokerstat==0
replace w8smokerstat=2 if smokerstat==1 | smokerstat==2 | smokerstat==3
replace w8smokerstat=3 if smokerstat==4
lab define w8smokerstat 1"Never smoker" 2"Ex-smoker" 3"Current smoker", replace
label values w8smokerstat w8smokerstat
label variable w8smokerstat "Cigarette smoking status"

tab w8smokerstat, m

*--HEALTH-RELATED FACTORS

*Angina: 'hedawan', 'hedacan', 'hediman'
*Heart attach: 'hedawmi', 'hedacmi', 'hedimmi'
*Congestion heart failure: 'hedawhf', 'hedachf', 'hedimhf'
*Heart murmur: 'hedawhm', 'hedachm', 'hedimhm'
*Abnormal heart rhythm: 'hedawar', 'hedacar', 'hedimar'
*Other: 'hedaw95', 'hedac95', 'hedia95'

*-ANGINA
tab1 hedawan hedacan hediman
gen w8angina=0
replace w8angina=1 if (hedawan==2 & hedacan==1) | hediman==1
replace w8angina=. if hediman<0 & w8angina!=1

*-HEART ATTACK
tab1 hedawmi hedacmi hedimmi
gen w8heartattack=0 if hedawmi==-1
replace w8heartattack=1 if (hedawmi==3 & hedacmi==1) | hedimmi==1
replace w8heartattack=. if hedimmi<0 & w8heartattack!=1

*-CONGESTION HEART FAILURE
tab1 hedawhf hedachf hedimhf
gen w8heartfailure=0
replace w8heartfailure=1 if (hedawhf==4 & hedachf==1) | hedimhf==1
replace w8heartfailure=. if hedimhf<0 & w8heartfailure!=1

*-HEART MURMUR
tab1 hedawhm hedachm hedimhm
gen w8heartmurmur=0
replace w8heartmurmur=1 if (hedawhm==5 & hedachm==1) | hedimhm==1
replace w8heartmurmur=. if hedimhm<0 & w8heartmurmur!=1

*-ABNORMAL HEART RHYTHM
tab1 hedawar hedacar hedimar
gen w8heartrhythm=0
```

Data_management_wave8_Dec2019 - Printed on 23/06/2020 15:19:33

```
287   replace w8heartrhythm=1 if (hedawar==6 & hedacar==1) | hedimar==1
288   replace w8heartrhythm=. if hedimar<0 & w8heartrhythm!=1
289
290   *-OTHER
291   tab1 hedaw95 hedac95 hedia95
292   gen w8otherheartproblem=0
293   replace w8otherheartproblem=1 if (hedaw95==95 & hedac95==1) | hedia95==1
294   replace w8otherheartproblem=. if hedia95<0 & w8otherheartproblem!=1
295
296   *-HEART PROBLEMS
297   gen w8hearttroubles=0
298   replace w8hearttroubles=1 if w8angina==1 | w8heartattack==1 | w8heartfailure==1 |
      w8heartmurmur==1 | w8heartrhythm==1 | w8otherheartproblem==1
299   replace w8hearttroubles=. if w8angina==. & w8heartattack==. & w8heartfailure==. &
      w8heartmurmur==. & w8heartrhythm==. & w8otherheartproblem==.
300
301   *-DIABETES
302   tab1 hedawdi hedacdi hedimdi
303   gen w8diabetes=0
304   replace w8diabetes=1 if (hedawdi==7 & hedacdi==1) | hedimdi==1
305   replace w8diabetes=. if hedimdi<0 & w8diabetes!=1
306
307   *-HIGH BLOOD PRESSURE
308   tab1 hedawbp hedacbp hedimbp
309   gen w8hbp=0
310   replace w8hbp=1 if (hedawbp==1 & hedacbp==1) | hedimbp==1
311   replace w8hbp=. if hedimbp<0 & w8hbp!=1
312
313   *-STROKE
314   tab1 hedawst hedacst hedimst
315   gen w8stroke=0
316   replace w8stroke=1 if (hedawst==8 & hedacst==1) | hedimst==1
317   replace w8stroke=. if hedimst<0 & w8stroke!=1
318   tab w8stroke
319
320   *-ARTHRITIS
321   tab1 hedbwar hedbdar hedibar
322   gen w8arthritis=0
323   replace w8arthritis=1 if (hedbwar==3 & hedbdar==1) | hedibar==1
324   replace w8arthritis=. if hedibar<0 & w8arthritis!=1
325
326   *-DEMENTIA
327   tab1 hedbwde hedbdde hedibde
328   gen w8dementia=0
329   replace w8dementia=1 if (hedbwde==9 & hedbdde==1) | hedibde==1
330   replace w8dementia=. if hedibde<0 & w8dementia!=1
331
332   *-OSTEOPOROSIS
333   tab1 hedbwos hedbdos hedibos
334   gen w8osp=0
335   replace w8osp=1 if (hedbwos==4 & hedbdos==1) | hedibos==1
336   replace w8osp=. if hedibos<0 & w8osp!=1
337
338   *-CHRONIC LUNG DISEASE
339   tab1 hedbwlu hedbdlu hediblu
340   gen w8lungdisease=0
341   replace w8lungdisease=1 if (hedbwlu==1 & hedbdlu==1) | hediblu==1
342   replace w8lungdisease=. if hediblu<0 & w8lungdisease!=1
343
344   *-ASTHMA
345   tab1 hedbwas hedbdas hedibas
346
347   gen w8asthma=0
348   replace w8asthma=1 if (hedbwas==1 & hedbdas==1) | hedibas==1
349   replace w8asthma=. if hedibas<0 & w8asthma!=1
350
351   *-CHRONIC LUNG DISEASE + ASTHMA
352   gen w8cld=0
353   replace w8cld=1 if w8lungdisease==1 | w8asthma==1
354   replace w8cld=. if w8lungdisease==. & w8asthma==.
355
356   /*****   ANALYSIS  WITH WEIGHTED DATA ******/
357
358   svyset, clear
359   svyset [pweight=w8xwgt], psu(psu)
```

```
360
361
362   /***** FEMALE ****/
363
364   unab xvars: w8white w8marstat w8retired w8smokerstat w8hearttroubles w8diabetes w8hbp
      w8stroke w8arthritis w8dementia w8osp w8cld
365   foreach x of local xvars {
366   svy:tab `x' w8age4g if w8sex==2, col per ci
367   }
368
369   *---Weight (Kg)
370   forvalues i = 1/4 {
371   svy:mean w8weightKg if w8sex==2 & w8age4g==`i'
372   }
373
374   *---Unweighted N
375   tab w8age4g if w8sex==2
376
377
378   /***** MALE ****/
379   unab xvars: w8white w8marstat w8retired w8smokerstat w8hearttroubles w8diabetes w8hbp
      w8stroke w8arthritis w8dementia w8osp w8cld
380   foreach x of local xvars {
381   svy:tab `x' w8age4g if w8sex==1, col per ci
382   }
383
384   *---Weight (Kg)
385   forvalues i = 1/4 {
386   svy:mean w8weightKg if w8sex==1 & w8age4g==`i'
387   }
388
389   *---Unweighted N
390   tab w8age4g if w8sex==1
391
392
```
