# Supplementary material for: Cohort profile: Oxford Pain, Activity and Lifestyle (OPAL) Study, a prospective cohort study of older adults in England
Source: BMJ Open. 2020 Sep 3;10(9):e037516. doi: 10.1136/bmjopen-2020-037516 (PMC7473632; doi:10.1136/bmjopen-2020-037516)
Supplement: Author's manuscript [file bmjopen-2020-037516.draft_revisions.pdf]

BMJ Open is committed to open peer review. As part of this commitment we make the peer review history of every article we publish publicly available.

When an article is published we post the peer reviewers' comments and the authors' responses online. We also post the versions of the paper that were used during peer review. These are the versions that the peer review comments apply to.

The versions of the paper that follow are the versions that were submitted during the peer review process. They are not the versions of record or the final published versions. They should not be cited or distributed as the published version of this manuscript.

BMJ Open is an open access journal and the full, final, typeset and author-corrected version of record of the manuscript is available on our site with no access controls, subscription charges or pay-per-view fees (http://bmjopen.bmj.com).

If you have any questions on BMJ Open's open peer review process please email info.bmjopen@bmj.com

BMJ Open

Cohort profile: Oxford Pain, Activity and Lifestyle (OPAL) study, a prospective cohort study of older adults in England

Journal:	*BMJ Open*
Manuscript ID	bmjopen-2020-037516
Article Type:	Cohort profile
Date Submitted by the Author:	05-Feb-2020
Complete List of Authors:	Sanchez Santos, Maria ; University of Oxford, Centre for Rehabilitation Research in Oxford, Nuffield Department of Orthopaedics, Rheumatology and Musculoskeletal Sciences<br>Williamson, Esther; University of Oxford, Centre for Rehabilitation Research in Oxford, Nuffield Department of Orthopaedics, Rheumatology and Musculoskeletal Sciences<br>Bruce, Julie; University of Warwick, Warwick Clinical Trials Unit<br>Ward, Lesley; University of Oxford, Centre for Rehabilitation Research in Oxford, Nuffield Department of Rheumatology, Orthopaedics and Musculoskeletal Sciences; Northumbria University, Department of Sport, Exercise & Rehabilitation<br>Mallen, Christian; Keele University, Arthritis Research UK Primary Care Centre<br>Garrett, Angela; University of Oxford, Centre for Rehabilitation Research in Oxford, Nuffield Department of Orthopaedics Rheumatology and Musculoskeletal Sciences<br>Morris , Alana ; University of Oxford, Centre for Rehabilitation Research in Oxford, Nuffield Department of Orthopaedics Rheumatology and Musculoskeletal Sciences<br>Lamb, Sarah; University of Exeter, College of Medicine and Health; University of Oxford, Centre for Rehabilitation Research in Oxford, Nuffield Department of Orthopaedics Rheumatology and Musculoskeletal Sciences
Keywords:	EPIDEMIOLOGY, GERIATRIC MEDICINE, Musculoskeletal disorders < ORTHOPAEDIC & TRAUMA SURGERY, PRIMARY CARE
Note: The following files were submitted by the author for peer review, but cannot be converted to PDF. You must view these files (e.g. movies) online.	
Data_management_wave8_Dec2019.do	

SCHOLARONE™ Manuscripts

I, the Submitting Author has the right to grant and does grant on behalf of all authors of the Work (as defined in the below author licence), an exclusive licence and/or a non-exclusive licence for contributions from authors who are: i) UK Crown employees; ii) where BMJ has agreed a CC-BY licence shall apply, and/or iii) in accordance with the terms applicable for US Federal Government officers or employees acting as part of their official duties; on a worldwide, perpetual, irrevocable, royalty-free basis to BMJ Publishing Group Ltd ("BMJ") its licensees and where the relevant Journal is co-owned by BMJ to the co-owners of the Journal, to publish the Work in this journal and any other BMJ products and to exploit all rights, as set out in our licence.

The Submitting Author accepts and understands that any supply made under these terms is made by BMJ to the Submitting Author unless you are acting as an employee on behalf of your employer or a postgraduate student of an affiliated institution which is paying any applicable article publishing charge ("APC") for Open Access articles. Where the Submitting Author wishes to make the Work available on an Open Access basis (and intends to pay the relevant APC), the terms of reuse of such Open Access shall be governed by a Creative Commons licence – details of these licences and which Creative Commons licence will apply to this Work are set out in our licence referred to above.

Other than as permitted in any relevant BMJ Author's Self Archiving Policies, I confirm this Work has not been accepted for publication elsewhere, is not being considered for publication elsewhere and does not duplicate material already published. I confirm all authors consent to publication of this Work and authorise the granting of this licence.

For peer review only

Cohort profile: Oxford Pain, Activity and Lifestyle (OPAL) study, a prospective cohort study of older adults in England

Maria T Sanchez-Santos[1], Esther Williamson[1], Julie Bruce[2], Lesley Ward[1,3], Christian D. Mallen[4], Angela Garrett[1], Alana Morris[1], Sarah E Lamb[1,5] on behalf of the OPAL study team

Authors' affiliations:

[1]Centre for Rehabilitation Research, Nuffield Department of Rheumatology, Orthopaedics and Musculoskeletal Sciences, University of Oxford, Oxford, United Kingdom (UK)

[2]Warwick Clinical Trials Unit, Division of Health Sciences, University of Warwick, Coventry, UK

[3]Department of Sport, Exercise & Rehabilitation, Northumbria University, Newcastle-upon-Tyne

[4]School of Primary, Community and Social Care, Keele University, Staffordshire

[5]College of Medicine and Health, University of Exeter, UK

Corresponding Author: Sarah E Lamb

Email: S.E.Lamb@exeter.ac.uk

Tel: +44(0)1392 722 370

Heavitree Road, Exeter, Devon, EX1 2LU

Words: Abstract: 289; Main text: 3,806; **Tables:** 3; **Figures:** 2

Supplementary files: 2; **References**: 57

For peer review only

Abstract

PURPOSE: The 'Oxford Pain, Activity and Lifestyle' (OPAL) cohort is a longitudinal, prospective cohort study of adults, aged 65 years and older, living in the community which is investigating the determinants of health in later life. The initial focus was on musculoskeletal pain and mobility, but the cohort was designed with flexibility to include new elements over time. This paper describes the study design, data collection, and baseline characteristics of participants. We also compared the OPAL baseline characteristics with a representative sample of community-dwelling older people, The English Longitudinal Study of Ageing (ELSA).

PARTICIPANTS: We randomly selected eligible participants from two stratified age bands (65-74 and 75 and over years). In total, 5,409 individuals (42.1% of eligible participants) from 35 general practices agreed to participate between 2016 and 2018. The majority of participants (n=5,367) also consented for research team to access their UK NHS Digital and primary health care records.

FINDINGS TO DATE: Mean participant age was 74.9 years (range 65-100); 51.5% (n=2,784/5,409) were women. 94.9% of participants were white, and 28.8% lived alone. Over 83.0% reported pain in at least one body area in the previous six weeks. Pain was more prevalent in women (86.0%). Over 29.0% of participants reported having one or more falls in the last year. Most participants were confident in their ability to walk outside. Characteristics of OPAL participants were similar to the ELSA population.

FUTURE PLANS: Postal follow-up of the cohort is being undertaken at annual intervals, with data collection ongoing. Linkage to NHS hospital admission data is planned. This English prospective cohort offers a large and rich resource for research on the longitudinal associations between demographic, clinical, and social factors and health trajectories and outcomes in older people living in the community.

For peer review only - http://bmjopen.bmj.com/site/about/guidelines.xhtml

Strengths and limitations of this study

- OPAL is a new, high quality cohort of older community-dwelling men and women exploring causes and consequences of pain, frailty, mobility decline, disability and poor health-related quality of life.
- A total of 5,409 older adults from 35 general practices in nine distinct areas in England participated at baseline, 2016-2018.
- The data comprise a wide range of self-reported variables. These include lifestyle measures, attitudes and beliefs, socioeconomic status and health-related outcomes.
- Nearly all participants (n=5,367/5,409; 99.2%) have given informed consent to access their UK NHS Digital and primary health care data.
- OPAL participants are similar to those in general population.

Introduction

The population of the United Kingdom (UK) is undergoing a fundamental change in its age structure, due to lower birth rates and extended life expectancy. One in four people in the UK are projected to be aged 65 or over by 2050, with 15% aged over 75 years and 5% aged 85 years or older[1]. This change reflects gains in health and social development, and it is important that as many years of life are spent in good health as possible.

Active independence is one of the key concerns of older people, and mobility is critically important to this[2 3]. Older people value their mobility highly and they consider mobility loss as a key disadvantage of aging[4]. Poor or limited mobility is linked to functional decline, mortality, and increased health care utilization[5]. Conceptually, factors associated with mobility decline precede

For peer review only - http://bmjopen.bmj.com/site/about/guidelines.xhtml

disability within models of disablement. Therefore, identification of factors associated with mobility decline are important for prevention of, and rehabilitation from, mobility decline[6].

Musculoskeletal pain is one of the leading causes of disability and disease burden worldwide among community-dwelling older adults[7 8]. A recent review estimated that the prevalence of chronic pain among older adults in the UK ranged from 42% in 65-74 years old to 62% in the over 75 age group[9]. These estimates are similar to other developed countries[10].

Musculoskeletal pain has a large impact on many other aspects of older people's health such as loss of mobility, frailty, cognitive impairment, falls, and poor sleep quality[11-15]. However, the role of musculoskeletal pain on adverse health outcomes in older adults is poorly understood.

In order to address these knowledge gaps, we assembled the Oxford Pain, Activity and Lifestyle (OPAL) cohort, a prospective study of community dwelling older adults. The immediate objectives were:

- To investigate the causes and consequences of mobility decline and disability in later life, and the role and contribution of musculoskeletal pain and other factors;
- To develop a prognostic tool to assess mobility decline in a population-based cohort of older adults in UK;
- To investigate factors that moderate or mediate the effects of musculoskeletal pain on health outcomes.

In addition, we intend to use the OPAL cohort to identify potential participants for future clinical trials in disability prevention in later life and to study disablement and multi-morbidity more broadly. The 'cohort multiple randomised controlled trials' design is becoming increasingly common[16 17]. The concept is to use data collected in a cohort to identify people with specific health conditions and

For peer review only - http://bmjopen.bmj.com/site/about/guidelines.xhtml

then, as and when the opportunity arises, invite them to participate in a clinical trial relevant to their condition.

In this paper, we describe the OPAL cohort, design, data collection, and the profile of the participants at baseline and their overall representativeness of the English general population.

Cohort description

Study design

A population-based, longitudinal, prospective cohort study in England, using a combination of annually administered, self-reported questionnaires and routinely collected health data.

Practice and participant identification

General practice identification

General practices who were predominantly working with the UK NIHR Clinical Research Network (CRN) were approached to take part in the study. In terms of geographical spread, we included a range of rural and urban areas across England, to capture diversity in both socioeconomic and ethnic profiles.

Participant identification

Eligible Participants were identified from electronic record searches of general practice lists. A random sample of approximately 400 individuals (median: 365; range 158-400) per practice was selected (Figure 1) and stratified to ensure equal representation in the following two age bands: 65-74 years and 75 years and over (~200 individuals per practice within each age group). We estimated response rate between 30-40% amongst eligible participants based on previous experience of recruitment of older people from general practice[18].

For peer review only - http://bmjopen.bmj.com/site/about/guidelines.xhtml

Inclusion criteria

People registered with a general practice, aged 65 years and older, and living in the community, including sheltered or supported housing, were eligible for invitation.

Exclusion criteria

Individuals were excluded if they lived in a residential care or nursing home. Following the generation of the random sample, a designated General Practitioner (GP) or research nurse per practice screened the list to exclude those with known terminal illness with a life expectancy of less than six months, who presented with severe health or social concerns sufficient to preclude approach, or who were considered unable to provide informed consent.

Recruitment and enrolment

Recruitment and enrolment to OPAL commenced in October 2016 and it was completed in September 2018. A total of 12,839 individuals from thirty-five general practices in nine different areas of England were invited to take part in the study (Figure 1). A pack including an invitation letter, participant information leaflet, consent form, baseline questionnaire, and a postage paid return envelope was sent by the general practice. Five thousand four hundred and nine (42.1% of those eligible; range 5.1%-65.8% across practices) individuals who returned the baseline questionnaire and a signed consent form to the University of Oxford study office were enrolled in the study (Figure 1). One-fifth (21.3% of those eligible; n=2,736/12,839) declined participation and 4,694 (36.6%; n=4,694/12,839) did not respond. Non-responders were sent one postal reminder, four weeks after the original invitation. If no response was received, no further contact was made.

For peer review only - http://bmjopen.bmj.com/site/about/guidelines.xhtml

How often are they being followed up?

Study participants are being followed by postal questionnaire at annual intervals. First year follow up has now been completed, and second and third year follow-up will be concluded around September 2020 and 2021, respectively. Future follow-up questionnaires will be sent at four and five years from the date of the original invitation.

What is being measured?

Postal self-completed questionnaire

The OPAL cohort study includes information on a range of domains including demographic, socioeconomic, lifestyle variables, social participation, attitudes to ageing, musculoskeletal pain, health-related factors, comorbidity, mobility, disability, frailty, cognitive function, health-related quality of life, and medications (see Table 1).

Musculoskeletal pain is assessed by asking the participant if they have experienced pain in nine different body sites (knees, hands/wrists, neck, shoulders, hips, feet/ankles, elbows, lower and upper back) during the last six weeks[19 20]. Information on presence, frequency, troublesomeness, onset, and description of back pain in the last six weeks was collected using recognised methods[20-22]. Information about the spread of back related symptoms was also included. To identify individuals with possible spinal stenosis we asked whether participant's pain travelled into their buttocks/legs, whether it was exacerbated while standing up or walking and whether the symptoms improved when sitting down or bending forward[23 24]. *Mobility* was assessed using different measures. Confidence to walk a half a mile was assessed using a single item from the Modified Gait Self-efficacy scale which is rated on a 1 'not confident at all' to 10 "totally confident" scale[25]. Participants also reported their perceived usual walking pace outdoors with six possible responses: "Unable to walk", "very slow", "stroll at an easy pace", "normal", "fairly brisk" and "fast". Change in mobility in the last year was

For peer review only - http://bmjopen.bmj.com/site/about/guidelines.xhtml

measured with the question "Compared with 1 year ago, how would you rate your walking in general?" (Response options: much better, somewhat better, about the same, somewhat worse or much worse than a year ago). Participant, family, friends or doctor's concerns about participant ability to walk and move around was measured using two questions. Potential responses were "Extremely", "A little concerned" or "Not concerned at all". Life-space mobility was measured using five questions from the life-space assessment (LSA) questionnaire [26]: ''During the past 4 weeks have you gone to: (1) other rooms in your home besides the room where you sleep? (2) An area outside of your home as your porch, deck or patio, hallway or garage? (3) Different places in your neighbourhood? (4) Locations outside of your neighbourhood, but within your city? and (5) places outside your town?'. *Falls* data were collected as recommended by the Prevention of Falls Network Europe, using a single question , "In the last 12 months, have you had any fall including a slip or trip following which you have come to rest on the ground, floor or lower level?"[27]. Three possible responses were available: not fallen, fallen once or more than once in the last year. *Frailty* was measured by The Tilburg Frailty Indicator (TFI)[28 29]. It is composed of two parts. The first part describes different determinants of frailty based on sociodemographic data and health related questions. The second part contains 15 items which measure three frailty domains: physical (8 items), psychological (4 items) and social (3 items). Frailty total scale and individual domain scores are derived from the second part. All items are rated as a binary response of either 0 or 1. Scores are the sum of the respective item points with a total score ranged from 0 to 15, with higher scores representing a higher level of frailty. A total score ≥5 points indicates that the individual is frail[28]. *Health-Related Quality of life (HRQoL)* was measured by the EuroQol-5D-5L (EQ-5D-5L) questionnaire, a generic measure of HRQoL that includes five levels of functioning from level 1 (no problems) to level 5 (severe or extreme problems)[30 31]. Additionally, respondents rated their current health status according to the EuroQol-Visual Analog Scale (EQ-VAS), from 0 (worst imaginable health) to 100 (best imaginable health). The

For peer review only - http://bmjopen.bmj.com/site/about/guidelines.xhtml

responses from the five domains were converted into a single EQ-5D index value using the EQ-5D-5L Crosswalk Index Value Calculator to produce a final QoL value[32 33]. The index values ranged between -0.594 (a state worse than death) to 1 (best possible health state).

New variables have been added to the follow up questionnaire (Table 1), allowing the cohort to be used for a wider range of analytical approaches and purposes, and to dovetail to recruitment of new clinical trials. The first follow up (Year 1) repeated baseline variables (Table 1) with the exception of ethnicity, number of children, height, education, lifetime physical activity, main occupation during lifetime, self-rating of strenuousness of occupation, and use of smart-phone or computer to access the internet. Variables were also added, including presence, frequency, troublesome, location and description of knee pain. The second wave of follow-up of data collection is collecting variables included in previous wave (Year 1) in addition to difficulty balancing whilst walking and difficulty in any of the following basic activities of daily living (ADL); bathing, transfers, toilet use, dressing and eating. Each activity is rated from 'no difficulty' to 'Unable to perform'.

Characteristics of participating general practices

General practice deprivation and estimated proportion of non-white ethnic groups in the practice population were obtained from Public Health England (PHE)[34]. Deprivation was measured by the Index of Multiple Deprivation 2015 (IMD2015)[35]. Practice IMD scores are practice population weighted based on the Lower Layer Super Output Areas (LSOAs) where the practice population resides.

For peer review only - http://bmjopen.bmj.com/site/about/guidelines.xhtml

Data management and quality control

All data are being processed and stored according to the Data Protection Act 2018. As the OPAL study started prior to the application of the General Data Protection Regulation (GDPR) 2018, all participants were sent an updated GDPR statement along with their next follow-up questionnaire.

A software application was developed to support the filtering and random sampling of individuals from the practice lists. Individual identifiable data were removed by the application. When eligible participants were selected, a unique screening number was allocated to each participant and given to the practice. Each general practice put invitation letters into the corresponding pre-numbered participant pack and completed the mail out.

The study office in Oxford receives returned questionnaires and the coordinating team undertake data quality checks. The returned questionnaires are processed using the electronic data capture software TeleForm Workgroup (Serial Number: 247885; Company name: ePartner Consulting Ltd), which includes internal system validation checks. Once questionnaires are scanned, additional validation is manually completed by a member of the OPAL study team. For example, if a questionnaire is returned with a double-page spread missing, the participant is contacted by telephone with a maximum of two attempts (on two separate days) in order to complete missing sections.

Access to electronic linkage

The majority of OPAL participants (99.2% of those who agreed to participate; n=5,367/5,409) consented for the research team access their UK NHS Digital and primary health care records, and to be approached for future interventional and observational studies. NHS Digital is a national provider

For peer review only - http://bmjopen.bmj.com/site/about/guidelines.xhtml

of information, data and information technology systems for commissioners, analysts and clinicians in health and social care. Information on hospital admissions, outpatient and accident and emergency department visits for individuals receiving NHS hospital treatment in England[36]. Diagnoses are coded using the World Organisation's (WHO) International Classification of Disease version 10 (ICD-10). In addition, date and cause of death of death will be purchased.

Patient and public involvement statement

Patients and the public were involved in the development of the research question, the design of the study, and the conduct of the research. We piloted and refined the OPAL cohort study questionnaires with our Patient and Public Involvement (PPI) representatives including older adults for whom English was a second language in order to ensure acceptability and assist with uptake of the study by ethnic minority groups. We will also collaborate with our PPI representatives when drafting publications and developing a strategy for dissemination to patients and the public.

Ethics

Ethical approval for the study was provided by the London - Brent Research Ethics Committee (16/LO/0348) on 10th March 2016. All participants provided written informed consent, returned with the baseline questionnaire before being enrolled in the study.

Statistical analysis

Descriptive statistics were used to summarize demographic and health-related measures of the OPAL participants at baseline. Selected key demographic and health-related variables are reported in this manuscript.

For peer review only - http://bmjopen.bmj.com/site/about/guidelines.xhtml

To assess whether our cohort is representative of the population of England, we compared characteristics of the OPAL study to those in The English Longitudinal Study of Ageing (ELSA). We deliberately focus on absolute differences and not on statistical significance because the large study samples may produce low p-values even when absolute differences are small. Analyses were performed using STATA software V.15.1 (StataCorp).

The English Longitudinal Study of Ageing (ELSA)

The ELSA study is a prospective study of a representative sample of community-dwelling people aged 50 years or older living in England[37]. It started in 2002 (wave 1), with participants recruited from an annual cross-sectional survey of households who were followed up every two years. For this comparison, we used cross-sectional ELSA data from the core members (n=7,223) at wave eight (May 2016-June 2017), as the time-period was comparable with the OPAL study at baseline. Members aged <65 years (n=2,102) and institutionalized (n=56) were excluded for the comparison. Thus, data from 5,065 ELSA participants were included.

We compared the following participant characteristics between ELSA and OPAL: demographic (age, sex, ethnicity (white vs. non-white), work status (retired vs. non-retired), current relationship status (married vs. non-married), weight, smoking status and health-related self-reported doctor-diagnosed chronic diseases (arthritis, diabetes, heart problems, stroke, dementia, lung disease, osteoporosis and high blood pressure)[38]. We applied the recommended weightings to the data to correct for non-response in ELSA cohort study[39].

Further details of the variables used in OPAL and ELSA cohort studies are in Table S1 supplementary information. The ELSA data management is available in a Stata do-file "Data_management_wave8_Dec2019.do" in supplementary information. The measurement protocol for the ELSA cohort study can be found at http://www.ifs.org.uk/elsa.

For peer review only - http://bmjopen.bmj.com/site/about/guidelines.xhtml

Findings to date

Response

Eight thousand two hundred and forty individuals (64.2% amongst the 12,839 eligible participants) who were sent the invitation letter responded to the invitation, and 65.6% of them (n=5,409/8,240) agreed to participate in the study. Questionnaire response rate (amongst eligible individuals) by practice ranged from 5.1% to 65.8% (median: 45.6%; IQR: 32.2%-54.3%). Lower levels of response were observed in the most deprived practices (Supplementary Table S5)

OPAL baseline data has a low proportion of missing values. The amount of missing data for any single variable varied from 0.2% (n=13/5,409) (for relationship status and current work status) to 6.2% (n=335/5,409) (for Tilburg frailty score (0-15); item missing ranging from 0.4% to 1.9%).

Characteristics of study participants at baseline

The demographic characteristics of participants are reported in Table 2. Half of the participants were women (51.5%; n=2,784/5,409), and the mean (SD) age was 74.9 (6.8) years, ranging from 65 to 100 years. The majority of study participants were white (94.9%; n=5,132/5,409).

The majority of participants were married or partnered (66.6%; n=3,602/5,409), with a higher proportion of women living alone. Most participants were retired (84.8%; n=4,589/5,409), and had secondary school education (56.4%; n= 3,051/5,409). The mean deprivation score of individuals (SD) was 16.6 (14.1) and it was similar between sexes. Women were less likely to report they were current smokers or drinking alcoholic beverages at least once every week than men. Prevalence of overweight and obesity was 38.1% (n=2,061/5,409) and 18.6% (n=1,005/5,409), respectively.

Health-related variables of men and women are described in Table 3 and Figure 2. A high proportion of participants (83.8%; n=4,530/5,409) reported pain in at least one body area in the previous six

For peer review only - http://bmjopen.bmj.com/site/about/guidelines.xhtml

weeks, with pain being more prevalent in women than men (Table 3). Low back pain was the most frequently reported site for pain (44.3%; n=2,397/5,409).

The majority of participants were confident to walk half a mile (66.1%; n=3,577/5,409), with a higher proportion of men being confident walkers. Over thirty-eight percent (n=2,094/5,409) of participants rated their walking speed as strolling at an easy pace or very slow, 18.5% (n=1,002/5,409) reported using a walking aid inside or outside, and 25.5% (n=1,375/5,409) reported that their walking speed to be slower than a year ago. Over a quarter of participants (29.0%; n=1,569/5,409) reported having fallen once or more in the 12 months prior to the baseline questionnaire, and 27.1% (n=1,463/5,409) were frail. Frailty was more prevalent in women. Most of the participants reported good health across four domains of the EQ-5D-5L questions with 88.5% (n=4,784/5,409), 69.7% (n=3,772/5,409), 66.1% (n=3,577/5,409) and 59.0% (n=3,190/5,409) reporting no problems with self-care, anxiety/depression, usual activities and mobility, respectively, except for pain/discomfort with a percentage of participants reporting no problems of 29.5% (n=1,594/5,409). The average HRQoL measured by EQ-5D-5L crosswalk value set and the EQ-VAS were 0.79 (SD 0.20) and 78.4 (SD 17.4), respectively. Women reported worse HRQoL (lower average score in both scales) compared with men (Table 3). The average self-reported EQ VAS score in population norms for UK population aged 65-74 and 75 years and over[40] is broadly comparable to OPAL study (population norm vs. OPAL study: 77.3 vs. 80.5 and 73.8 vs. 75.6, respectively).

The more frequently self-reported health condition was high blood pressure (45.5%; n=2,459/5,409), followed by arthritis (44.2%; n=2,391/5,409) and angina or heart problems (20.2%; n=1,094/5,409). High blood pressure was the most prevalent condition amongst men (47.4%; n=1,244/2,625), and arthritis the most prevalent in women (52.3%; 1,455/2,784) (Figure 2).

For peer review only - http://bmjopen.bmj.com/site/about/guidelines.xhtml

Representativeness of OPAL Cohort study

Supplementary Table S2, S3 and S4 show the sex-specific distribution of characteristics in OPAL and ELSA cohort studies across four age groups. Overall, OPAL participants were broadly comparable with those in the nationally representative ELSA cohort study.

There was a slightly higher proportion of men and a lower proportion of women in the 80 and older age group in OPAL study compared to ELSA study. Both men and women participants in the OPAL study were less likely to smoke and had a lower prevalence of self-reported heart problems, stroke and dementia.

Characteristics of included general practices

General practice area deprivation and the estimated proportion of ethnic groups registered in the practice population are described in Supplementary Table S5. Of the 35 general practices included in the study, 32 had data available on PHE national general practice profiles website. Nine of 32 practices (28.1%) were classified among the most deprived practices (IMD deciles 1-3), 14/32 (43.8%) in the most affluent practices (IMD deciles 8-10) and the remainder categorised as moderate (n=9/32; 28.1%; IMD deciles 4-7).

Cohort multiple randomized controlled trial

The first RCT utilizing the OPAL cohort study is now being undertaken. This trial is testing the effectiveness of a physiotherapist delivered combined physical and psychological intervention for older adults with neurogenic claudication compared to best practice advice (BOOST)[41]. The trial is registered with the International Standard Randomised Controlled Trials database, reference number ISRCTN12698674.

For peer review only - http://bmjopen.bmj.com/site/about/guidelines.xhtml

Strengths and limitations

The original target for recruitment of the OPAL cohort study was a minimum of 4,000 older adults from 32 general practices. However, uptake was better than predicted and we have recruited 5,409 older adults from 35 general practices within nine distinct areas, providing good geographical coverage within England. The wide range of self-report health measures will allow us to account for a large range of potential mediating and confounding variables.

One important limitation of the cohort is the reliance upon self-reported data. However, we have written informed consent to access NHS Digital and primary health care data for the majority of the participants, to allow independent verification of diagnoses related to hospital admission and attendance, and as well as important elements of health service resource use and mortality. Participants living in most deprived neighbourhoods (based on practice deprivation) and non-white ethnicity groups were less likely to participate in OPAL (Supplementary Table S5), but nevertheless our population is broadly representative of the English population.

In terms of the representativeness of the OPAL study, characteristics of OPAL participants are similar to those in the ELSA study (Supplementary Table S2, S3 and S4). The selected variables for the comparison analysis had good comparability in both OPAL and ELSA studies, but there were some differences. For example, in ELSA, weight was calculated using measured weight, whereas in OPAL weight was self-reported. Self-reported weight tends to be underreported, particularly by women and those who are heaviest[42]. In addition, in ELSA, the definition of 'smoker status' and health conditions combines information from previous waves, whereas in OPAL study, only baseline information was used. This may have led to a slight underestimation of the difference between ELSA and OPAL in the percentage of 'ex-smoker' and individuals with the health condition.

For peer review only - http://bmjopen.bmj.com/site/about/guidelines.xhtml

Future work

Data collection for the Year 1 follow-up questionnaire was completed in September 2019 and Year 2 and 3 follow-up will be completed in 2020 and 2021, respectively. We plan to administer questionnaires at annual intervals, and aim to continue this for a minimum of five years.

The potential of this data set has yet to be exploited and further work is in progress. We will start focusing on particular health domains (such as low back pain and mobility problems), together with an exploration of factors underlying the variability of those health domains. Future work will include the development of a prognostic tool to identify older adults at risk of mobility decline to help individuals, GPs and other health professionals identify risk factors and when these should be prioritised as a treatment target. This longitudinal cohort study will also identify health trajectories and will examine their associations with demographic, clinical, and social factors, with the aim of identifying factors that maintain good health and independence in older people.

For peer review only

Where can I find out more?

Further information on the OPAL cohort study can be found on our website: https://www.ndorms.ox.ac.uk/rrio/opal. Data will be available for data sharing. Enquires can be made to Professor Sarah (Sallie) Lamb (Principal Investigator, e-mail: sarah.lamb@ndorms.ox.ac.uk / S.E.Lamb@exeter.ac.uk).

For peer review only

OPAL Study Team

Conway O, Darton F, Dutton S, Garrett A, Hagan D, Haywood D, Hewitt A, Lamb S, Marian I, Morris A, Nevay L, Nicolson P, Sanchez-Santos MT, Slark M, Vadher K, Ward L, Watson M, Williamson E, Arden N, Barker K, Collins G, Fairbank J, Fitch J, French D, Griffiths F, Hanson Z, Hutchinson C, Mallen C, Petrou S.

OPAL General Practice Team

Grange Hill Surgery, Birmingham; Gosford Hill Medical Centre, Oxford; River Brook Medical Centre, Birmingham; The Key Medical Practice, Oxford; Summertown Health Centre, Oxford; Alconbury and Brampton Surgeries, Cambridgeshire; Old Exchange Surgery, Cambridgeshire; The Wand Medical Centre, Birmingham; Kingsfield Medical Centre, Birmingham; Buckden and Little Paxton Surgery, Cambridgeshire; Temple Cowley Medical Group, Oxford; Keynell Covert Surgery, Birmingham; Burbury Medical Centre, Birmingham; Hollow Way Medical Centre, Oxford; Priory View Medical Centre, Leeds; Newton Surgery, Leeds; Priory Fields Surgery, Cambridgeshire, Cromwell Place Surgery, Cambridgeshire; Craven Road Medical Centre, Leeds; Queslett Medical Practice, Birmingham; Hall Street Medical Centre, Saint Helens; Vauxhall Health Centre, Liverpool; Ireland Wood Surgery, Leeds; Civic Medical Centre, Wirral; Brownlow Group Practice, Liverpool; Wareham Surgery, Dorset; The Adam Practice, Poole; The Harvey Practice, Dorset; Three Chequers Medical

For peer review only - http://bmjopen.bmj.com/site/about/guidelines.xhtml

Practice, Salisbury; Gate Medical Centre, Birmingham; Rendcomb Surgery, Cirencester; Cotswold Medical Practice, Cheltenham; Brigstock and South Norwood Partnership, Croydon; Portland Practice, Gloucestershire; Eversley Medical Centre, Croydon.

Supporting NIHR Clinical Research Networks (CRN)

Thames Valley and South Midlands, Eastern; Yorkshire and the Humber, North West Coast; Wessex, West of England; West Midlands, South London.

For peer review only

Competing interests

All authors have completed the Unified Competing Interest form at www.icmje.org/coi_disclosure.pdf. JB reports personal fees from Medtronic Ltd, grants from NIHR HTA Funding, outside the submitted work. CM reports grants from NIHR, during the conduct of the study. SL reports and declared competing interests of authors: Sarah E Lamb was on the Health Technology Assessment (HTA) Additional Capacity Funding Board, HTA End of Life Care and Add-on Studies Board, HTA Prioritisation Group Board and the HTA Trauma Board. All other authors declare no conflicts of interest.

Funding

This research is funded by the NIHR Programme Grants for Applied Research (reference: PTC-RP-PG-0213-20002). Preparatory work for the programme of research was supported by the NIHR Collaborations for Leadership in Applied Health Research and Care (CLAHRC). Christian D. Mallen. is funded by the National Institute for Health Research (NIHR) Applied Research Collaboration (West Midlands), the NIHR School for Primary Care Research and an NIHR Research Professorship in General Practice (NIHR-RP-2014-04-026). Julie Bruce is funded by NIHR Research Capability Funding..

For peer review only - http://bmjopen.bmj.com/site/about/guidelines.xhtml

Disclosure

The views expressed in this article are those of the authors and not necessarily those of the NHS, the NIHR, our funding bodies or the Department of Health and Social Care.

Author contributions

MTSS participated in the data preparation, analysis, and interpretation; and the development and writing of the paper. EW participated in the OPAL study design, data collection and interpretation of the results of the paper. JB, LW and CM participated in the OPAL study design and interpretation of findings. AG and AM participated in the design of the OPAL study, data collection, data preparation and interpretation of findings. SL conceived the study, secured funding, and oversaw all aspects as principal investigator. SL participated in the design and execution of the OPAL study, and the development and writing of the paper. All authors contributed and approved the final manuscript.

Acknowledgments

The authors would like to thank the individuals who participated in The Oxford Pain, Activity and Lifestyle (OPAL) cohort study, the general practitioners and their staff for assisting with the identification and invitation of eligible individuals and the NIHR Biomedical Research Centre, Oxford for funding this study (grant reference PTC-RP-PG-0213-20002).

For peer review only - http://bmjopen.bmj.com/site/about/guidelines.xhtml

References

1. ONS. National population projections: 2016-based: Office for National Statistics; 2017 [Available from: https://www.ons.gov.uk/releases/nationalpopulationprojections2016basedstatisticalbulletin accessed 11/06/2019 2019.
2. Fried LP, Tangen CM, Walston J, et al. Frailty in older adults: evidence for a phenotype. *The journals of gerontology Series A, Biological sciences and medical sciences* 2001;56(3):M146-56. [published Online First: 2001/03/17]
3. Troutman-Jordan M, Staples J. Successful aging from the viewpoint of older adults. *Research and theory for nursing practice* 2014;28(1):87-104. [published Online First: 2014/04/30]
4. Parsons S, Gale CR, Kuh D, et al. Physical capability and the advantages and disadvantages of ageing: perceptions of older age by men and women in two British cohorts. *Ageing Soc* 2012;24(3):452-71. doi: https://doi.org/10.1017/S0144686X12001067
5. Hardy SE, Kang Y, Studenski SA, et al. Ability to walk 1/4 mile predicts subsequent disability, mortality, and health care costs. *Journal of general internal medicine* 2011;26(2):130-5. doi: 10.1007/s11606-010-1543-2 [published Online First: 2010/10/26]
6. Ward RE, Beauchamp MK, Latham NK, et al. A Novel Approach to Identifying Trajectories of Mobility Change in Older Adults. *PloS one* 2016;11(12):e0169003. doi: 10.1371/journal.pone.0169003 [published Online First: 2016/12/23]
7. Woolf AD, Pfleger B. Burden of major musculoskeletal conditions. *Bulletin of the World Health Organization* 2003;81(9):646-56. [published Online First: 2004/01/09]
8. Global, regional, and national disability-adjusted life-years (DALYs) for 359 diseases and injuries and healthy life expectancy (HALE) for 195 countries and territories, 1990-2017: a systematic analysis for the Global Burden of Disease Study 2017. *Lancet* 2018;392(10159):1859-922. doi: 10.1016/s0140-6736(18)32335-3 [published Online First: 2018/11/13]
9. Fayaz A, Croft P, Langford RM, et al. Prevalence of chronic pain in the UK: a systematic review and meta-analysis of population studies. *BMJ open* 2016;6(6):e010364. doi: 10.1136/bmjopen-2015-010364 [published Online First: 2016/06/22]
10. Andorsen OF, Ahmed LA, Emaus N, et al. High prevalence of chronic musculoskeletal complaints among women in a Norwegian general population: the Tromso study. *BMC research notes* 2014;7:506. doi: 10.1186/1756-0500-7-506 [published Online First: 2014/08/12]
11. Eggermont LH, Leveille SG, Shi L, et al. Pain characteristics associated with the onset of disability in older adults: the maintenance of balance, independent living, intellect, and zest in the Elderly Boston Study. *J Am Geriatr Soc* 2014;62(6):1007-16. doi: 10.1111/jgs.12848 [published Online First: 2014/05/16]
12. Leveille SG, Jones RN, Kiely DK, et al. Chronic musculoskeletal pain and the occurrence of falls in an older population. *JAMA* 2009;302(20):2214-21. doi: 10.1001/jama.2009.1738
13. Whitlock EL, Diaz-Ramirez LG, Glymour MM, et al. Association Between Persistent Pain and Memory Decline and Dementia in a Longitudinal Cohort of Elders. *JAMA internal medicine* 2017;177(8):1146-53. doi: 10.1001/jamainternmed.2017.1622 [published Online First: 2017/06/07]
14. Chen Q, Hayman LL, Shmerling RH, et al. Characteristics of chronic pain associated with sleep difficulty in older adults: the Maintenance of Balance, Independent Living, Intellect, and Zest in the Elderly (MOBILIZE) Boston study. *J Am Geriatr Soc* 2011;59(8):1385-92. doi: 10.1111/j.1532-5415.2011.03544.x [published Online First: 2011/08/03]
15. Saraiva MD, Suzuki GS, Lin SM, et al. Persistent pain is a risk factor for frailty: a systematic review and meta-analysis from prospective longitudinal studies. *Age and ageing* 2018;47(6):785-93. doi: 10.1093/ageing/afy104 [published Online First: 2018/07/28]
16. Relton C, Torgerson D, O'Cathain A, et al. Rethinking pragmatic randomised controlled trials: introducing the "cohort multiple randomised controlled trial" design. *BMJ (Clinical research ed)* 2010;340:c1066. doi: 10.1136/bmj.c1066 [published Online First: 2010/03/23]
17. Clegg A, Relton C, Young J, et al. Improving recruitment of older people to clinical trials: use of the cohort multiple randomised controlled trial design. *Age and ageing* 2015;44(4):547-50. doi: 10.1093/ageing/afv044 [published Online First: 2015/04/11]

For peer review only - http://bmjopen.bmj.com/site/about/guidelines.xhtml

18. Bruce J, Lall R, Withers EJ, et al. A cluster randomised controlled trial of advice, exercise or multifactorial assessment to prevent falls and fractures in community-dwelling older adults: protocol for the prevention of falls injury trial (PreFIT). *BMJ open* 2016;6(1):e009362. doi: 10.1136/bmjopen-2015-009362 [published Online First: 2016/01/20]
19. Kuorinka I, Jonsson B, Kilbom A, et al. Standardised Nordic questionnaires for the analysis of musculoskeletal symptoms. *Applied ergonomics* 1987;18(3):233-7. [published Online First: 1987/09/01]
20. Parsons S, Breen A, Foster NE, et al. Prevalence and comparative troublesomeness by age of musculoskeletal pain in different body locations. *Family practice* 2007;24(4):308-16. doi: 10.1093/fampra/cmm027 [published Online First: 2007/07/03]
21. Deyo RA, Battie M, Beurskens AJ, et al. Outcome measures for low back pain research. A proposal for standardized use. *Spine (Phila Pa 1976)* 1998;23(18):2003-13. doi: 10.1097/00007632-199809150-00018 [published Online First: 1998/10/21]
22. Lamb SE, Lall R, Hansen Z, et al. A multicentred randomised controlled trial of a primary care-based cognitive behavioural programme for low back pain. The Back Skills Training (BeST) trial. *Health Technol Assess* 2010;14(41):1-253, iii-iv. doi: 10.3310/hta14410
23. de Schepper EI, Overdevest GM, Suri P, et al. Diagnosis of Lumbar Spinal Stenosis: An Updated Systematic Review of the Accuracy of Diagnostic Tests. *Spine (Phila Pa 1976)* 2013;38(8):E469-E81. doi: 10.1097/BRS.0b013e31828935ac
24. Suri P, Rainville J, Kalichman L, et al. Does this older adult with lower extremity pain have the clinical syndrome of lumbar spinal stenosis? *JAMA* 2010;304(23):2628-36. doi: 10.1001/jama.2010.1833
25. Newell AM, VanSwearingen JM, Hile E, et al. The modified Gait Efficacy Scale: establishing the psychometric properties in older adults. *Physical therapy* 2012;92(2):318-28. doi: 10.2522/ptj.20110053 [published Online First: 2011/11/15]
26. Peel C, Sawyer Baker P, Roth DL, et al. Assessing mobility in older adults: the UAB Study of Aging Life-Space Assessment. *Physical therapy* 2005;85(10):1008-119. [published Online First: 2005/09/27]
27. Lamb SE, Jørstad-Stein EC, Hauer K, et al. Development of a common outcome data set for fall injury prevention trials: the Prevention of Falls Network Europe consensus. *J Am Geriatr Soc* 2005;53(9):1618-22. doi: 10.1111/j.1532-5415.2005.53455.x
28. Gobbens RJ, van Assen MA, Luijkx KG, et al. The Tilburg Frailty Indicator: psychometric properties. *Journal of the American Medical Directors Association* 2010;11(5):344-55. doi: 10.1016/j.jamda.2009.11.003
29. Gobbens RJ, van Assen MA, Luijkx KG, et al. Determinants of frailty. *Journal of the American Medical Directors Association* 2010;11(5):356-64. doi: 10.1016/j.jamda.2009.11.008 [published Online First: 2010/06/01]
30. Herdman M, Gudex C, Lloyd A, et al. Development and preliminary testing of the new five-level version of EQ-5D (EQ-5D-5L). *Qual Life Res* 2011;20(10):1727-36. doi: 10.1007/s11136-011-9903-x [published Online First: 2011/04/12]
31. Janssen MF, Pickard AS, Golicki D, et al. Measurement properties of the EQ-5D-5L compared to the EQ-5D-3L across eight patient groups: a multi-country study. *Qual Life Res* 2013;22(7):1717-27. doi: 10.1007/s11136-012-0322-4 [published Online First: 2012/11/28]
32. van Hout B, Janssen MF, Feng YS, et al. Interim scoring for the EQ-5D-5L: mapping the EQ-5D-5L to EQ-5D-3L value sets. *Value in health : the journal of the International Society for Pharmacoeconomics and Outcomes Research* 2012;15(5):708-15. doi: 10.1016/j.jval.2012.02.008 [published Online First: 2012/08/08]
33. EuroQol Group. EQ-5D 5L | Valuation: Standard value sets [Available from: https://euroqol.org/eq-5d-instruments/eq-5d-5l-about/valuation-standard-value-sets/crosswalk-index-value-calculator/ accessed 17 December 2019.
34. Public Health England National general practice profiles [Available from: http://fingertips.phe.org.uk/profile/general-practice/data accessed August 2019.
35. Department for Communities and Local Government. The English Indices of Deprivation 2015. *In London: Department for Communities and Local Government* 2015

For peer review only - http://bmjopen.bmj.com/site/about/guidelines.xhtml

36. Digital N. Hospital Episode Statistics (HES) [11/06/2019]. Available from: https://digital.nhs.uk/data-and-information/data-tools-and-services/data-services/hospital-episode-statistics.
37. Clemens S, Phelps A, Oldfield Z, et al. English Longitudinal Study of Ageing: Waves 0-8, 1998-2017 2019 [30th Edition:[
38. Steptoe A, Breeze E, Banks J, et al. Cohort profile: the English longitudinal study of ageing. *International journal of epidemiology* 2013;42(6):1640-8. doi: 10.1093/ije/dys168 [published Online First: 2012/11/13]
39. Abell J, Amin-Smith N, Banks J, et al. The dynamics of ageing: evidence from the English Longitudinal Study of Ageing 2002-2016 (Wave 8): The Institute for Fiscal Studies 2018.
40. Janssen B, Szende A. Population Norms for the EQ-5D. In: Szende A, Janssen B, Cabases J, eds. Self-Reported Population Health: An International Perspective based on EQ-5D. Dordrecht (NL): Springer Copyright 2014, The Author(s). 2014:19-30.
41. Williamson E, Ward L, Vadher K, et al. Better Outcomes for Older people with Spinal Trouble (BOOST) Trial: a randomised controlled trial of a combined physical and psychological intervention for older adults with neurogenic claudication, a protocol. *BMJ open* 2018;8(10):e022205. doi: 10.1136/bmjopen-2018-022205 [published Online First: 2018/10/21]
42. Spencer EA, Appleby PN, Davey GK, et al. Validity of self-reported height and weight in 4808 EPIC-Oxford participants. *Public health nutrition* 2002;5(4):561-5. doi: 10.1079/phn2001322 [published Online First: 2002/08/21]
43. Banks J, Nazroo J, Steptoe A, editors. *Evidence from the English Longitudinal Study of Ageing 2002-2012 (Wave 6)*. London: The Institute for Fiscal Studies, 2014.
44. Banks J, Breeze E, Lessof C, et al. Retirement, health and relationships of the older population in England: The 2004 English Longitudinal Study of Ageing. *London: Institute for Fiscal Studies* 2006
45. Mottram S, Peat G, Thomas E, et al. Patterns of pain and mobility limitation in older people: cross-sectional findings from a population survey of 18,497 adults aged 50 years and over. *Qual Life Res* 2008;17(4):529-39. doi: 10.1007/s11136-008-9324-7
46. Driscoll T, Jacklyn G, Orchard J, et al. The global burden of occupationally related low back pain: estimates from the Global Burden of Disease 2010 study. *Annals of the rheumatic diseases* 2014;73(6):975-81. doi: 10.1136/annrheumdis-2013-204631 [published Online First: 2014/03/26]
47. Guralnik JM, Fried LP, Simonsick EM, et al. The Women's Health and Aging Study: Health and Social Characteristics of Older Women with Disability. Bethesda, MD: National Institute of Aging, 1995.
48. Shuval K, Kohl HW, 3rd, Bernstein I, et al. Sedentary behaviour and physical inactivity assessment in primary care: the Rapid Assessment Disuse Index (RADI) study. *British journal of sports medicine* 2014;48(3):250-5. doi: 10.1136/bjsports-2013-092901 [published Online First: 2013/10/23]
49. Rose SB, Elley CR, Lawton BA, et al. A single question reliably identifies physically inactive women in primary care. *The New Zealand medical journal* 2008;121(1268):U2897. [published Online First: 2008/02/08]
50. Buysse DJ, Reynolds CF, 3rd, Monk TH, et al. The Pittsburgh Sleep Quality Index: a new instrument for psychiatric practice and research. *Psychiatry research* 1989;28(2):193-213. [published Online First: 1989/05/01]
51. Collin C, Wade DT, Davies S, et al. The Barthel ADL Index: a reliability study. *International disability studies* 1988;10(2):61-3. [published Online First: 1988/01/01]
52. Gompertz P, Pound, P., & Ebrahim, S. A postal version of the Barthel Index. *Clinical Rehabilitation*, *8*(3), 233-239. *Clinical Rehabilitation* 1994;8(3):233-39.
53. Von Korff M, Ormel J, Keefe F, et al. Grading the severity of chronic pain. *Pain* 1992;50(2):133-49.
54. Syddall HE, Westbury LD, Cooper C, et al. Self-reported walking speed: a useful marker of physical performance among community-dwelling older people? *Journal of the American Medical Directors Association* 2015;16(4):323-8. doi: 10.1016/j.jamda.2014.11.004 [published Online First: 2014/12/20]
55. Chung J, Demiris G, Thompson HJ. Instruments to assess mobility limitation in community-dwelling older adults: a systematic review. *Journal of aging and physical activity* 2015;23(2):298-313. doi: 10.1123/japa.2013-0181 [published Online First: 2014/03/05]

For peer review only - http://bmjopen.bmj.com/site/about/guidelines.xhtml

56. Pinto E, Peters R. Literature review of the Clock Drawing Test as a tool for cognitive screening. *Dement Geriatr Cogn Disord* 2009;27(3):201-13. doi: 10.1159/000203344

57. Laidlaw K, Power MJ, Schmidt S. The Attitudes to Ageing Questionnaire (AAQ): development and psychometric properties. *International journal of geriatric psychiatry* 2007;22(4):367-79. doi: 10.1002/gps.1683 [published Online First: 2006/10/20]

For peer review only

For peer review only - http://bmjopen.bmj.com/site/about/guidelines.xhtml

Tables and Figures

Figure 1. Locations of the areas from which the OPAL Cohort Study was derived. Map of England divided by counties.

Figure 2. Health conditions in men and women of OPAL Cohort Study

For peer review only

For peer review only - http://bmjopen.bmj.com/site/about/guidelines.xhtml

Table 1. Measures included in the OPAL Cohort Study

Data collection for the OPAL Cohort Study		
**Domain measured**	**Self-reported measure**	**Years (Y)**
Socio-demographic	Age, sex, education, relationship status<br>Participation in clubs and groups[43]<br>Requires unpaid/paid carer	Y0-Y5
	Ethnicity<br>Number of live births and stillbirths	Y0
Socio-economic	Participant and GP Area deprivation obtained from postcodes [35]<br>Current work status [44]<br>Type of housing<br>Adequacy of income [45]	Y0-Y5
	Main occupation during lifetime [46] and self-rating of strenuousness of occupation<br>Internet access	Y0
Lifestyle	Weight<br>Alcohol and smoking [47]<br>Current physical activity [48]	Y0-Y5
	Height<br>Lifetime physical activity [49]	Y0
General health data	Self-reported comorbidities and medication use<br>Sleep quality - Pittsburgh Sleep Quality Index [50] and average number of hours sleep each night<br>Incontinence - 2 items from Barthel Index [51 52]<br>Falls in the last 12 months [27]<br>Broken bones or fractures in the last 12 months	Y0-Y5
Musculoskeletal pain	The Nordic pain questionnaire adapted version [19 20]	Y0-Y5
	Report of back pain in last 6 weeks, troublesomeness, onset of back pain and nature of back pain [22]<br>Leg pain and symptoms related to low back pain<br>Screening questions for neurogenic claudication [23]	Y0-Y5
	Report of knee pain, troublesomeness, interference with daily activity [53]	Y1-Y2

For peer review only

For peer review only - http://bmjopen.bmj.com/site/about/guidelines.xhtml

Data collection for the OPAL Cohort Study		
**Domain measured**	**Self-reported measure**	**Years (Y)**
	Location of knee pain	Y1
Mobility	Change in mobility in the last year.<br>Self-rated walking speed [54]<br>Use of walking aids (inside and outside)<br>Mobility concerns<br>Access to transport [43]<br>Life-Space assessment [26]<br>Single item from the Modified Gait Self-Efficacy Scale (10-item) [25]	Y0-Y5
	Difficulty with balance while walking	Y2-Y5
	Difficulties walking a half of mile [55]<br>Difficulties walking up and down a flight of stairs [55]	Y3-Y5
Disability	Self-reported difficulty with Activities of Daily Living (bathing, transfers, toilet use, dressing and eating)	Y2-Y5
Frailty	Tilburg Frailty Index [28 29]	Y0-Y5
Cognition	Clock Drawing Test [56]	Y0-Y5
Beliefs about ageing	Attitude to ageing questionnaire – physical changes subscale [57]	Y0-Y5
Health related quality of life	EuroQol 5-Dimension Health Questionnaire, five-level version [30]<br>EuroQol-Visual Analog Scale (EQ-VAS) [30]	Y0-Y5

For peer review only

For peer review only - http://bmjopen.bmj.com/site/about/guidelines.xhtml

Table 2. Sociodemographic and life-style factors of men and women in the OPAL Cohort Study

Characteristic	Men (n=2,625)	Women (n=2,784)
Age, mean (SD)	74.8 (6.7)	75.0 (6.8)
Age groups, n (%)		
65-69	784 (29.9)	801 (28.8)
70-74	696 (26.5)	734 (26.4)
75-79	542 (20.7)	618 (22.2)
80-84	355 (13.5)	356 (12.8)
85-89	196 (7.5)	203 (7.3)
90+	52 (2.0)	72 (2.6)
Ethnicity (White), n (%)	2,465 (93.9)	2,667 (95.8)
Relationship status, n (%)		
Married/Civil Union	1,897 (72.3)	1,506 (54.1)
Living with Partner	114 (4.3)	85 (3.1)
Unmarried (never married)	117 (4.5)	105 (3.8)
Separated/Divorced	185 (7.1)	273 (9.8)
Widow/Widower	305 (11.6)	809 (29.1)
Live alone, n (%)	534 (20.3)	1,021 (36.7)
Education, n (%)		
High professional or university	1,017 (38.7)	895 (32.2)
Secondary school only	1,370 (52.2)	1,681 (60.4)
None or primary	219 (8.3)	189 (6.8)
Work status (Retired), n (%)	2,187 (83.3)	2,402 (86.3)
Quintiles of IMD, n (%)		
Q1 – Most deprived	293 (11.2)	289 (10.4)
Q2	323 (12.3)	339 (12.2)
Q3	542 (20.7)	613 (22.0)
Q4	575 (21.9)	591 (21.2)
Q5 – Least deprived	892 (34.0)	952 (34.2)
BMI (kg/m^2), mean (SD)	26.8 (4.3)	26.4 (5.3)
Smoking status, n (%)		
Never	1,069 (40.7)	1,608 (57.8)
Ex-Smoker	1,400 (53.3)	1,039 (37.3)
Current	145 (5.5)	118 (4.2)
Cigarettes per day, median (IQR)	15 (10-20)	10 (5-17)
Alcohol intake once per week, n (%)	1,861 (70.9)	1,361 (48.9)

SD=standard deviation; IMD=Index of Multiple Deprivation. Data included older adults 65 years and older at baseline 2016-2018.

For peer review only

Table 3. Health-related characteristics of men and women at the OPAL Cohort Study

Health-related characteristics	Men (n=2,625)	Women (n=2,784)
**Pain in the last 6 weeks, n (%)**		
Low back (small of the back)	1,096 (41.8)	1,301 (46.7)
One of both knees	930 (35.4)	1,123 (40.3)
Wrist/hands	653 (24.9)	1,047 (37.6)
Neck	673 (25.6)	949 (34.1)
Shoulders	665 (25.3)	943 (33.9)
One of both hips/thighs	597 (22.7)	873 (31.4)
One or both ankles/feet	559 (21.3)	752 (27.0)
Upper back	160 (6.1)	344 (12.4)
Elbows	160 (6.1)	171 (6.1)
Any pain, n (%)	2,135 (81.3)	2,395 (86.0)
**Mobility**		
Confidence to walk half a mile, median (IQR)	10 (9-10)	10 (6-10)
Outdoor walking pace, n (%)		
Fast	91 (3.5)	93 (3.3)
Fairly brisk	534 (20.3)	572 (20.6)
Normal	994 (37.9)	958 (34.4)
stroll at an easy pace	647 (24.7)	726 (26.1)
Very slow	326 (12.4)	395 (14.2)
Unable to walk	19 (0.7)	27 (1.0)
Walking rate than 1 year ago, n (%)		
Much better	52 (2.0)	84 (3.0)
Somewhat better	114 (4.3)	101 (3.6)
About the same	1,822 (69.4)	1,831 (65.8)
Somewhat worse	507 (19.3)	622 (22.3)
Much worse	113 (4.3)	133 (4.8)
Walking aid use inside (Yes), n (%)	108 (4.1)	153 (5.5)
Walking aid use outside (Yes), n (%)	306 (11.7)	435 (15.6)
**Falls** in the last year, n (%)		
None	1,900 (72.4)	1,906 (68.5)
One fall	474 (18.1)	624 (22.4)
More than one fall	235 (9.0)	236 (8.5)
**Frailty,** Tilburg frailty score, median (IQR)	2 (1-4)	3 (1-5)
**Quality of life**		
EQ-5D crosswalk index value, mean (SD)	0.79 (0.19)	0.76 (0.21)
EQ-VAS, mean (SD)	79.1 (16.7)	77.7 (18.0)

Sample sizes may vary due to missing values; data included older adults 65 years and older at baseline 2016-2018.

For peer review only - http://bmjopen.bmj.com/site/about/guidelines.xhtml

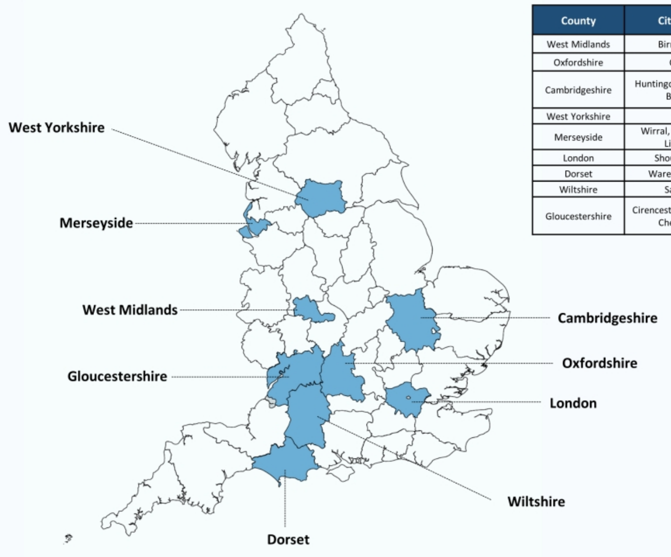

County	City/Town	General practices	No. Invited	No. Participants
West Midlands	Birmingham	8	2,623	754
Oxfordshire	Oxford	5	1,844	905
Cambridgeshire	Huntingdon, Saint Ives, Buckden	5	1,891	1,075
West Yorkshire	Leeds	4	1,782	665
Merseyside	Wirral, Saint Helens, Liverpool	4	1,502	501
London	Shout London	2	652	164
Dorset	Wareham, Poole	3	1,098	547
Wiltshire	Salisbury	1	386	214
Gloucestershire	Cirencester, Northleach, Cheltenham	3	1,061	584
		**35**	**12,839**	**5,409**

Locations of the areas from which the OPAL Cohort Study was derived. Map of England divided by counties

270x160mm (300 x 300 DPI)

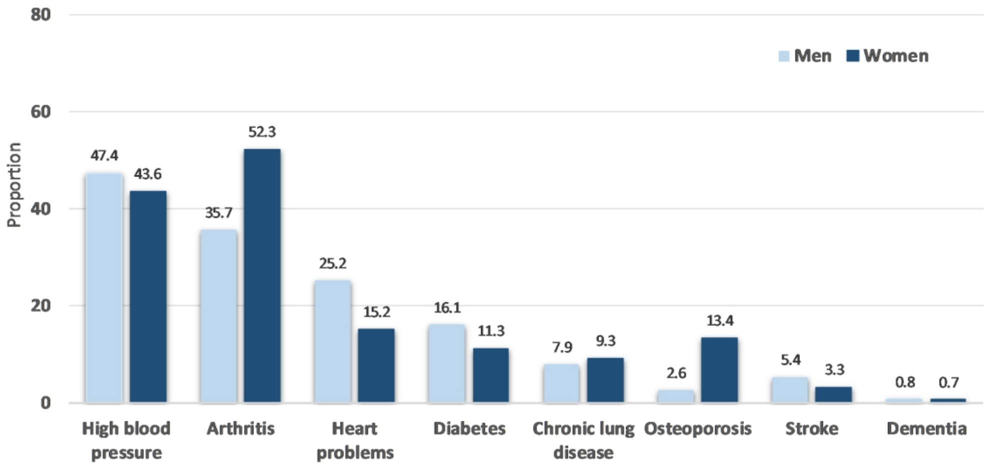

Health conditions in men and women of OPAL Cohort Study

196x101mm (300 x 300 DPI)

Supplementary Data

Supplemental Table S1. Variables used in the OPAL and the ELSA cohort studies.

Variable	Question(s), answer(s) posed to OPAL study	Question(s), answer(s) posed to ELSA study	Name (label) of the variable used for the comparison study
age	Date of birth and date of completion of questionnaire	Age in 5 year bands<br>• 65-69<br>• 70-74<br>• 75-79<br>• 80-84<br>• 85+	'ageg5' (Age variable in 5 year bands)<br>(Derived variable from Institute for fiscal studies (IFS))
Sex	Gender: Male and Female	Sex: Male and Female	'indsex' (Sex variable)
Ethnicity	To which of these ethnic groups do you consider you belong?<br>• White British or white other	To which of the groups on this card do you consider that you belong?<br>• White	'fqethnmr' (Ethnicity recoded into white and non-white (consolidated))
Work status	Which of the following best describes your CURRENT work status?<br>• Retired	Which of the following best describes your CURRENT work status?<br>• Retired	'wpdes' (Best description of current situation)
Relationship status	What is your current relationship status?<br>• Married/Civil Union	What is your current legal marital status?<br>• Married/Civil partner	'dimarr' (Marital status - combined marriage/civil partnership)
Weight	What is your weight?<br>• In Kilograms	Weight measurement<br>• In Kilograms<br>Note: Participants with weight of 37 kg or lower were excluded from the analysis of this variable (n=282) due to the lowest cut-off used in the OPAL cohort study	'estwt' (Final measured or estimated weight (kg))
Smoking status	Which of the following describes your current cigarette smoking status?<br>• Never<br>• Ex-smoker<br>• Current smoker	Smoker status (past or present):<br>• Never<br>• Ex-smoker<br>• Current smoker	'smokerstat' (Derived variable from IFS (non-financial))

For peer review only

**Variable**	**Question(s), answer(s) posed to OPAL study**	**Question(s), answer(s) posed to ELSA study**	**Name (label) of the variable used for the comparison study**
Chronic health conditions	*Has your doctor or nurse ever told you that you have any of the following conditions?*	*Our records show that in the last interview you said that you had been told by a doctor that you had any of the following conditions.*<br><br>*Do you still have the condition?*<br><br>*Since last interview, has a doctor ever told you that you have any of the conditions on this card?*	*Diagnosed last interview AND confirms previous chronic condition*<br><br>*OR*<br><br>*Chronic condition since last interview*
Heart problems	• Angina or heart troubles	• Angina<br>• A heart attack (including myocardial infarction or coronary thrombosis)<br>• Congestive heart failure<br>• A heart murmur<br>• An abnormal heart rhythm<br>• Any other heart trouble	*Angina*: 'hedawan', 'hedacan', 'hediman'<br>*Heart attach*: 'hedawmi', 'hedacmi', 'hedimmi'<br>Congestion heart failure: 'hedawhf', 'hedachf', 'hedimhf'<br>*Heart murmur*: 'hedawhm', 'hedachm', 'hedimhm'<br>*Abnormal heart rhythm*: 'hedawar', 'hedacar', 'hedimar'<br>*Other*: 'hedaw95', 'hedac95', 'hedia95'
Diabetes	• Diabetes (Types I or II)	• Diabetes or high blood sugar	'hedawdi', 'hedacdi', 'hedimdi'
High blood pressure	• High blood pressure	• High blood pressure or hypertension	'hedawbp', 'hedacbp', 'hedimbp'
Stroke	• Stroke	• A stroke (cerebral vascular disease)	'hedawst', 'hedacst', 'hedimst'
Arthritis	• Arthritis	• Arthritis (including osteoarthritis, or rheumatism)	'hedbwar', 'hedbdar', 'hedibar'
Dementia	• Dementia	• Dementia, senility, or any other serious memory impairment	'hedbwde', 'hedbdde', 'hedibde'

For peer review only

Variable	Question(s), answer(s) posed to OPAL study	Question(s), answer(s) posed to ELSA study	Name (label) of the variable used for the comparison study
Osteoporosis	• Osteoporosis	• Osteoporosis, sometimes called thin or brittle bones	'hedbwos', 'hedbdos', 'hedibos'
Chronic lung disease	• Chronic lung disease or Asthma	• Chronic lung disease such as chronic bronchitis or emphysema<br>• Asthma	*Chronic lung disease*: 'hedbwlu', 'hedbdlu', 'hediblu'<br><br>*Asthma*: 'hedbwas', 'hedbdas', 'hedibas'

For peer review only

Supplemental Table S2. Sex-distribution in the OPAL and ELSA cohort studies by age groups

	OPAL (Observed %)				ELSA (Estimated % [95%CI])			
	**65-69**	**70-74**	**75-79**	**80+**	**65-69**	**70-74**	**75-79**	**80+**
Sex								
Female	50.5	51.3	53.3	51.1	51.6 [48.9-54.2]	52.2 [49.4-55.1]	53.8 [50.5-57.0]	58.8 [55.8-61.8]
Male	49.5	48.7	46.7	48.9	48.5 [45.8-51.1]	47.8 [44.9-50.6]	46.2 [43.0-49.5]	41.2 [38.3-44.2]
Unweighted N	1,585	1,430	1,160	1,234	1,547	1,303	992	1,223

ELSA=The English Longitudinal Study of Ageing, a national probability sample of non-institutionalised older people. Wave 8 (2016-2017) was used for this analysis. For variable definitions, see Supplemental Table S1 and for ELSA data management, see Stata do-file "Data_management_wave8_Dec2019.do". Data were weighted to correct for non-response in the ELSA cohort study.

For peer review only

Supplemental Table S3. Characteristics of **women** in the OPAL and ELSA cohort studies by age groups

Characteristics	OPAL (Observed %)				ELSA (Estimated % [95%CI])			
	**65-69**	**70-74**	**75-74**	**80+**	**65-69**	**70-74**	**75-74**	**80+**
Ethnicity, White	95.1	95.5	95.6	97.2	96.7 [95.1-97.8]	97.6 [96.0-98.6]	97.5 [95.0-98.8]	97.3 [95.4-98.5]
Relationship status								
Married/Civil Union	66.0	62.5	52.4	30.7	69.0 [65.8-72.1]	64.6 [60.8-68.2]	54.9 [50.4-59.2]	30.1 [26.6-33.9]
Work status, Retired	75.9	87.3	92.7	91.9	77.3 [74.2-80.0]	88.5 [85.7-90.8]	90.2 [87.3-92.6]	93.2 [91.1-94.9]
Weight (kg), mean (SD)	70.7 (14.8)	69.7 (14.7)	68.4 (13.1)	65.2 (13.0)	73.9 [72.8-75.1]	72.8 [71.5-74.1]	70.3 [69.0-71.6]	66.2 [65.0-67.4]
Smoking status,								
Ex-Smoker	38.3	40.2	35.3	34.7	46.7 [43.3-50.2]	56.8 [52.9-60.7]	49.8 [45.3-54.2]	53.5 [49.5-57.4]
Current	5.2	4.8	5.0	1.6	11.6 [9.5-14.1]	9.1 [7.0-11.8]	7.1 [4.9-10.1]	3.9 [2.6-5.9]
Health conditions,								
Heart problems	8.6	12.4	15.7	27.7	18.5 [16.0-21.4]	21.2 [18.1-24.6]	25.7 [22.0-29.7]	34.3 [30.6-38.2]
Diabetes	8.7	11.3	11.5	14.3	11.4 [9.3-13.8]	14.1 [11.6-17.1]	13.4 [10.6-16.7]	16.9 [14.1-20.2]
High Blood pressure	32.5	42.4	48.7	54.4	36.5 [33.2-39.9]	41.3 [37.5-45.3]	50.7 [46.2-55.1]	57.6 [53.6-61.4]
Stroke	2.1	2.2	2.9	6.7	3.3 [2.3-4.8]	4.3 [2.9-6.2]	7.8 [5.7-10.6]	11.2 [9.0-13.9]
Arthritis	45.6	51.8	55.0	58.6	49.9 [46.4-53.3]	54.3 [50.4-58.2]	58.3 [53.8-62.6]	62.4 [58.4-66.1]
Dementia	0.1	0.3	0.7	1.7	0.3 [0.1-1.1]	1.5 [0.8-2.8]	1.5 [0.7-3.2]	5.7 [4.1-7.8]
Osteoporosis	9.7	11.0	15.2	19.2	13.6 [11.4-16.2]	18.1 [15.2-21.4]	16.8 [13.8-20.4]	21.2 [18.2-24.6]
Chronic lung disease	10.1	9.4	10.2	7.1	6.8 [5.3-8.8]	8.2 [6.3-10.7]	8.7 [6.4-11.7]	6.1 [4.6-8.2]
Unweighted N	801	734	618	631	888	679	534	720

ELSA=The English Longitudinal Study of Ageing, a national probability sample of non-institutionalised older people. Wave 8 (2016-2017) was used for this analysis. For variable definitions, see Supplemental Table S1 and for ELSA data management, see Stata do-file “Data_management_wave8_Dec2019.do”. Data were weighted to correct for non-response in the ELSA cohort study

For peer review only

Supplemental Table S4. Characteristics of **men** in the OPAL and ELSA cohort studies by age groups

Characteristics	OPAL (Observed %)				ELSA (Estimated % [95%CI])			
	**65-69**	**70-74**	**75-74**	**80+**	**65-69**	**70-74**	**75-74**	**80+**
Ethnicity, White	92.9	95.1	93.7	94.0	96.9 [94.8-98.1]	96.2 [93.9-97.6]	97.9 [95.7-99.0]	96.6 [94.3-98.0]
Relationship status								
Married/Civil Union	75.8	75.9	72.1	63.7	76.5 [72.9-79.8]	78.0 [74.3-81.2]	72.6 [68.1-76.7]	64.2 [59.6-68.6]
Work status, Retired	71.1	81.8	90.6	94.5	74.1 [70.5-77.5]	88.0 [85.2-90.4]	93.9 [91.3-95.8]	97.3 [95.4-98.4]
Weight (kg), mean (SD)	85.0 (15.6)	83.7 (15.4)	81.5 (13.8)	78.1 (12.6)	87.3 [86.0-88.7]	84.5 [83.1-85.8]	81.6 [80.3-83.0]	78.6 [77.3-79.8]
Smoking status,								
Ex-Smoker	49.0	54.0	55.4	56.4	61.8 [57.8-65.6]	64.0 [59.9-67.9]	66.9 [62.2-71.3]	75.0 [70.8-78.7]
Current	8.8	5.2	4.6	2.5	9.7 [7.4-12.6]	10.2 [7.7-13.2]	8.2 [5.9-11.4]	2.3 [1.3-4.0]
Health conditions,								
Heart problems	18.4	25.0	27.7	32.2	20.2 [17.1-23.7]	28.5 [24.9-32.4]	35.1 [30.6-39.8]	40.2 [35.8-44.9]
Diabetes	16.1	15.4	16.1	17.1	14.5 [11.8-17.6]	18.0 [14.9-21.5]	19.4 [15.7-23.7]	15.6 [12.5-19.2]
High Blood pressure	44.3	48.7	48.0	49.4	39.5 [35.6-43.5]	47.5 [43.4-51.6]	49.8 [45.0-54.6]	51.9 [47.2-56.5]
Stroke	2.9	4.2	6.6	8.8	5.2 [3.6-7.4]	6.6 [4.8-9.1]	8.7 [6.3-11.7]	16.8 [13.5-20.6]
Arthritis	31.1	32.6	37.5	43.5	31.6 [27.9-35.4]	37.0 [33.1-41.1]	40.6 [36.0-45.4]	41.5 [37.0-46.2]
Dementia	0.4	0.4	0.7	2.0	0.5 [0.2-1.6]	1.7 [0.8-3.3]	2.3 [1.2-4.4]	4.9 [3.3-7.3]
Osteoporosis	1.4	2.3	3.5	3.7	2.0 [1.2-3.5]	5.7 [4.0-8.1]	3.6 [2.1-5.9]	3.5 [2.1-5.7]
Chronic lung disease	7.5	7.8	7.9	8.3	7.4 [5.4-10.0]	10.2 [7.9-13.1]	11.6 [8.8-15.2]	8.1 [5.9-10.9]
Unweighted N	784	696	542	603	659	624	458	503

ELSA=The English Longitudinal Study of Ageing, a national probability sample of non-institutionalised older people. Wave 8 (2016-2017) was used for this analysis. For variable definitions, see Supplemental Table S1 and for ELSA data management, see Stata do-file “Data_management_wave8_Dec2019.do”. Data were weighted to correct for non-response in the ELSA cohort study

For peer review only

Supplemental Table S5. Area deprivation and ethnicity based on each general practice.

General practice	Eligible individuals	%Response rate	Practice IMD 2015 decile (1 More deprived to 10 Least deprived)	Estimated proportion of non-white ethnic groups in practice population		
				%Mixed	%Asian	%Black
OP-01	390	38.5%	-	-	-	-
OP-02	381	54.3%	10	2.3	5.8	1.8
OP-03	400	38.5%	4	4.4	12.8	4.1
OP-04	381	49.1%	10	2.1	5.0	1.7
OP-05	349	54.7%	10	3.4	9.1	1.8
OP-06	396	58.1%	10	1.7	2.1	1.3
OP-07	371	59.8%	10	1.4	3.6	0.0
OP-08	361	23.0%	1	6.9	36.8	21.3
OP-09	385	48.1%	5	4.6	15.7	4.0
OP-10	378	54.8%	10	1.1	1.1	0.0
OP-11	342	44.7%	6	4.3	12.7	6.8
OP-12	295	32.5%	2	4.7	6.1	5.0
OP-13	158	5.1%	1	3.9	62.0	19.1
OP-14	391	42.7%	6	4.4	13.9	6.5
OP-15	356	35.7%	2	2.5	5.9	2.7
OP-16	351	15.4%	1	6.1	31.4	16.7
OP-17	370	54.3%	7	2.2	5.3	2.0
OP-18	376	57.2%	10	1.4	3.7	0.0
OP-19	359	34.5%	5	4.4	10.9	6.3
OP-20	245	22.9%	6	2.2	7.4	3.4
OP-21	386	37.3%	3	0.0	1.1	0.0
OP-22	394	18.0%	1	1.5	2.5	1.7
OP-23/36*	350/366	47.4%/53.0%	8	2.0	3.2	0.0
OP-24	377	46.7%	7	0.0	1.2	0.0
OP-25	345	31.9%	3	3.9	15.2	4.4
OP-26	353	54.4%	8	0.0	0.0	0.0
OP-27	363	44.6%	-	-	-	-
OP-28	382	50.5%	10	0.0	1.2	0.0
OP-29	396	55.4%	9	1.0	1.8	0.0
OP-30	389	5.7%	-	-	-	-
OP-31	342	65.8%	8	0.0	1.1	0.0
OP-32	359	53.5%	9	0.0	0.0	0.0
OP-33	351	22.5%	4	7.2	26.9	33.8
OP-34	360	46.4%	8	1.4	2.8	0.0
OP-35	301	28.2%	3	7.2	29.6	28.2

IMD=Index of Multiple deprivation. 8 general practices had a response rate below (red) and 13 above (green) to the expected rate (<30% and >50%, respectively). Information found on the following government website: https://fingertips.phe.org.uk/profile/general-practice. *Two different random samples of individuals were selected from the same general practice.

For peer review only

BMJ Open

Cohort profile: Oxford Pain, Activity and Lifestyle (OPAL) study, a prospective cohort study of older adults in England

Journal:	*BMJ Open*
Manuscript ID	bmjopen-2020-037516.R1
Article Type:	Cohort profile
Date Submitted by the Author:	25-Jun-2020
Complete List of Authors:	Sanchez Santos, Maria ; University of Oxford, Centre for Rehabilitation Research in Oxford, Nuffield Department of Orthopaedics, Rheumatology and Musculoskeletal Sciences<br>Williamson, Esther; University of Oxford, Centre for Rehabilitation Research in Oxford, Nuffield Department of Orthopaedics, Rheumatology and Musculoskeletal Sciences<br>Bruce, Julie; University of Warwick, Warwick Clinical Trials Unit<br>Ward, Lesley; University of Oxford, Centre for Rehabilitation Research in Oxford, Nuffield Department of Rheumatology, Orthopaedics and Musculoskeletal Sciences; Northumbria University, Department of Sport, Exercise & Rehabilitation<br>Mallen, Christian; Keele University, Arthritis Research UK Primary Care Centre<br>Garrett, Angela; University of Oxford, Centre for Rehabilitation Research in Oxford, Nuffield Department of Orthopaedics Rheumatology and Musculoskeletal Sciences<br>Morris , Alana ; University of Oxford, Centre for Rehabilitation Research in Oxford, Nuffield Department of Orthopaedics Rheumatology and Musculoskeletal Sciences<br>Lamb, Sarah; University of Exeter, College of Medicine and Health; University of Oxford, Centre for Rehabilitation Research in Oxford, Nuffield Department of Orthopaedics Rheumatology and Musculoskeletal Sciences
**Primary Subject Heading**:	Epidemiology
Secondary Subject Heading:	Geriatric medicine
Keywords:	EPIDEMIOLOGY, GERIATRIC MEDICINE, Musculoskeletal disorders < ORTHOPAEDIC & TRAUMA SURGERY, PRIMARY CARE

SCHOLARONE™
Manuscripts

I, the Submitting Author has the right to grant and does grant on behalf of all authors of the Work (as defined in the below author licence), an exclusive licence and/or a non-exclusive licence for contributions from authors who are: i) UK Crown employees; ii) where BMJ has agreed a CC-BY licence shall apply, and/or iii) in accordance with the terms applicable for US Federal Government officers or employees acting as part of their official duties; on a worldwide, perpetual, irrevocable, royalty-free basis to BMJ Publishing Group Ltd ("BMJ") its licensees and where the relevant Journal is co-owned by BMJ to the co-owners of the Journal, to publish the Work in this journal and any other BMJ products and to exploit all rights, as set out in our licence.

The Submitting Author accepts and understands that any supply made under these terms is made by BMJ to the Submitting Author unless you are acting as an employee on behalf of your employer or a postgraduate student of an affiliated institution which is paying any applicable article publishing charge ("APC") for Open Access articles. Where the Submitting Author wishes to make the Work available on an Open Access basis (and intends to pay the relevant APC), the terms of reuse of such Open Access shall be governed by a Creative Commons licence – details of these licences and which Creative Commons licence will apply to this Work are set out in our licence referred to above.

Other than as permitted in any relevant BMJ Author's Self Archiving Policies, I confirm this Work has not been accepted for publication elsewhere, is not being considered for publication elsewhere and does not duplicate material already published. I confirm all authors consent to publication of this Work and authorise the granting of this licence.

For peer review only

Cohort profile: Oxford Pain, Activity and Lifestyle (OPAL) study, a prospective cohort study of older adults in England

Maria T Sanchez-Santos[1], Esther Williamson[1], Julie Bruce[2], Lesley Ward[1,3], Christian D. Mallen[4], Angela Garrett[1], Alana Morris[1], Sarah E Lamb[1,5] on behalf of the OPAL study team

Authors' affiliations:

[1]Centre for Rehabilitation Research, Nuffield Department of Rheumatology, Orthopaedics and Musculoskeletal Sciences, University of Oxford, Oxford, United Kingdom (UK)

[2]Warwick Clinical Trials Unit, Division of Health Sciences, University of Warwick, Coventry, UK

[3]Department of Sport, Exercise & Rehabilitation, Northumbria University, Newcastle-upon-Tyne

[4]School of Primary, Community and Social Care, Keele University, Staffordshire

[5]College of Medicine and Health, University of Exeter, UK

Corresponding Author: Sarah E Lamb

Email: S.E.Lamb@exeter.ac.uk

Tel: +44(0)1392 722 370

Heavitree Road, Exeter, Devon, EX1 2LU

Words: Abstract: 283 ; Main text: 4,836; **Tables:** 3; **Figures:** 2

Supplementary files: 2; **References**: 68

For peer review only

For peer review only - http://bmjopen.bmj.com/site/about/guidelines.xhtml

Abstract

PURPOSE: The 'Oxford Pain, Activity and Lifestyle' (OPAL) cohort is a longitudinal, prospective cohort study of adults, aged 65 years and older, living in the community which is investigating the determinants of health in later life. Our focus was on musculoskeletal pain and mobility, but the cohort is designed with flexibility to include new elements over time. This paper describes the study design, data collection, and baseline characteristics of participants. We also compared the OPAL baseline characteristics with nationally representative data sources.

PARTICIPANTS: We randomly selected eligible participants from two stratified age bands (65-74 and 75 and over years). In total, 5,409 individuals (42.1% of eligible participants) from 35 general practices in England agreed to participate between 2016 and 2018. The majority of participants (n=5,367) also consented for research team to access their UK NHS Digital and primary health care records.

FINDINGS TO DATE: Mean participant age was 74.9 years (range 65-100); 51.5% (n=2,784/5,409) were women. 94.9% of participants were white, and 28.8% lived alone. Over 83.0% reported pain in at least one body area in the previous six weeks. Pain was more prevalent in women (86.0%). One third of participants reported having one or more falls in the last year. Most participants were confident in their ability to walk outside. Characteristics of OPAL cohort participants were broadly similar to the general population of the same age.

FUTURE PLANS: Postal follow-up of the cohort is being undertaken at annual intervals, with data collection ongoing. Linkage to NHS hospital admission data is planned. This English prospective cohort offers a large and rich resource for research on the longitudinal associations between demographic, clinical, and social factors and health trajectories and outcomes in community-dwelling older people.

For peer review only - http://bmjopen.bmj.com/site/about/guidelines.xhtml

Strengths and limitations of this study

- OPAL is a new, high quality cohort of older community-dwelling people aiming to explore causes and consequences of pain, frailty, mobility decline, disability and poor health-related quality of life.
- A total of 5,409 older adults from 35 general practices in nine distinct areas in England participated at baseline, 2016-2018.
- OPAL participants are similar to those in general population of the same age
- The cohort study relies on self-reported and routine NHS data, there is not face to face data collection.
- Our findings may under represent older people living in the community with severe cognitive impairment.

For peer review only

Introduction

The population of the United Kingdom (UK) is undergoing a fundamental change in its age structure, due to lower birth rates and extended life expectancy. One in four people in the UK are projected to be aged 65 or over by 2050, with 15% aged over 75 years and 5% aged 85 years or older[1]. This change reflects gains in health and social development, and it is important that as many years of life are spent in good health as possible.

Active independence is one of the key concerns of older people, and mobility is critically important for independence[2 3]. Older people value their mobility highly and consider mobility loss as a key disadvantage of aging[4]. Poor or limited mobility is linked to functional decline, mortality, and increased health care utilization[5]. Conceptually, factors associated with mobility decline precede disability within models of disablement. Therefore, identification of factors associated with mobility decline are important for prevention of, and rehabilitation from, mobility decline[6].

Musculoskeletal pain is one of the leading causes of disability and disease burden worldwide among community-dwelling older adults[7 8]. A recent review estimated that the prevalence of chronic pain among older adults in the UK ranged from 42% in 65-74 years old to 62% in the over 75 age group[9]. These prevalence estimates are similar to other developed countries[10].

Musculoskeletal pain has a large impact on many other aspects of older people's health such as loss of mobility, frailty, cognitive impairment, falls, and poor sleep quality[11-15]. However, the role of musculoskeletal pain on adverse health outcomes in older adults is poorly understood. The majority of studies are cross-sectional in design, thus are limited; and only few longitudinal studies have examined potential mediators between pain and disability[16]. A better understanding of the causal path between musculoskeletal pain and disability in representative community-based older adults is needed to inform decisions about treatment and rehabilitation.

For peer review only - http://bmjopen.bmj.com/site/about/guidelines.xhtml

There are a number of high quality cohort studies examining age-related health conditions among community dwelling older adults. These include the English Longitudinal Study of Ageing, the MOBILIZE Boston Study, the Longitudinal Study of Ageing, the Baltimore Longitudinal Study of Aging and the Italian Invecchiare aging in Chianti study (InChianti), amongst many others. However, to our knowledge, only one cohort focuses on the impact and contribution of musculoskeletal pain on disability in older people, the ongoing MOBILIZE Boston Study[17]. This American cohort is limited by a relatively small sample size (765 participants at inception).

In order to address these knowledge gaps, we assembled the Oxford Pain, Activity and Lifestyle (OPAL) cohort, a prospective study of community dwelling older adults from across England. The immediate objectives were:

- To investigate the causes and consequences of mobility decline and disability in later life, and the role and contribution of musculoskeletal pain and other factors;
- To develop a prognostic tool to assess mobility decline in a population-based cohort of older adults in UK;
- To investigate factors that moderate or mediate the effects of musculoskeletal pain on health outcomes. For example, we will investigate whether specific social, physical and psychological factors play an intermediate role between low back pain and mobility decline.

In addition, we intend to use the OPAL cohort to identify potential participants for future clinical trials of disability prevention in later life and to study disablement and multi-morbidity more broadly. The 'cohort multiple randomised controlled trials' study design is becoming increasingly common[18 19]. The concept is to use data collected from an established cohort to identify people with specific health conditions and then, as and when the opportunity arises, invite them to participate in a clinical trial relevant to their condition.

For peer review only - http://bmjopen.bmj.com/site/about/guidelines.xhtml

In this paper, we describe the OPAL cohort, design, data collection, and the profile of study participants at baseline and their overall representativeness of the English general population.

Cohort description

Study design

A population-based, longitudinal, prospective cohort study in England, using a combination of annually administered, self-reported questionnaires and routinely collected health data.

Practice and participant identification

General practice identification

General practices who were working with the NIHR Clinical Research Network (CRN), which have been shown to be generalisable to wider primary care community[20], were approached to take part in the study. In terms of geographical spread, we included a range of rural and urban areas across England, to capture diversity in both socioeconomic and ethnic profiles.

Participant identification

Eligible participants were identified from electronic record searches of general practice lists. A random sample of approximately 400 individuals (median: 365; range 158-400) per practice was selected (Figure 1). To ensure an equal representation in two age bands: 65-74 years and 75 years and over, around 200 individuals per practice within each age group were randomly selected.

Inclusion criteria

People registered with a general practice, aged 65 years and older, and living in the community, including sheltered or supported housing, were eligible for invitation.

For peer review only

For peer review only - http://bmjopen.bmj.com/site/about/guidelines.xhtml

Exclusion criteria

Individuals were excluded if they lived in a residential care or nursing home. Following the generation of the random sample, a designated General Practitioner (GP) or research nurse from each practice screened the list to exclude those with known terminal illness with a life expectancy of less than six months, those who presented with severe health or social concerns sufficient to preclude approach, or those considered unable to provide informed consent.

Sample size

The sample size was determined by the prevalence of lower back pain and musculoskeletal problems in older people and driven by the sample size requirement for the prognostic tool to assess mobility decline. We pre-specified a minimum of 1,000 participants of the sample should have lower back pain as this would be sufficient for a range of epidemiological analyses, including predictive modelling, within sub-sample of people with lower back pain[21 22]. The Cambridge Cohort Study of Ageing[23] provided the most recent estimates of disabling low back pain in the population aged 70 to 90 years, with prevalence of 25% to 30% for these age groups respectively. If we assume that 25% of people aged over 65 years have low back pain, then we required a minimum of 4,000 people to yield 1,000 with low back pain and 3,000 people without low back pain. We estimated that between 30-40% of participants would agree to participate based on uptake to the Prevention of Falls Injury Trial (PreFIT)[24] which recruited an older population into an English falls prevention study and anticipated that there would be attrition from the sample over time. Therefore, we had to approach a minimum of 11,000 people, or approximately 350 people from each of 32 practices across our regions to achieve our recruitment target.

For peer review only - http://bmjopen.bmj.com/site/about/guidelines.xhtml

Recruitment and enrolment

Recruitment and enrolment to OPAL commenced in October 2016 and completed in September 2018. A total of 12,839 individuals from thirty-five general practices in nine different areas of England were invited to take part (Figure 1). A pack including an invitation letter, participant information leaflet, consent form, baseline questionnaire, and a postage paid return envelope was sent by the general practice. Five thousand four hundred and nine (42.1% of those eligible; range 5.1%-65.8% across practices) individuals who returned the baseline questionnaire and a signed consent form to the University of Oxford study office were enrolled in the study (Figure 1). One-fifth (21.3% of those eligible; n=2,736/12,839) declined participation and 4,694 (36.6%; n=4,694/12,839) did not respond. Non-responders were sent one postal reminder, four weeks after the original invitation. If no response was received, no further contact was made. The flow chart of the sample is illustrated in Figure S1 supplementary information.

How often are participants followed up?

Study participants are followed up by postal questionnaire at annual intervals for five years. First year follow up is completed, second and third year follow-up will complete in September 2020 and 2021, respectively. Future follow-up questionnaires will be sent at four and five years from the date of the original invitation.

What is being measured?

Postal self-completed questionnaire

The OPAL cohort study includes information on a range of domains including demographic, socioeconomic, lifestyle variables, social participation, attitudes to ageing, musculoskeletal pain, health-related factors, comorbidity, mobility, disability, frailty, cognitive function, health-related quality of life, and medications (see Table 1).

For peer review only - http://bmjopen.bmj.com/site/about/guidelines.xhtml

Musculoskeletal symptoms is assessed by asking the participant if they have experienced any trouble (ache, pain or discomfort) in nine different body sites (knees, hands/wrists, neck, shoulders, hips, feet/ankles, elbows, lower and upper back) during the last six weeks[25 26]. Information on presence, frequency, troublesomeness, onset, and description of back pain in the last six weeks was collected using recognised methods[26-28]. Information about the spread of back related symptoms was also included. To identify individuals with possible spinal stenosis we asked participants their pain travelled into their buttocks/legs, whether it was exacerbated while standing up or walking and whether the symptoms improved when sitting down or bending forward[29 30]. *Mobility* was assessed using different measures. Confidence to walk a half a mile was assessed using a single item from the Modified Gait Self-efficacy scale which is rated on a 1 'not confident at all' to 10 "totally confident" scale[31]. Participants also reported their perceived usual walking pace outdoors with six possible responses: "Unable to walk", "very slow", "stroll at an easy pace", "normal", "fairly brisk" and "fast". Change in mobility in the last year was measured with the question "Compared with 1 year ago, how would you rate your walking in general?" (Response options: much better, somewhat better, about the same, somewhat worse or much worse than a year ago). Participant, family, friends or doctor's concerns about participant ability to walk and move around was measured using two questions. Potential responses were "Extremely", "A little concerned" or "Not concerned at all". Life-space mobility was measured using five questions from the life-space assessment (LSA) questionnaire [32]: ''During the past 4 weeks have you gone to: (1) other rooms in your home besides the room where you sleep? (2) An area outside of your home as your porch, deck or patio, hallway or garage? (3) Different places in your neighbourhood? (4) Locations outside of your neighbourhood, but within your city? and (5) places outside your town?'. *Falls* data were collected as recommended by the Prevention of Falls Network Europe, using a single question , "In the last 12 months, have you had any fall including a slip or trip following which you have come to rest on the ground, floor or lower

For peer review only - http://bmjopen.bmj.com/site/about/guidelines.xhtml

level?"[33]. Three possible responses were available: not fallen, fallen once or more than once in the last year. *Frailty* was measured by The Tilburg Frailty Indicator (TFI)[34 35], which is composed of two parts. The first part describes different determinants of frailty based on sociodemographic data and health-related questions. The second part contains 15 items which measure three frailty domains: physical (8 items), psychological (4 items) and social (3 items). Frailty total scale and individual domain scores are derived from the second part. All items are rated as a binary response of either 0 or 1. Scores are the sum of the respective item points with a total score ranged from 0 to 15, with higher scores representing more frailty. A total score ≥5 points indicates that the individual is frail[34].

Cognitive function was measured with a clock-drawing test[36]. Participants were asked to draw the entire face of a clock depicting the time "10 minutes after 11" following the instructions given in the questionnaire. Scoring was a six-point system according to visual-spatial aspects and the correct denotation of time: normal cognition (score 6); minor visuospatial errors (score 5); mild (score 4), moderate (score 3) or severe (score 2) visuospatial disorganisation of time, or no reasonable representation of a clock (score 1).

Health-Related Quality of life (HRQoL) was measured by the EuroQol-5D-5L (EQ-5D-5L) questionnaire, a generic measure of HRQoL that includes five levels of functioning from level 1 (no problems) to level 5 (severe or extreme problems)[37 38]. Additionally, respondents rated their current health status according to the EuroQol-Visual Analog Scale (EQ-VAS), from 0 (worst imaginable health) to 100 (best imaginable health). The responses from the five domains were converted into a single EQ-5D index value using the EQ-5D-5L Crosswalk Index Value Calculator to produce a final QoL value[39 40]. The index values ranged between -0.594 (a state worse than death) to 1 (best possible health state).

New variables have been added to follow up questionnaires (Table 1), allowing the cohort to be used for a wider range of analytical approaches and purposes, and to dovetail to recruitment of new

For peer review only - http://bmjopen.bmj.com/site/about/guidelines.xhtml

clinical trials. The first follow up (Year 1) repeated baseline variables (Table 1) with the exception of ethnicity, number of children, height, education, lifetime physical activity, main occupation during lifetime, self-rating of strenuousness of occupation, and use of smart-phone or computer to access the internet. Added variables included presence, frequency, troublesome, location and description of knee pain. The second wave of follow-up of data collection is collecting variables included in previous wave (Year 1) in addition to difficulty balancing whilst walking and any difficulty in the following basic activities of daily living (ADL); bathing, transfers, toilet use, dressing and eating. Each activity will be rated from 'no difficulty' to 'Unable to perform'.

Characteristics of participating general practices

General practice deprivation and estimated proportion of non-white ethnic groups in the practice population were obtained from Public Health England (PHE)[41]. Deprivation was measured by the Index of Multiple Deprivation 2015 (IMD2015)[42]. Practice IMD scores are practice population weighted based on the Lower Layer Super Output Areas (LSOAs) where the practice population resides. LSOA is a low-level geography designed to contain 1,500 inhabitants on average. Following the 2011 census, there were 32,844 English LSOAs.

General practice urbanity was defined using the 2011 urban-rural classification[43]. Within this classification, any settlement with a population of 10,000 people or more is defined as urban, with all others are classified as rural. It was determined at the LSOA level. Each general practice postcode was linked to its LSOA and it was then matched to urbanity[44].

Data management and quality control

All data are being processed and stored according to the Data Protection Act 2018. As the OPAL study pre-dated General Data Protection Regulation (GDPR) 2018, all participants were sent an updated GDPR statement along with their next annual questionnaire.

For peer review only - http://bmjopen.bmj.com/site/about/guidelines.xhtml

A software application was developed to support the filtering and random sampling of individuals from the practice lists. Identifiable data were removed by the application. When eligible participants were selected, a unique screening number was allocated to each participant and given to the practice. Each general practice put invitation letters into the corresponding pre-numbered participant pack and completed the mail out.

The study office in Oxford receives returned questionnaires and the coordinating team undertake data quality checks. Returned questionnaires are processed using the electronic data capture software TeleForm Workgroup (Serial Number: 247885; Company name: ePartner Consulting Ltd), which includes internal system validation checks. Once questionnaires are scanned, additional validation is manually completed by a member of the OPAL study team. For example, if a questionnaire is returned with a double-page spread missing, the participant is contacted by telephone with a maximum of two attempts (on two separate days) to complete missing sections.

Access to electronic linkage

The majority of OPAL participants (99.2% of those who agreed to participate; n=5,367/5,409) consented for the research team access their UK NHS Digital and primary health care records, and to be approached for future interventional and observational studies (Up to date, data linkage are not completed). NHS Digital is a national provider of information, data and information technology systems for commissioners, analysts and clinicians in health and social care. The database holds information on hospital admissions, outpatient and accident and emergency department visits for individuals receiving NHS hospital treatment in England[45]. Diagnoses are coded using the World

For peer review only - http://bmjopen.bmj.com/site/about/guidelines.xhtml

Organisation's (WHO) International Classification of Disease version 10 (ICD-10). In addition, date and cause of death of death will be purchased/linked to NHS Digital.

Patient and public involvement statement

Patients and the public were involved in the development of the research question, the design of the study, and the conduct of the research. We piloted and refined the OPAL cohort study questionnaires with our Patient and Public Involvement (PPI) representatives. Our PPI group included older adults for whom English was a second language in order to ensure acceptability of wording of materials and to assist with uptake of the study by ethnic minority groups. We will continue to collaborate with our PPI representatives when drafting publications and with dissemination of findings to patients and the public.

Ethics

Ethical approval for the study was provided by the London - Brent Research Ethics Committee (16/LO/0348) on 10th March 2016. All participants provided written informed consent, returned with the baseline questionnaire before being enrolled in the cohort study.

Statistical analysis

Descriptive statistics were used to summarize demographic and health-related measures of the OPAL participants at baseline. Selected key demographic and health-related variables are reported in this manuscript.

To assess whether our cohort is representative of the population of England, we compared a range of demographic and health-related characteristics of the OPAL cohort study with the 2011 England

For peer review only - http://bmjopen.bmj.com/site/about/guidelines.xhtml

Census[46] and with The English Longitudinal Study of Ageing (ELSA) cohort[47]. We deliberately focus on absolute differences and not on statistical significance because the large study samples may produce low p-values even when absolute differences are small. Analyses were performed using STATA software V.15.1 (StataCorp).

The English Longitudinal Study of Ageing (ELSA)

The ELSA study is an ongoing prospective cohort study of a representative sample of community-dwelling people aged 50 years or older living in England[47]. It started in 2002 (wave 1), with participants recruited from an annual cross-sectional survey of households who were then followed up every two years. For this comparison, we used cross-sectional ELSA data from the core members (n=7,223) at wave eight (May 2016-June 2017), as the time-period was comparable with the OPAL study on recruitment. ELSA participants aged <65 years (n=2,102) and institutionalized (n=56) were excluded for the comparison. Thus, data from 5,065 ELSA participants were included.

We compared the following participant characteristics between ELSA and OPAL: work status (retired vs. non-retired), current relationship status (married vs. non-married), weight, smoking status and health-related self-reported doctor-diagnosed chronic diseases (arthritis, diabetes, heart problems, stroke, dementia, lung disease, osteoporosis and high blood pressure)[48]. We applied the recommended weightings to the data to correct for non-response in ELSA cohort study[49].

Further details of the variables used in OPAL and ELSA cohort studies are in Table S1 supplementary information. The ELSA data management is available in a Stata do-file "Data_management_wave8_Dec2019.do" in supplementary information. The measurement protocol for the ELSA cohort study can be found at http://www.ifs.org.uk/elsa.

For peer review only - http://bmjopen.bmj.com/site/about/guidelines.xhtml

Dealing with missing data

Bias due to missing data (and the mechanism causing the data to be missing) will be investigated and an appropriate analysis approach, such as multiple imputation and/or inverse-probability weighting, to manage this problem will be used depending of the type of study being analysed. Only observed characteristics of OPAL participants at baseline are shown in this manuscript.

Findings to date

Response to invitation to participate

Eight thousand one hundred and forty-five individuals (63.4% amongst the 12,839 eligible participants) who were sent the invitation letter responded to the invitation, 5,409 individuals (65.6% amongst the 8,240 responders) agreed to participate in the study and 2,736 individuals declined to participated (Supplementary Figure S1).

Age and sex distribution of participants and non-participants (declined and non-responders) are shown in Supplementary Table S2, and by general practice in Supplementary Figure S2-S3. Overall, the participation rate in the OPAL cohort study was lower in the oldest age group (participation rates were over 40% for those aged 65-79 years and 36% for those aged 80+ years, respectively), although these were within the expected response rate. Response rate was similar between sexes (participation rates were 44.2% and 42.8% in men and women, respectively). No differences between participants and non-participants in terms of age or sex was observed, and these results were consistent across most practices.

Questionnaire response rate (amongst eligible individuals) by practice ranged from 5.1% to 65.8% (median: 45.6%; IQR: 32.2%-54.3%). Lower levels of response were observed in the most deprived practices (Supplementary Table S3)

For peer review only - http://bmjopen.bmj.com/site/about/guidelines.xhtml

OPAL baseline data has a low proportion of missing values. The amount of missing data for any single variable varied from 0.2% (n=13/5,409) (for relationship status and current work status) to 5.9% (n=321/5,409) (for Tilburg frailty score (0-15); item missing ranging from 0.4% to 1.9%).

Characteristics of OPAL study participants at baseline

The demographic characteristics of participants are reported in Table 2. Half of the participants were women (51.5%; n=2,784/5,409), and the mean (SD) age was 74.9 (6.8) years, ranging from 65 to 100 years. The majority of study participants were white (94.9%; n=5,132/5,409).

The majority of participants were married or partnered (66.6%; n=3,602/5,409), with a higher proportion of women living alone. Most participants were retired (84.8%; n=4,589/5,409), and had secondary school education (56.4%; n= 3,051/5,409). The median (IQR) area deprivation score of participants was 12.5 (6.9-20.3) and it was similar between sexes. In England the median (IQR) deprivation score is 17.4 (9.7-30.1). Women were less likely to report they were current smokers or drinking alcoholic beverages at least once every week than men. Prevalence of overweight (BMI: 25-29.9 kg/m^2) and obesity (BMI: ≥30 kg/m^2) amongst the whole sample was 38.1% (n=2,061/5,409) and 18.6% (n=1,005/5,409), respectively.

Health-related variables of men and women are described in Table 3 and Figure 2. A high proportion of OPAL participants (84.0%; n=4,543/5,409) reported musculoskeletal symptoms in at least one body area in the previous six weeks, with symptoms being more prevalent in women than men (Table 3). Low back pain was the most frequently reported site for pain (44.4%; n=2,404/5,409).

The majority of participants were mobile and were confident to walk half a mile (66.1%; n=3,577/5,409), with a higher proportion of men being confident walkers. Over one-third (38.7%; n=2,094/5,409) of participants rated their walking speed as strolling at an easy pace or very slow, 18.5% (n=1,002/5,409) reported using a walking aid inside or outside, and 25.5% (n=1,375/5,409)

For peer review only - http://bmjopen.bmj.com/site/about/guidelines.xhtml

363 reported that their walking speed to be slower than a year ago. Over a quarter of participants (29.0%;
364 n=1,569/5,409) reported having fallen once or more in the 12 months prior to the baseline
365 questionnaire, and 27.1% (n=1,463/5,409) were classified as frail. Frailty was more prevalent in
366 women. The majority of study participants presented high cognitive function, with 82.8%
367 (n=4,481/5,409) of participants having a score of 5 or 6 points in the clock-drawing test. Most of the
368 participants reported good health across four domains of the EQ-5D-5L questions with 88.5%
369 (n=4,784/5,409), 69.7% (n=3,772/5,409), 66.1% (n=3,577/5,409) and 59.0% (n=3,190/5,409)
370 reporting no problems with self-care, anxiety/depression, usual activities and mobility, respectively,
371 except for pain/discomfort with a percentage of participants reporting no problems of 29.5%
372 (n=1,594/5,409). The average HRQoL measured by EQ-5D-5L crosswalk value set and the EQ-VAS
373 were 0.79 (SD 0.20) and 78.4 (SD 17.4), respectively. Women reported worse HRQoL (lower average
374 score in both scales) compared with men (Table 3). The average self-reported EQ VAS score in
375 population norms for UK population aged 65-74 and 75 years and over[50] is broadly comparable to
376 the OPAL study (population norm vs. OPAL study: 77.3 vs. 80.5 and 73.8 vs. 75.6, respectively).

377

378 The more frequently self-reported health condition was high blood pressure (45.5%; n=2,459/5,409),
379 followed by arthritis (44.2%; n=2,391/5,409) and angina or heart problems (20.2%; n=1,094/5,409).
380 High blood pressure was the most prevalent condition amongst men (47.4%; n=1,244/2,625), and
381 arthritis the most prevalent in women (52.3%; 1,455/2,784) (Figure 2).

382

383 Representativeness of OPAL Cohort study

384 Demographic characteristics in OPAL cohort study were similar to the general population of the same
385 age range in the 2011 England Census (Supplementary Table S4). There was a lower proportion of
386 women in the 80 and older age group in OPAL study compared to the general population.

For peer review only - http://bmjopen.bmj.com/site/about/guidelines.xhtml

Supplementary Table S5 and S6 show the sex-specific distribution of health-related characteristics in OPAL and ELSA cohort studies across four age groups. Overall, health-related characteristics of the OPAL participants were broadly comparable with those recruited to the nationally representative ELSA cohort study.

. Both men and women participants in the OPAL study were less likely to smoke and had a lower prevalence of self-reported heart problems, stroke and dementia.

Characteristics of included general practices

General practice area deprivation and the estimated proportion of ethnic groups registered in the practice population are described in Supplementary Table S3. Of the 35 general practices included in the study, 32 had data available on PHE national general practice profiles website. Nine of 32 practices (28.1%) were classified among the most deprived practices (IMD deciles 1-3), 14/32 (43.8%) in the most affluent practices (IMD deciles 8-10) and the remainder categorised as moderate (n=9/32; 28.1%; IMD deciles 4-7).

Over 14.3% (n=5/35) of general practices are located in rural areas, a slightly lower proportion than across rural areas in England as a whole (17.0%; n=5,598/32,844 LSOAs).

Cohort multiple randomized controlled trial

The first registered RCT utilizing the OPAL cohort study is now being undertaken. This NIHR funded trial is testing the effectiveness of a physiotherapist delivered combined physical and psychological intervention for older adults with neurogenic claudication compared to best practice advice (BOOST)[51]. The trial is registered with the International Standard Randomised Controlled Trials database, reference number ISRCTN12698674.

For peer review only - http://bmjopen.bmj.com/site/about/guidelines.xhtml

Strengths and limitations

The original target for recruitment of the OPAL cohort study was a minimum of 4,000 older adults from 32 general practices. However, uptake was better than predicted and we have recruited 5,409 older adults from 35 general practices within nine distinct areas, providing good geographical coverage within England. The wide range of self-report health measures will allow us to account for a large range of potential mediating and confounding variables.

One important limitation of the cohort is the reliance upon self-reported data. We acknowledge that performance tests may provide more reliable objective data, however, we were interested in patient reported factors and outcomes as these are feasible to capture during a patient consultation and findings may application within clinical practice. We also have obtained written informed consent to access NHS Digital and primary health care data for the majority of the participants, to allow independent verification of diagnoses related to hospital admission and attendance, and as well as important elements of health service resource use and mortality. Biological markers are not systematically collected in electronic health records and this may be a potential weaknesses. However, the OPAL cohort study was designed to elucidate the epidemiology of musculoskeletal pain and the contribution of pain on health related outcomes rather than attempt to investigate the biological underpinning of musculoskeletal pain.

Individuals living in more deprived neighbourhoods (based on practice populationdeprivation) and non-white ethnicity groups were less likely to participate in OPAL (Supplementary Table S3). This finding is consistent with other epidemiological studies which report that populations with a lower socioeconomic position are less likely to take part in research compared to those with higher socioeconomic position[52]. Nevertheless, our population is broadly representative of the English population.

For peer review only - http://bmjopen.bmj.com/site/about/guidelines.xhtml

Our findings will apply to community-dwelling older adults in England and may under represent those living in the community with severe cognitive impairment.

In terms of the representativeness of the OPAL study, demographic and health-related characteristics of OPAL participants are similar to those in the general population (2011 Census) and ELSA study (Supplementary Table S4, S5 and S6), respectively. The selected variables for the comparison analysis had good comparability in both OPAL and ELSA studies, but there were some differences. For example, in ELSA, weight was calculated using measured weight, whereas in OPAL weight was self-reported. Self-reported weight tends to be underreported, particularly by women and those who are heaviest[53]. In addition, in ELSA, the definition of 'smoker status' and health conditions combines information from previous waves, whereas in OPAL study, only baseline information was used. This may have led to a slight underestimation of the difference between ELSA and OPAL in the percentage of 'ex-smoker' and individuals with the health condition.

Future work

Data collection for the Year 1 follow-up questionnaire was completed in September 2019 and Year 2 and 3 follow-up will be completed in 2020 and 2021, respectively. We plan to administer questionnaires at annual intervals, and aim to continue this for a minimum of five years.

The potential of this data set has yet to be exploited and further work is in progress. We will start focusing on particular health domains (such as low back pain and mobility problems), together with an exploration of factors underlying the variability of those health domains. For example, we will investigate whether social, physical and psychological factors mediate the effect between low back pain and immobility. Future work will include the development of a prognostic tool to identify older adults at risk of mobility decline to help individuals, GPs and other health professionals identify risk factors and when these should be prioritised as a treatment target. This longitudinal cohort study

For peer review only - http://bmjopen.bmj.com/site/about/guidelines.xhtml

will also identify health trajectories and will examine their associations with demographic, clinical, and social factors, with the aim of identifying factors that maintain good health and independence in older people.

Collaboration

We welcome potential collaborations with other research groups. Interested researchers should contact Professor Sarah (Sallie) Lamb (S.E.Lamb@exeter.ac.uk / sarah.lamb@ndorms.ox.ac.uk) to discuss collaboration. Further information on the OPAL cohort study can be found on our website: https://www.ndorms.ox.ac.uk/rrio/opal.

For peer review only - http://bmjopen.bmj.com/site/about/guidelines.xhtml

Data sharing statement

Further information on the OPAL cohort study can be found on our website: https://www.ndorms.ox.ac.uk/rrio/opal. Unpublished data will be available for data sharing. Enquires can be made to Professor Sarah (Sallie) Lamb (Principal Investigator, e-mail: sarah.lamb@ndorms.ox.ac.uk / S.E.Lamb@exeter.ac.uk).

OPAL Study Team

Conway O, Darton F, Dutton S, Garrett A, Hagan D, Haywood D, Hewitt A, Lamb S, Marian I, Morris A, Nevay L, Nicolson P, Sanchez-Santos MT, Bruce J, Slark M, Vadher K, Ward L, Watson M, Williamson E, Arden N, Barker K, Collins G, Fairbank J, Fitch J, French D, Griffiths F, Hanson Z, Hutchinson C, Mallen C, Petrou S.

OPAL General Practice Team

Grange Hill Surgery, Birmingham; Gosford Hill Medical Centre, Oxford; River Brook Medical Centre, Birmingham; The Key Medical Practice, Oxford; Summertown Health Centre, Oxford; Alconbury and Brampton Surgeries, Cambridgeshire; Old Exchange Surgery, Cambridgeshire; The Wand Medical Centre, Birmingham; Kingsfield Medical Centre, Birmingham; Buckden and Little Paxton Surgery, Cambridgeshire; Temple Cowley Medical Group, Oxford; Keynell Covert Surgery, Birmingham; Burbury Medical Centre, Birmingham; Hollow Way Medical Centre, Oxford; Priory View Medical Centre, Leeds; Newton Surgery, Leeds; Priory Fields Surgery, Cambridgeshire, Cromwell Place Surgery, Cambridgeshire; Craven Road Medical Centre, Leeds; Queslett Medical Practice, Birmingham; Hall Street Medical Centre, Saint Helens; Vauxhall Health Centre, Liverpool; Ireland Wood Surgery, Leeds; Civic Medical Centre, Wirral; Brownlow Group Practice, Liverpool; Wareham Surgery, Dorset; The Adam Practice, Poole; The Harvey Practice, Dorset; Three Chequers Medical

For peer review only - http://bmjopen.bmj.com/site/about/guidelines.xhtml

Practice, Salisbury; Gate Medical Centre, Birmingham; Rendcomb Surgery, Cirencester; Cotswold Medical Practice, Cheltenham; Brigstock and South Norwood Partnership, Croydon; Portland Practice, Gloucestershire; Eversley Medical Centre, Croydon.

Supporting NIHR Clinical Research Networks (CRN)

Thames Valley and South Midlands, Eastern; Yorkshire and the Humber, North West Coast; Wessex, West of England; West Midlands, South London.

Competing interests

All authors have completed the Unified Competing Interest form at www.icmje.org/coi_disclosure.pdf. JB reports reports grant funding from NIHR, Diabetes Research UK and Medronic Ltd. CM reports grants from NIHR, during the conduct of the study. SL reports and declared competing interests of authors: Sarah E Lamb was on the Health Technology Assessment (HTA) Additional Capacity Funding Board, HTA End of Life Care and Add-on Studies Board, HTA Prioritisation Group Board and the HTA Trauma Board. All other authors declare no conflicts of interest.

Funding

This research is funded by the NIHR Programme Grants for Applied Research (reference: PTC-RP-PG-0213-20002). Preparatory work for the programme of research was supported by the NIHR Collaborations for Leadership in Applied Health Research and Care (CLAHRC). Christian D. Mallen. is funded by the National Institute for Health Research (NIHR) Applied Research Collaboration (West Midlands), the NIHR School for Primary Care Research and an NIHR Research Professorship in General

For peer review only

Practice (NIHR-RP-2014-04-026). Julie Bruce is supported from NIHR Research Capability Funding via University Hospitals Coventry and Warwickshire.

Disclosure

The views expressed in this article are those of the authors and not necessarily those of the NHS, the NIHR, our funding bodies or the Department of Health and Social Care.

Author contributions

MTSS participated in the data preparation, analysis, and interpretation; and the development and writing of the paper. EW participated in the OPAL study design, data collection and interpretation of the results of the paper. JB, LW and CM participated in the OPAL study design, data collection and interpretation of findings. AG and AM participated in the design of the OPAL study, data collection, data preparation and interpretation of findings. SL conceived the study, secured funding, and oversaw all aspects as principal investigator. SL participated in the design and execution of the OPAL study, and the development and writing of the paper. All authors contributed and approved the final manuscript.

Acknowledgments

The authors would like to thank the individuals who participated in The Oxford Pain, Activity and Lifestyle (OPAL) cohort study, the general practitioners and their staff for assisting with the identification and invitation of eligible individuals and the NIHR Biomedical Research Centre, Oxford for funding this study (grant reference PTC-RP-PG-0213-20002).

For peer review only - http://bmjopen.bmj.com/site/about/guidelines.xhtml

References

1. ONS. National population projections: 2016-based: Office for National Statistics; 2017 [Available from: https://www.ons.gov.uk/releases/nationalpopulationprojections2016basedstatisticalbulletin accessed 11/06/2019 2019.
2. Fried LP, Tangen CM, Walston J, et al. Frailty in older adults: evidence for a phenotype. *The journals of gerontology Series A, Biological sciences and medical sciences* 2001;56(3):M146-56. [published Online First: 2001/03/17]
3. Troutman-Jordan M, Staples J. Successful aging from the viewpoint of older adults. *Research and theory for nursing practice* 2014;28(1):87-104. [published Online First: 2014/04/30]
4. Parsons S, Gale CR, Kuh D, et al. Physical capability and the advantages and disadvantages of ageing: Perceptions of older age by men and women in two British cohorts. *Ageing and Society* 2014;34(3):452-71. doi: https://doi.org/10.1017/S0144686X12001067
5. Hardy SE, Kang Y, Studenski SA, et al. Ability to walk 1/4 mile predicts subsequent disability, mortality, and health care costs. *Journal of general internal medicine* 2011;26(2):130-5. doi: 10.1007/s11606-010-1543-2 [published Online First: 2010/10/26]
6. Ward RE, Beauchamp MK, Latham NK, et al. A Novel Approach to Identifying Trajectories of Mobility Change in Older Adults. *PloS one* 2016;11(12):e0169003. doi: 10.1371/journal.pone.0169003 [published Online First: 2016/12/23]
7. Woolf AD, Pfleger B. Burden of major musculoskeletal conditions. *Bulletin of the World Health Organization* 2003;81(9):646-56. [published Online First: 2004/01/09]
8. Global, regional, and national disability-adjusted life-years (DALYs) for 359 diseases and injuries and healthy life expectancy (HALE) for 195 countries and territories, 1990-2017: a systematic analysis for the Global Burden of Disease Study 2017. *Lancet* 2018;392(10159):1859-922. doi: 10.1016/s0140-6736(18)32335-3 [published Online First: 2018/11/13]
9. Fayaz A, Croft P, Langford RM, et al. Prevalence of chronic pain in the UK: a systematic review and meta-analysis of population studies. *BMJ open* 2016;6(6):e010364. doi: 10.1136/bmjopen-2015-010364 [published Online First: 2016/06/22]
10. Andorsen OF, Ahmed LA, Emaus N, et al. High prevalence of chronic musculoskeletal complaints among women in a Norwegian general population: the Tromso study. *BMC research notes* 2014;7:506. doi: 10.1186/1756-0500-7-506 [published Online First: 2014/08/12]
11. Eggermont LH, Leveille SG, Shi L, et al. Pain characteristics associated with the onset of disability in older adults: the maintenance of balance, independent living, intellect, and zest in the Elderly Boston Study. *J Am Geriatr Soc* 2014;62(6):1007-16. doi: 10.1111/jgs.12848 [published Online First: 2014/05/16]
12. Leveille SG, Jones RN, Kiely DK, et al. Chronic musculoskeletal pain and the occurrence of falls in an older population. *JAMA* 2009;302(20):2214-21. doi: 10.1001/jama.2009.1738
13. Whitlock EL, Diaz-Ramirez LG, Glymour MM, et al. Association Between Persistent Pain and Memory Decline and Dementia in a Longitudinal Cohort of Elders. *JAMA internal medicine* 2017;177(8):1146-53. doi: 10.1001/jamainternmed.2017.1622 [published Online First: 2017/06/07]
14. Chen Q, Hayman LL, Shmerling RH, et al. Characteristics of chronic pain associated with sleep difficulty in older adults: the Maintenance of Balance, Independent Living, Intellect, and Zest in the Elderly (MOBILIZE) Boston study. *J Am Geriatr Soc* 2011;59(8):1385-92. doi: 10.1111/j.1532-5415.2011.03544.x [published Online First: 2011/08/03]
15. Saraiva MD, Suzuki GS, Lin SM, et al. Persistent pain is a risk factor for frailty: a systematic review and meta-analysis from prospective longitudinal studies. *Age and ageing* 2018;47(6):785-93. doi: 10.1093/ageing/afy104 [published Online First: 2018/07/28]
16. Lee H, Hübscher M, Moseley GL, et al. How does pain lead to disability? A systematic review and meta-analysis of mediation studies in people with back and neck pain. *Pain* 2015;156(6):988-97. doi: 10.1097/j.pain.0000000000000146 [published Online First: 2015/03/12]
17. Leveille SG, Kiel DP, Jones RN, et al. The MOBILIZE Boston Study: design and methods of a prospective cohort study of novel risk factors for falls in an older population. *BMC geriatrics* 2008;8:16. doi: 10.1186/1471-2318-8-16 [published Online First: 2008/07/22]

For peer review only - http://bmjopen.bmj.com/site/about/guidelines.xhtml

18. Relton C, Torgerson D, O'Cathain A, et al. Rethinking pragmatic randomised controlled trials: introducing the "cohort multiple randomised controlled trial" design. *BMJ (Clinical research ed)* 2010;340:c1066. doi: 10.1136/bmj.c1066 [published Online First: 2010/03/23]
19. Clegg A, Relton C, Young J, et al. Improving recruitment of older people to clinical trials: use of the cohort multiple randomised controlled trial design. *Age and ageing* 2015;44(4):547-50. doi: 10.1093/ageing/afv044 [published Online First: 2015/04/11]
20. McManus RJ, Ryan R, Jones M, et al. How representative of primary care are research active practices? Cross-sectional survey. *Family practice* 2008;25(1):56-62. doi: 10.1093/fampra/cmm065 [published Online First: 2007/12/01]
21. Harrell Jr FE. Regression modeling strategies: with applications to linear models, logistic regression and survival analysis. New York, Springer. New York: Springer 2001.
22. Peduzzi P, Concato J, Kemper E, et al. A simulation study of the number of events per variable in logistic regression analysis. *Journal of clinical epidemiology* 1996;49(12):1373-9. doi: 10.1016/s0895-4356(96)00236-3 [published Online First: 1996/12/01]
23. Docking RE, Fleming J, Brayne C, et al. Epidemiology of back pain in older adults: prevalence and risk factors for back pain onset. *Rheumatology (Oxford)* 2011;50(9):1645-53. doi: 10.1093/rheumatology/ker175 [published Online First: 2011/05/25]
24. Bruce J, Lall R, Withers EJ, et al. A cluster randomised controlled trial of advice, exercise or multifactorial assessment to prevent falls and fractures in community-dwelling older adults: protocol for the prevention of falls injury trial (PreFIT). *BMJ open* 2016;6(1):e009362. doi: 10.1136/bmjopen-2015-009362 [published Online First: 2016/01/20]
25. Kuorinka I, Jonsson B, Kilbom A, et al. Standardised Nordic questionnaires for the analysis of musculoskeletal symptoms. *Applied ergonomics* 1987;18(3):233-7. [published Online First: 1987/09/01]
26. Parsons S, Breen A, Foster NE, et al. Prevalence and comparative troublesomeness by age of musculoskeletal pain in different body locations. *Family practice* 2007;24(4):308-16. doi: 10.1093/fampra/cmm027 [published Online First: 2007/07/03]
27. Deyo RA, Battie M, Beurskens AJ, et al. Outcome measures for low back pain research. A proposal for standardized use. *Spine (Phila Pa 1976)* 1998;23(18):2003-13. doi: 10.1097/00007632-199809150-00018 [published Online First: 1998/10/21]
28. Lamb SE, Lall R, Hansen Z, et al. A multicentred randomised controlled trial of a primary care-based cognitive behavioural programme for low back pain. The Back Skills Training (BeST) trial. *Health Technol Assess* 2010;14(41):1-253, iii-iv. doi: 10.3310/hta14410
29. de Schepper EI, Overdevest GM, Suri P, et al. Diagnosis of Lumbar Spinal Stenosis: An Updated Systematic Review of the Accuracy of Diagnostic Tests. *Spine (Phila Pa 1976)* 2013;38(8):E469-E81. doi: 10.1097/BRS.0b013e31828935ac
30. Suri P, Rainville J, Kalichman L, et al. Does this older adult with lower extremity pain have the clinical syndrome of lumbar spinal stenosis? *JAMA* 2010;304(23):2628-36. doi: 10.1001/jama.2010.1833
31. Newell AM, VanSwearingen JM, Hile E, et al. The modified Gait Efficacy Scale: establishing the psychometric properties in older adults. *Physical therapy* 2012;92(2):318-28. doi: 10.2522/ptj.20110053 [published Online First: 2011/11/15]
32. Peel C, Sawyer Baker P, Roth DL, et al. Assessing mobility in older adults: the UAB Study of Aging Life-Space Assessment. *Physical therapy* 2005;85(10):1008-119. [published Online First: 2005/09/27]
33. Lamb SE, Jørstad-Stein EC, Hauer K, et al. Development of a common outcome data set for fall injury prevention trials: the Prevention of Falls Network Europe consensus. *J Am Geriatr Soc* 2005;53(9):1618-22. doi: 10.1111/j.1532-5415.2005.53455.x
34. Gobbens RJ, van Assen MA, Luijkx KG, et al. The Tilburg Frailty Indicator: psychometric properties. *Journal of the American Medical Directors Association* 2010;11(5):344-55. doi: 10.1016/j.jamda.2009.11.003
35. Gobbens RJ, van Assen MA, Luijkx KG, et al. Determinants of frailty. *Journal of the American Medical Directors Association* 2010;11(5):356-64. doi: 10.1016/j.jamda.2009.11.008 [published Online First: 2010/06/01]

For peer review only - http://bmjopen.bmj.com/site/about/guidelines.xhtml

36. Shua-Haim J, Koppuzha G, Gross J. A simple scoring system for clock drawing in patients with Alzheimer's disease. *J Am Geriatr Soc* 1996;44(3):335. [published Online First: 1996/03/01]
37. Herdman M, Gudex C, Lloyd A, et al. Development and preliminary testing of the new five-level version of EQ-5D (EQ-5D-5L). *Qual Life Res* 2011;20(10):1727-36. doi: 10.1007/s11136-011-9903-x [published Online First: 2011/04/12]
38. Janssen MF, Pickard AS, Golicki D, et al. Measurement properties of the EQ-5D-5L compared to the EQ-5D-3L across eight patient groups: a multi-country study. *Qual Life Res* 2013;22(7):1717-27. doi: 10.1007/s11136-012-0322-4 [published Online First: 2012/11/28]
39. van Hout B, Janssen MF, Feng YS, et al. Interim scoring for the EQ-5D-5L: mapping the EQ-5D-5L to EQ-5D-3L value sets. *Value in health : the journal of the International Society for Pharmacoeconomics and Outcomes Research* 2012;15(5):708-15. doi: 10.1016/j.jval.2012.02.008 [published Online First: 2012/08/08]
40. EuroQol Group. EQ-5D 5L | Valuation: Standard value sets [Available from: https://euroqol.org/eq-5d-instruments/eq-5d-5l-about/valuation-standard-value-sets/crosswalk-index-value-calculator/ accessed 17 December 2019.
41. Public Health England National general practice profiles [Available from: http://fingertips.phe.org.uk/profile/general-practice/data accessed August 2019.
42. Department for Communities and Local Government. The English Indices of Deprivation 2015. *In London: Department for Communities and Local Government* 2015
43. Bibby P, Brindley P. Urban and rural area definitions for policy purposes in England and Wales: Methodology. *Government Statistical Service, London* 2013
44. Office for National Statistics. Open Geography Portal. 2016 [Available from: http://geoportal.statistics.gov.uk/ accessed 15 June 2020
45. Digital N. Hospital Episode Statistics (HES) [11/06/2019]. Available from: https://digital.nhs.uk/data-and-information/data-tools-and-services/data-services/hospital-episode-statistics.
46. Office for National Statistics. Census 2011 [Available from: www.nomisweb.co.uk/census/2011 accessed June 9, 2020.
47. Clemens S, Phelps A, Oldfield Z, et al. English Longitudinal Study of Ageing: Waves 0-8, 1998-2017 2019 [30th Edition:[
48. Steptoe A, Breeze E, Banks J, et al. Cohort profile: the English longitudinal study of ageing. *International journal of epidemiology* 2013;42(6):1640-8. doi: 10.1093/ije/dys168 [published Online First: 2012/11/13]
49. Abell J, Amin-Smith N, Banks J, et al. The dynamics of ageing: evidence from the English Longitudinal Study of Ageing 2002-2016 (Wave 8): The Institute for Fiscal Studies 2018.
50. Janssen B, Szende A. Population Norms for the EQ-5D. In: Szende A, Janssen B, Cabases J, eds. Self-Reported Population Health: An International Perspective based on EQ-5D. Dordrecht (NL): Springer Copyright 2014, The Author(s). 2014:19-30.
51. Williamson E, Ward L, Vadher K, et al. Better Outcomes for Older people with Spinal Trouble (BOOST) Trial: a randomised controlled trial of a combined physical and psychological intervention for older adults with neurogenic claudication, a protocol. *BMJ open* 2018;8(10):e022205. doi: 10.1136/bmjopen-2018-022205 [published Online First: 2018/10/21]
52. Galea S, Tracy M. Participation rates in epidemiologic studies. *Annals of epidemiology* 2007;17(9):643-53. doi: 10.1016/j.annepidem.2007.03.013 [published Online First: 2007/06/08]
53. Spencer EA, Appleby PN, Davey GK, et al. Validity of self-reported height and weight in 4808 EPIC-Oxford participants. *Public health nutrition* 2002;5(4):561-5. doi: 10.1079/phn2001322 [published Online First: 2002/08/21]
54. Banks J, Nazroo J, Steptoe A, editors. *Evidence from the English Longitudinal Study of Ageing 2002-2012 (Wave 6)*. London: The Institute for Fiscal Studies, 2014.
55. Banks J, Breeze E, Lessof C, et al. Retirement, health and relationships of the older population in England: The 2004 English Longitudinal Study of Ageing. *London: Institute for Fiscal Studies* 2006

For peer review only - http://bmjopen.bmj.com/site/about/guidelines.xhtml

56. Mottram S, Peat G, Thomas E, et al. Patterns of pain and mobility limitation in older people: cross-sectional findings from a population survey of 18,497 adults aged 50 years and over. *Qual Life Res* 2008;17(4):529-39. doi: 10.1007/s11136-008-9324-7
57. Driscoll T, Jacklyn G, Orchard J, et al. The global burden of occupationally related low back pain: estimates from the Global Burden of Disease 2010 study. *Annals of the rheumatic diseases* 2014;73(6):975-81. doi: 10.1136/annrheumdis-2013-204631 [published Online First: 2014/03/26]
58. Guralnik JM, Fried LP, Simonsick EM, et al. The Women's Health and Aging Study: Health and Social Characteristics of Older Women with Disability. Bethesda, MD: National Institute of Aging, 1995.
59. Shuval K, Kohl HW, 3rd, Bernstein I, et al. Sedentary behaviour and physical inactivity assessment in primary care: the Rapid Assessment Disuse Index (RADI) study. *British journal of sports medicine* 2014;48(3):250-5. doi: 10.1136/bjsports-2013-092901 [published Online First: 2013/10/23]
60. Rose SB, Elley CR, Lawton BA, et al. A single question reliably identifies physically inactive women in primary care. *The New Zealand medical journal* 2008;121(1268):U2897. [published Online First: 2008/02/08]
61. Buysse DJ, Reynolds CF, 3rd, Monk TH, et al. The Pittsburgh Sleep Quality Index: a new instrument for psychiatric practice and research. *Psychiatry research* 1989;28(2):193-213. [published Online First: 1989/05/01]
62. Collin C, Wade DT, Davies S, et al. The Barthel ADL Index: a reliability study. *International disability studies* 1988;10(2):61-3. [published Online First: 1988/01/01]
63. Gompertz P, Pound, P., & Ebrahim, S. A postal version of the Barthel Index. *Clinical Rehabilitation*, *8*(3), 233-239. *Clinical Rehabilitation* 1994;8(3):233-39.
64. Von Korff M, Ormel J, Keefe F, et al. Grading the severity of chronic pain. *Pain* 1992;50(2):133-49.
65. Syddall HE, Westbury LD, Cooper C, et al. Self-reported walking speed: a useful marker of physical performance among community-dwelling older people? *Journal of the American Medical Directors Association* 2015;16(4):323-8. doi: 10.1016/j.jamda.2014.11.004 [published Online First: 2014/12/20]
66. Chung J, Demiris G, Thompson HJ. Instruments to assess mobility limitation in community-dwelling older adults: a systematic review. *Journal of aging and physical activity* 2015;23(2):298-313. doi: 10.1123/japa.2013-0181 [published Online First: 2014/03/05]
67. Pinto E, Peters R. Literature review of the Clock Drawing Test as a tool for cognitive screening. *Dement Geriatr Cogn Disord* 2009;27(3):201-13. doi: 10.1159/000203344
68. Laidlaw K, Power MJ, Schmidt S. The Attitudes to Ageing Questionnaire (AAQ): development and psychometric properties. *International journal of geriatric psychiatry* 2007;22(4):367-79. doi: 10.1002/gps.1683 [published Online First: 2006/10/20]

For peer review only

For peer review only - http://bmjopen.bmj.com/site/about/guidelines.xhtml

Tables and Figures

Figure 1. Locations of the areas from which the OPAL Cohort Study was derived. Map of England divided by counties.

Figure 2. Health conditions in men and women of OPAL Cohort Study

For peer review only

Table 1. Measures included in the OPAL Cohort Study

Data collection for the OPAL Cohort Study		
**Domain measured**	**Self-reported measure**	**Years (Y)**
Socio-demographic	Age, sex, education, relationship status<br>Participation in clubs and groups[54]<br>Requires unpaid/paid carer	Y0-Y5
	Ethnicity<br>Number of live births and stillbirths	Y0
Socio-economic	Participant and GP Area deprivation obtained from postcodes [42]<br>Current work status [55]<br>Type of housing<br>Adequacy of income [56]	Y0-Y5
	Main occupation during lifetime [57] and self-rating of strenuousness of occupation<br>Internet access	Y0
Lifestyle	Weight<br>Alcohol and smoking [58]<br>Current physical activity [59]	Y0-Y5
	Height<br>Lifetime physical activity [60]	Y0
General health data	Self-reported comorbidities and medication use<br>Sleep quality - Pittsburgh Sleep Quality Index [61] and average number of hours sleep each night<br>Incontinence - 2 items from Barthel Index [62 63]<br>Falls in the last 12 months [33]<br>Broken bones or fractures in the last 12 months	Y0-Y5
Musculoskeletal symptoms	The Nordic Musculoskeletal Questionnaire adapted version [25 26]	Y0-Y5
	Report of back pain in last 6 weeks, troublesomeness, onset of back pain and nature of back pain [28]<br>Leg pain and symptoms related to low back pain<br>Screening questions for neurogenic claudication [29]	Y0-Y5
	Report of knee pain, troublesomeness, interference with daily activity [64]	Y1-Y2

For peer review only - http://bmjopen.bmj.com/site/about/guidelines.xhtml

Data collection for the OPAL Cohort Study		
**Domain measured**	**Self-reported measure**	**Years (Y)**
	Location of knee pain	Y1
Mobility	Change in mobility in the last year.<br>Self-rated walking speed [65]<br>Use of walking aids (inside and outside)<br>Mobility concerns<br>Access to transport [54]<br>Life-Space assessment [32]<br>Single item from the Modified Gait Self-Efficacy Scale (10-item) [31]	Y0-Y5
	Difficulty with balance while walking	Y2-Y5
	Difficulties walking a half of mile [66]<br>Difficulties walking up and down a flight of stairs [66]	Y3-Y5
Disability	Self-reported difficulty with Activities of Daily Living (bathing, transfers, toilet use, dressing and eating)	Y2-Y5
Frailty	Tilburg Frailty Index [34 35]	Y0-Y5
Cognition	Clock Drawing Test [67]	Y0-Y5
Beliefs about ageing	Attitude to ageing questionnaire – physical changes subscale [68]	Y0-Y5
Health related quality of life	EuroQol 5-Dimension Health Questionnaire, five-level version [37]<br>EuroQol-Visual Analog Scale (EQ-VAS) [37]	Y0-Y5

For peer review only - http://bmjopen.bmj.com/site/about/guidelines.xhtml

Table 2. Sociodemographic and life-style factors of men and women in the OPAL Cohort Study

Characteristic	Men (n=2,625)	Women (n=2,784)
Age, mean (SD)	74.8 (6.7)	75.0 (6.8)
Age groups, n (%)		
65-69	784 (29.9)	801 (28.8)
70-74	696 (26.5)	734 (26.4)
75-79	542 (20.7)	618 (22.2)
80-84	355 (13.5)	356 (12.8)
85-89	196 (7.5)	203 (7.3)
90+	52 (2.0)	72 (2.6)
Ethnicity (White), n (%)	2,465 (93.9)	2,667 (95.8)
Relationship status, n (%)		
Married/Civil Union	1,897 (72.3)	1,506 (54.1)
Living with Partner	114 (4.3)	85 (3.1)
Unmarried (never married)	117 (4.5)	105 (3.8)
Separated/Divorced	185 (7.1)	273 (9.8)
Widow/Widower	305 (11.6)	809 (29.1)
Live alone, n (%)	534 (20.3)	1,021 (36.7)
Education, n (%)		
High professional or university	1,017 (38.7)	895 (32.2)
Secondary school only	1,370 (52.2)	1,681 (60.4)
None or primary	219 (8.3)	189 (6.8)
Work status (Retired), n (%)	2,187 (83.3)	2,402 (86.3)
Quintiles of IMD, n (%)		
Q1 – Most deprived	293 (11.2)	289 (10.4)
Q2	323 (12.3)	339 (12.2)
Q3	542 (20.7)	613 (22.0)
Q4	575 (21.9)	591 (21.2)
Q5 – Least deprived	892 (34.0)	952 (34.2)
BMI (kg/m^2), mean (SD)	26.8 (4.3)	26.4 (5.3)
Smoking status, n (%)		
Never	1,071 (40.8)	1,618 (58.1)
Ex-Smoker	1,401 (53.4)	1,040 (37.4)
Current	145 (5.5)	118 (4.2)
Cigarettes per day, median (IQR)	15 (10-20)	10 (5-17)
Alcohol intake once per week, n (%)	1,861 (70.9)	1,361 (48.9)

SD=standard deviation; IMD=Index of Multiple Deprivation. Data included older adults 65 years and older at baseline 2016-2018.

For peer review only

For peer review only - http://bmjopen.bmj.com/site/about/guidelines.xhtml

Table 3. Health-related characteristics of men and women at the OPAL Cohort Study

Health-related characteristics	Men (n=2,625)	Women (n=2,784)
**Musculoskeletal disorders** in the last 6 weeks**,** n (%)		
Low back (small of the back)	1,098 (41.8)	1,306 (46.9)
One of both knees	932 (35.5)	1,132 (40.7)
Wrist/hands	653 (24.9)	1,053 (37.8)
Neck	673 (25.6)	951 (34.2)
Shoulders	667 (25.4)	948 (34.1)
One of both hips/thighs	599 (22.8)	875 (31.4)
One or both ankles/feet	559 (21.3)	755 (27.1)
Upper back	160 (6.1)	346 (12.4)
Elbows	161 (6.1)	173 (6.2)
Any pain, n (%)	2,137 (81.4)	2,406 (86.4)
**Mobility**		
Confidence to walk half a mile, median (IQR)	10 (9-10)	10 (6-10)
Outdoor walking pace, n (%)		
Fast	91 (3.5)	93 (3.3)
Fairly brisk	534 (20.3)	572 (20.6)
Normal	994 (37.9)	958 (34.4)
stroll at an easy pace	647 (24.7)	726 (26.1)
Very slow	326 (12.4)	395 (14.2)
Unable to walk	19 (0.7)	27 (1.0)
Walking rate than 1 year ago, n (%)		
Much better	52 (2.0)	84 (3.0)
Somewhat better	114 (4.3)	101 (3.6)
About the same	1,822 (69.4)	1,831 (65.8)
Somewhat worse	507 (19.3)	622 (22.3)
Much worse	113 (4.3)	133 (4.8)
Walking aid use inside (Yes), n (%)	108 (4.1)	153 (5.5)
Walking aid use outside (Yes), n (%)	306 (11.7)	435 (15.6)
**Falls** in the last year, n (%)		
None	1,900 (72.4)	1,906 (68.5)
One fall	474 (18.1)	624 (22.4)
More than one fall	235 (9.0)	236 (8.5)
**Frailty,** Tilburg frailty score, median (IQR)	2 (1-4)	3 (1-5)
**Clock-drawing test**, n (%)		
1 point	9 (0.3)	5 (0.2)
2 points	28 (1.1)	45 (1.6)
3 points	112 (4.3)	102 (3.7)
4 points	210 (8.0)	273 (9.8)
5 points	445 (17.0)	487 (17.5)
6 points	1,756 (66.9)	1,793 (64.4)
**Quality of life**		
EQ-5D crosswalk index value, mean (SD)	0.79 (0.19)	0.76 (0.21)
EQ-VAS, mean (SD)	79.1 (16.7)	77.7 (18.0)

For peer review only

For peer review only - http://bmjopen.bmj.com/site/about/guidelines.xhtml

Sample sizes may vary due to missing values; data included older adults 65 years and older at baseline 2016-2018.

For peer review only

For peer review only - http://bmjopen.bmj.com/site/about/guidelines.xhtml

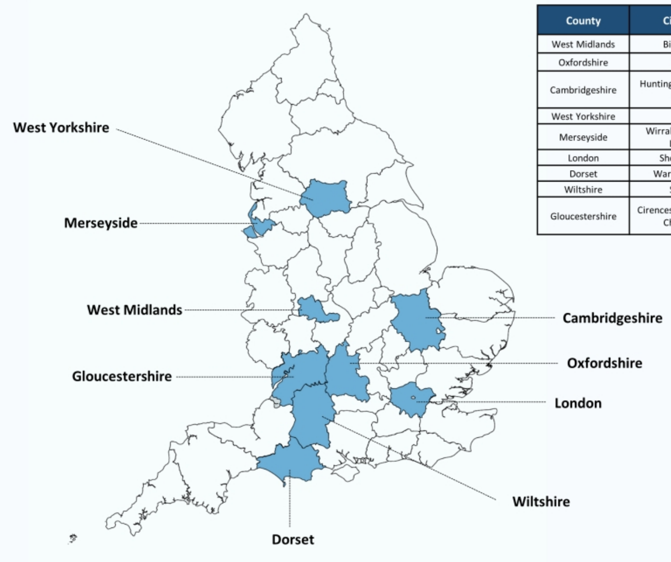

County	City/Town	General practices	No. Invited	No. Participants
West Midlands	Birmingham	8	2,623	754
Oxfordshire	Oxford	5	1,844	905
Cambridgeshire	Huntingdon, Saint Ives, Buckden	5	1,891	1,075
West Yorkshire	Leeds	4	1,782	665
Merseyside	Wirral, Saint Helens, Liverpool	4	1,502	501
London	Shout London	2	652	164
Dorset	Wareham, Poole	3	1,098	547
Wiltshire	Salisbury	1	386	214
Gloucestershire	Cirencester, Northleach, Cheltenham	3	1,061	584
		**35**	**12,839**	**5,409**

Locations of the areas from which the OPAL Cohort Study was derived. Map of England divided by counties

270x160mm (300 x 300 DPI)

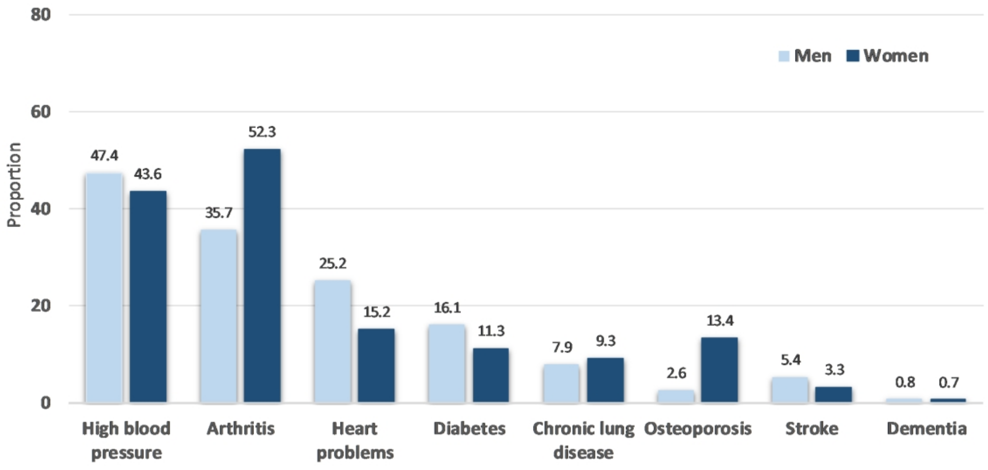

Health conditions in men and women of OPAL Cohort Study

196x101mm (300 x 300 DPI)

Supplementary Data

Supplemental Figure S1. Flow chart of baseline participants in the OPAL cohort study

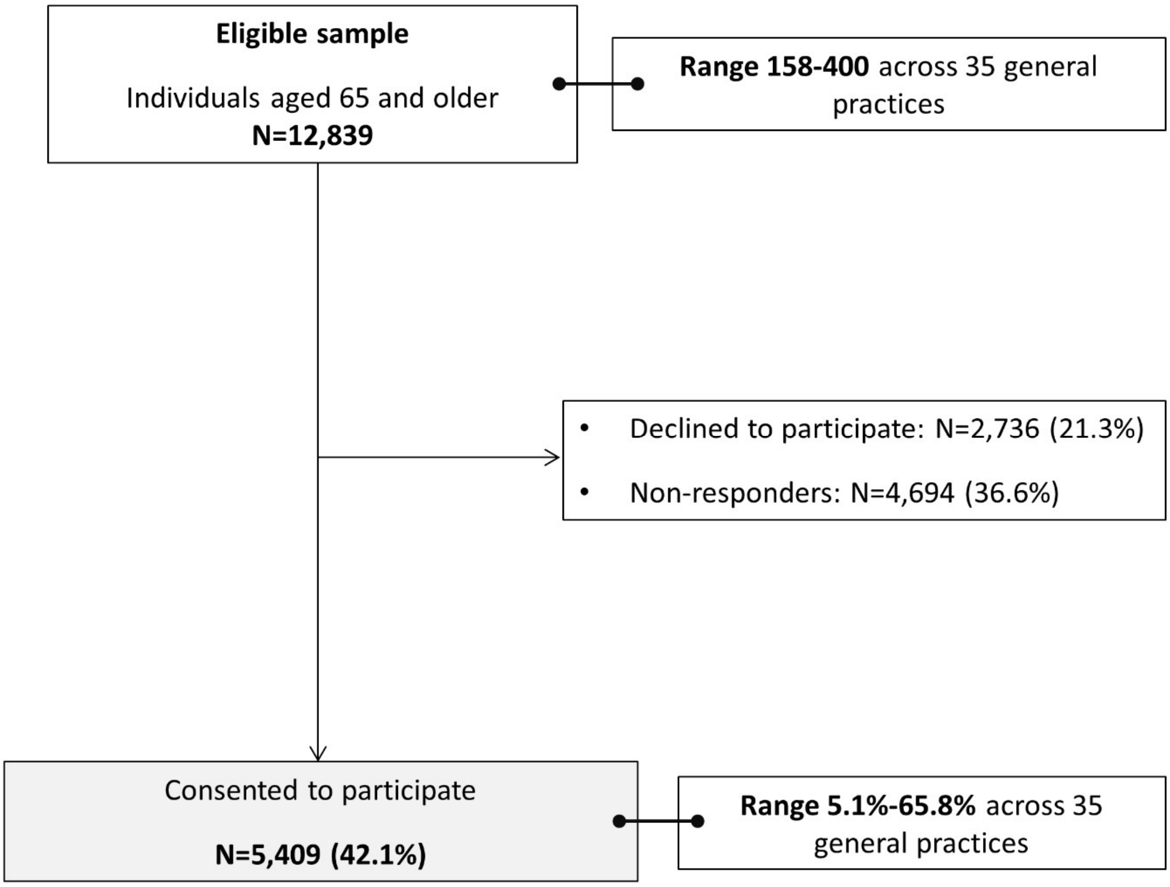

For peer review only - http://bmjopen.bmj.com/site/about/guidelines.xhtml

Supplemental Table S1. Variables used in the OPAL and the ELSA cohort studies.

Variable	Question(s), answer(s) posed to OPAL study	Question(s), answer(s) posed to ELSA study	Name (label) of the variable used for the comparison study
age	Date of birth and date of completion of questionnaire	Age in 5 year bands<br>• 65-69<br>• 70-74<br>• 75-79<br>• 80-84<br>• 85+	'ageg5' (Age variable in 5 year bands) (Derived variable from Institute for fiscal studies (IFS))
Sex	Gender: Male and Female	Sex: Male and Female	'indsex' (Sex variable)
Work status	Which of the following best describes your CURRENT work status?<br>• Retired	Which of the following best describes your CURRENT work status?<br>• Retired	'wpdes' (Best description of current situation)
Relationship status	What is your current relationship status?<br>• Married/Civil Union	What is your current legal marital status?<br>• Married/Civil partner	'dimarr' (Marital status - combined marriage/civil partnership)
Weight	What is your weight?<br>• In Kilograms	Weight measurement<br>• In Kilograms<br>Note: Participants with weight of 37 kg or lower were excluded from the analysis of this variable (n=282) due to the lowest cut-off used in the OPAL cohort study	'estwt' (Final measured or estimated weight (kg))
Smoking status	Which of the following describes your current cigarette smoking status?<br>• Never<br>• Ex-smoker<br>• Current smoker	Smoker status (past or present):<br>• Never<br>• Ex-smoker<br>• Current smoker	'smokerstat' (Derived variable from IFS (non-financial))
Chronic health conditions	*Has your doctor or nurse ever told you that you have any of the following conditions?*	*Our records show that in the last interview you said that you had been told by a doctor that you had any of the following conditions.*<br><br>*Do you still have the condition?*	*Diagnosed last interview AND confirms previous chronic condition*<br><br>*OR*<br><br>*Chronic condition since last interview*

For peer review only

For peer review only - http://bmjopen.bmj.com/site/about/guidelines.xhtml

		Since last interview, has a doctor ever told you that you have any of the conditions on this card?	
Heart problems	• Angina or heart troubles	• Angina<br>• A heart attack (including myocardial infarction or coronary thrombosis)<br>• Congestive heart failure<br>• A heart murmur<br>• An abnormal heart rhythm<br>• Any other heart trouble	*Angina*: 'hedawan', 'hedacan', 'hediman'<br>*Heart attach*: 'hedawmi', 'hedacmi', 'hedimmi'<br>Congestion heart failure: 'hedawhf', 'hedachf', 'hedimhf'<br>*Heart murmur*: 'hedawhm', 'hedachm', 'hedimhm'<br>*Abnormal heart rhythm*: 'hedawar', 'hedacar', 'hedimar'<br>*Other*: 'hedaw95', 'hedac95', 'hedia95'
Diabetes	• Diabetes (Types I or II)	• Diabetes or high blood sugar	'hedawdi', 'hedacdi', 'hedimdi'
High blood pressure	• High blood pressure	• High blood pressure or hypertension	'hedawbp', 'hedacbp', 'hedimbp'
Stroke	• Stroke	• A stroke (cerebral vascular disease)	'hedawst', 'hedacst', 'hedimst'
Arthritis	• Arthritis	• Arthritis (including osteoarthritis, or rheumatism)	'hedbwar', 'hedbdar', 'hedibar'
Dementia	• Dementia	• Dementia, senility, or any other serious memory impairment	'hedbwde', 'hedbdde', 'hedibde'
Osteoporosis	• Osteoporosis	• Osteoporosis, sometimes called thin or brittle bones	'hedbwos', 'hedbdos', 'hedibos'
Chronic lung disease	• Chronic lung disease or Asthma	• Chronic lung disease such as chronic bronchitis or emphysema<br>• Asthma	*Chronic lung disease*: 'hedbwlu', 'hedbdlu', 'hediblu'<br><br>*Asthma*: 'hedbwas', 'hedbdas', 'hedibas'

For peer review only - http://bmjopen.bmj.com/site/about/guidelines.xhtml

Variable	Question, answer(s) posed to OPAL study	Question, answer(s) posed to England 2011 Census	Variable used for the comparison study
Ethnicity	To which of these ethnic groups do you consider you belong?<br>• Non-white (Mixed, Indian, Pakistani, Bangladeshi, Black/Black British, Chinese and other ethnic group)	What is your ethnic group?<br>• Non-white (Mixed/multiple ethnic groups, Asian/Asian British, Black/African/Caribbean/Black British and other ethnic group)	Ethnicity - divided into white and non-white

For peer review only

For peer review only - http://bmjopen.bmj.com/site/about/guidelines.xhtml

Supplemental Table S2. Characteristics of OPAL participants and non-participants

Characteristic	Eligible (N=12,839)	Responders		Non-Responders (N=4,694)	Non-participants (N=7,430)
		Consented (N=5,409)	Declined (N=2,736)		
Age*, mean (SD)	75.5 (7.2)	74.9 (6.8)	77.0 (7.4)	75.4 (7.4)	75.9 (7.4)
Age* groups, n (%)					
65-69	3,611 (28.1)	1,601 (29.6)	598 (21.9)	1,412 (30.1)	2,010 (27.1)
70-74	3,124 (24.3)	1,426 (26.4)	598 (21.9)	1,100 (23.4)	1,698 (22.9)
75-79	2,690 (21.0)	1,155 (21.4)	615 (22.5)	920 (19.6)	1,535 (20.7)
80+	3,414 (26.6)	1,227 (22.7)	925 (33.8)	1,262 (26.9)	2,187 (29.4)
Sex, n (%)*					
Male	5,943 (47.7)	2,625 (48.5)	1,159 (43.7)	2,226 (49.0)	3,385 (47.1)
Female	6,506 (52.3)	2,784 (51.5)	1,492 (56.3)	2,313 (51.0)	3,805 (52.9)

We did not have sex available for one site, so it was excluded for the analysis (N=390).
*The age of eligible individuals was calculated based on date when the questionnaire was sent and date of birth.

For peer review only

Supplemental Figure S2. Age distribution between participants (dark blue) and non-participants (pink) in the OPAL cohort study by general practice.

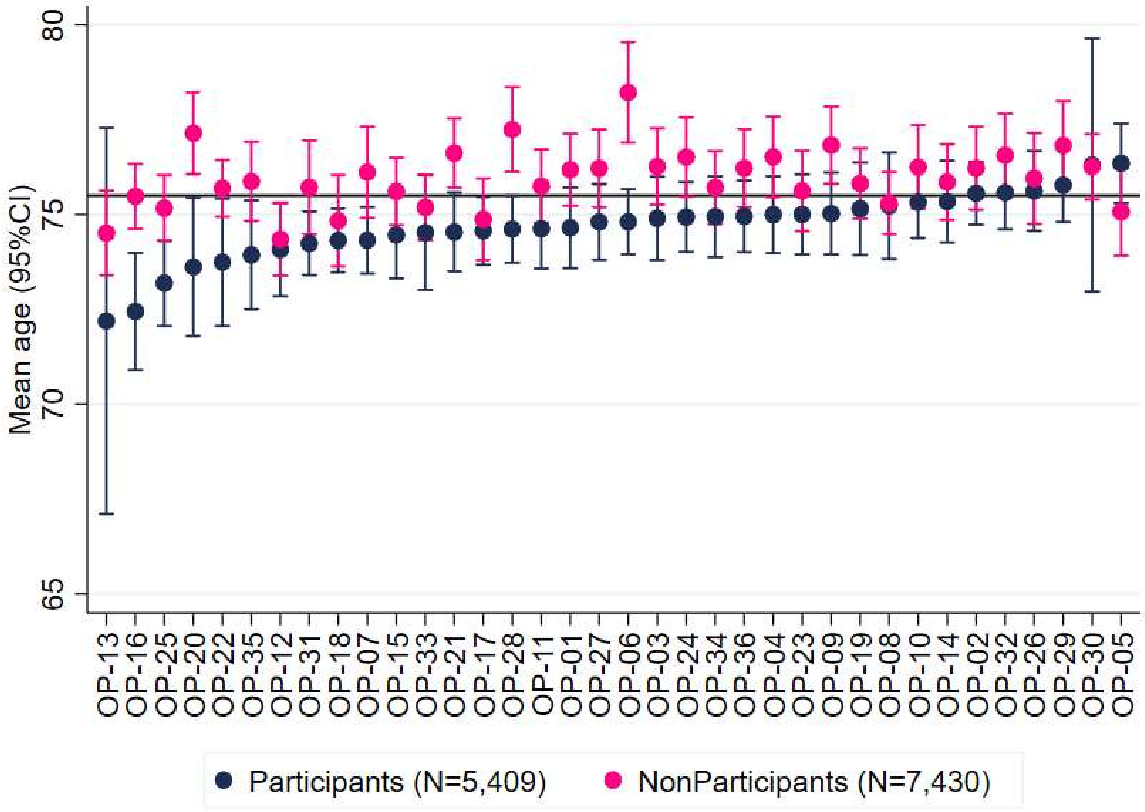

For peer review only - http://bmjopen.bmj.com/site/about/guidelines.xhtml

Supplemental Figure S3. Sex distribution between participants (dark blue) and non-participants (pink) in the OPAL cohort study by general practice.

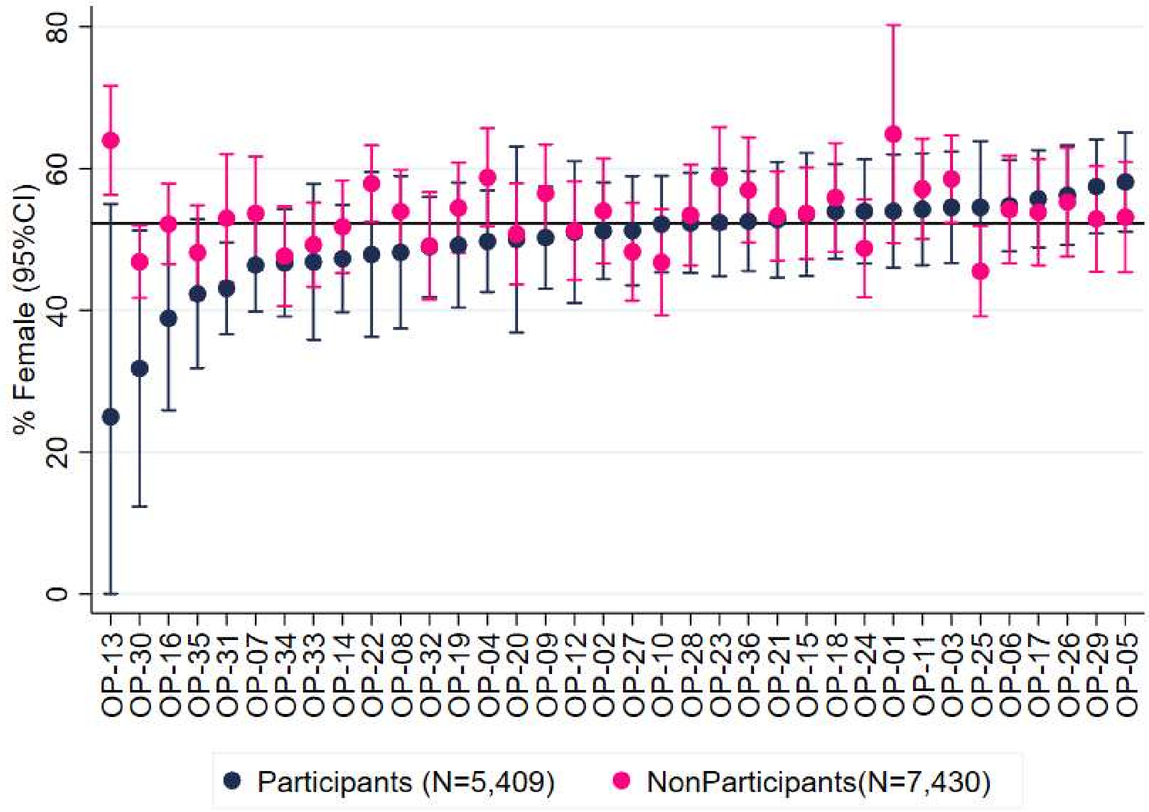

Supplemental Table S3. Area deprivation and ethnicity based on each general practice.

General practice	Eligible individuals	%Response rate	Practice IMD 2015 decile (1 More deprived to 10 Least deprived)	Estimated proportion of non-white ethnic groups in practice population		
				%Mixed	%Asian	%Black
OP-01	390	38.5%	-	-	-	-
OP-02	381	54.3%	10	2.3	5.8	1.8
OP-03	400	38.5%	4	4.4	12.8	4.1
OP-04	381	49.1%	10	2.1	5.0	1.7
OP-05	349	54.7%	10	3.4	9.1	1.8
OP-06	396	58.1%	10	1.7	2.1	1.3
OP-07	371	59.8%	10	1.4	3.6	0.0
OP-08	361	23.0%	1	6.9	36.8	21.3
OP-09	385	48.1%	5	4.6	15.7	4.0
OP-10	378	54.8%	10	1.1	1.1	0.0
OP-11	342	44.7%	6	4.3	12.7	6.8
OP-12	295	32.5%	2	4.7	6.1	5.0
OP-13	158	5.1%	1	3.9	62.0	19.1
OP-14	391	42.7%	6	4.4	13.9	6.5
OP-15	356	35.7%	2	2.5	5.9	2.7
OP-16	351	15.4%	1	6.1	31.4	16.7
OP-17	370	54.3%	7	2.2	5.3	2.0
OP-18	376	57.2%	10	1.4	3.7	0.0
OP-19	359	34.5%	5	4.4	10.9	6.3
OP-20	245	22.9%	6	2.2	7.4	3.4
OP-21	386	37.3%	3	0.0	1.1	0.0
OP-22	394	18.0%	1	1.5	2.5	1.7
OP-23/36*	350/366	47.4%/53.0%	8	2.0	3.2	0.0
OP-24	377	46.7%	7	0.0	1.2	0.0
OP-25	345	31.9%	3	3.9	15.2	4.4
OP-26	353	54.4%	8	0.0	0.0	0.0
OP-27	363	44.6%	-	-	-	-
OP-28	382	50.5%	10	0.0	1.2	0.0
OP-29	396	55.4%	9	1.0	1.8	0.0
OP-30	389	5.7%	-	-	-	-
OP-31	342	65.8%	8	0.0	1.1	0.0
OP-32	359	53.5%	9	0.0	0.0	0.0
OP-33	351	22.5%	4	7.2	26.9	33.8
OP-34	360	46.4%	8	1.4	2.8	0.0
OP-35	301	28.2%	3	7.2	29.6	28.2

IMD=Index of Multiple deprivation. 8 general practices had a response rate below (red) and 13 above (green) to the expected rate (<30% and >50%, respectively). Information found on the following government website: https://fingertips.phe.org.uk/profile/general-practice (Accessed August 2019). *Two different random samples of individuals were selected from the same general practice.

For peer review only

Supplemental Table S4. Age and ethnicity distribution in the OPAL and population estimates 2011 Census in England by sex

	OPAL (Observed %)			2011 England Census (%)		
	**Overall**	**Female**	**Male**	**Overall**	**Female**	**Male**
Age						
65-69	29.3	28.8	29.9	29.0	26.8	31.7
70-74	26.4	26.4	26.5	23.6	22.3	25.2
75-79	21.5	22.2	20.7	19.3	19.0	19.7
80 and over	22.8	22.7	23.0	28.2	31.9	23.5
Ethnicity, non-white	5.1	4.1	6.0	4.7	4.5	5.1
N	5,409	2,784 (51.5)	2,625 (48.5)	8,660,529	4,815,690 (55.6)	3,844,839 (44.4)

The 2011 England Census data were collected from: https://www.ons.gov.uk/

For peer review only

Supplemental Table S5. Characteristics of **women** in the OPAL and ELSA cohort studies by age groups

**Characteristics**	**OPAL (Observed %)**				**ELSA (Estimated % [95%CI])**			
	**65-69**	**70-74**	**75-79**	**80+**	**65-69**	**70-74**	**75-79**	**80+**
Relationship status								
Married/Civil Union	66.0	62.5	52.4	30.7	69.0 [65.8-72.1]	64.6 [60.8-68.2]	54.9 [50.4-59.2]	30.1 [26.6-33.9]
Work status, Retired	75.9	87.3	92.7	91.9	77.3 [74.2-80.0]	88.5 [85.7-90.8]	90.2 [87.3-92.6]	93.2 [91.1-94.9]
Weight (kg), mean (SD)	70.7 (14.8)	69.7 (14.7)	68.4 (13.1)	65.2 (13.0)	73.9 [72.8-75.1]	72.8 [71.5-74.1]	70.3 [69.0-71.6]	66.2 [65.0-67.4]
Smoking status,								
Ex-Smoker	38.3	40.2	35.3	34.9	46.7 [43.3-50.2]	56.8 [52.9-60.7]	49.8 [45.3-54.2]	53.5 [49.5-57.4]
Current	5.2	4.8	5.0	1.6	11.6 [9.5-14.1]	9.1 [7.0-11.8]	7.1 [4.9-10.1]	3.9 [2.6-5.9]
Health conditions,								
Heart problems	8.6	12.4	15.7	27.7	18.5 [16.0-21.4]	21.2 [18.1-24.6]	25.7 [22.0-29.7]	34.3 [30.6-38.2]
Diabetes	8.7	11.3	11.5	14.3	11.4 [9.3-13.8]	14.1 [11.6-17.1]	13.4 [10.6-16.7]	16.9 [14.1-20.2]
High Blood pressure	32.5	42.4	48.7	54.4	36.5 [33.2-39.9]	41.3 [37.5-45.3]	50.7 [46.2-55.1]	57.6 [53.6-61.4]
Stroke	2.1	2.2	2.9	6.7	3.3 [2.3-4.8]	4.3 [2.9-6.2]	7.8 [5.7-10.6]	11.2 [9.0-13.9]
Arthritis	45.6	51.8	55.0	58.6	49.9 [46.4-53.3]	54.3 [50.4-58.2]	58.3 [53.8-62.6]	62.4 [58.4-66.1]
Dementia	0.1	0.3	0.7	1.7	0.3 [0.1-1.1]	1.5 [0.8-2.8]	1.5 [0.7-3.2]	5.7 [4.1-7.8]
Osteoporosis	9.7	11.0	15.2	19.2	13.6 [11.4-16.2]	18.1 [15.2-21.4]	16.8 [13.8-20.4]	21.2 [18.2-24.6]
Chronic lung disease	10.1	9.4	10.2	7.1	6.8 [5.3-8.8]	8.2 [6.3-10.7]	8.7 [6.4-11.7]	6.1 [4.6-8.2]
Unweighted N	801	734	618	631	888	679	534	720

ELSA=The English Longitudinal Study of Ageing, a national probability sample of non-institutionalised older people. Wave 8 (2016-2017) was used for this analysis. For variable definitions, see Supplemental Table S1 and for ELSA data management, see Stata do-file "Data_management_wave8_Dec2019.do". Data were weighted to correct for non-response in the ELSA cohort study

For peer review only

Supplemental Table S6. Characteristics of **men** in the OPAL and ELSA cohort studies by age groups

**Characteristics**	**OPAL (Observed %)**				**ELSA (Estimated % [95%CI])**			
	**65-69**	**70-74**	**75-79**	**80+**	**65-69**	**70-74**	**75-79**	**80+**
Relationship status								
Married/Civil Union	75.8	75.9	72.1	63.7	76.5 [72.9-79.8]	78.0 [74.3-81.2]	72.6 [68.1-76.7]	64.2 [59.6-68.6]
Work status, Retired	71.1	81.8	90.6	94.5	74.1 [70.5-77.5]	88.0 [85.2-90.4]	93.9 [91.3-95.8]	97.3 [95.4-98.4]
Weight (kg), mean (SD)	85.0 (15.6)	83.7 (15.4)	81.5 (13.8)	78.1 (12.6)	87.3 [86.0-88.7]	84.5 [83.1-85.8]	81.6 [80.3-83.0]	78.6 [77.3-79.8]
Smoking status,								
Ex-Smoker	49.0	54.0	55.4	56.4	61.8 [57.8-65.6]	64.0 [59.9-67.9]	66.9 [62.2-71.3]	75.0 [70.8-78.7]
Current	8.8	5.2	4.6	2.5	9.7 [7.4-12.6]	10.2 [7.7-13.2]	8.2 [5.9-11.4]	2.3 [1.3-4.0]
Health conditions,								
Heart problems	18.4	25.0	27.7	32.2	20.2 [17.1-23.7]	28.5 [24.9-32.4]	35.1 [30.6-39.8]	40.2 [35.8-44.9]
Diabetes	16.1	15.4	16.1	17.1	14.5 [11.8-17.6]	18.0 [14.9-21.5]	19.4 [15.7-23.7]	15.6 [12.5-19.2]
High Blood pressure	44.3	48.7	48.0	49.4	39.5 [35.6-43.5]	47.5 [43.4-51.6]	49.8 [45.0-54.6]	51.9 [47.2-56.5]
Stroke	2.9	4.2	6.6	8.8	5.2 [3.6-7.4]	6.6 [4.8-9.1]	8.7 [6.3-11.7]	16.8 [13.5-20.6]
Arthritis	31.1	32.6	37.5	43.5	31.6 [27.9-35.4]	37.0 [33.1-41.1]	40.6 [36.0-45.4]	41.5 [37.0-46.2]
Dementia	0.4	0.4	0.7	2.0	0.5 [0.2-1.6]	1.7 [0.8-3.3]	2.3 [1.2-4.4]	4.9 [3.3-7.3]
Osteoporosis	1.4	2.3	3.5	3.7	2.0 [1.2-3.5]	5.7 [4.0-8.1]	3.6 [2.1-5.9]	3.5 [2.1-5.7]
Chronic lung disease	7.5	7.8	7.9	8.3	7.4 [5.4-10.0]	10.2 [7.9-13.1]	11.6 [8.8-15.2]	8.1 [5.9-10.9]
Unweighted N	784	696	542	603	659	624	458	503

ELSA=The English Longitudinal Study of Ageing, a national probability sample of non-institutionalised older people. Wave 8 (2016-2017) was used for this analysis. For variable definitions, see Supplemental Table S1 and for ELSA data management, see Stata do-file “Data_management_wave8_Dec2019.do”. Data were weighted to correct for non-response in the ELSA cohort study

For peer review only - http://bmjopen.bmj.com/site/about/guidelines.xhtml

For peer review only

For peer review only - http://bmjopen.bmj.com/site/about/guidelines.xhtml

```
***************************************************************************
* University of Oxford
* ELSA
* December 2019
*****************************************************************************

*Data: wave_8_elsa_data_eul_v2.dta

*Variables needed for health comparison from Wave 8
*Identifier
* idauniq   Variable label = Unique individual serial number
* idahhw8   Variable label = Analytical wave-specific individual
* perid     Variable label = Person ID
* samptyp   Variable label = Sampling status
* w8xwgt    Variable label = Wave 8 cross-sectional weight
* w8indout  Variable label = Individual outcome code

*Demography
* Derived variables are denoted with "(D)" at the beginning of the variable label:
* indager   Variable label = (D) Definitive age variable collapsed at 90+: priority diag, dhage
* indsex    Variable label = (D) Definitive sex variable: priority disex, dhsex
* fqethnmr  Variable label = (D) Ethnicity recoded into white and non-white (consolidated)
* wpdes     Variable label = Best description of current situation (retired)
* dimarr    Variable label = (D) Respondent current legal marital status - combined marriage/civil partnership
* estwt     Variable label = (D) Weight: final measured or estimated weight (kg)

*Health-related variables
*--Heart problems
*Angina
* hedawan   Variable label = Diagnosed angina fed forward
* hedacan   Variable label = Whether confirms angina diagnosis
* hediman   Variable label = Cardiovascular disease: angina diagnosis newly reported (merged)
*Heart attack
* hedawmi   Variable label =  Diagnosed heart attack fed forward
* hedacmi   Variable label =  Whether confirms heart attack diagnosis
* hedimmi   Variable label = Cardiovascular disease: heart attack diagnosis newly reported (merged)
*Congestive heart failure
* hedawhf   Variable label =  Diagnosed congestive heart failure fed forward
* hedachf   Variable label =  Whether confirms congestive heart failure diagnosis
* hedimhf   Variable label =  Cardiovascular disease: congestive heart failure diagnosis newly reported (merged)
*Heart murmur
* hedawhm   Variable label =  Diagnosed heart murmur fed forward
* hedachm   Variable label =  Whether confirms heart murmur diagnosis
* hedimhm   Variable label =  Cardiovascular disease: heart murmur diagnosis newly reported (merged)
*Abnormal heart rhythm
* hedawar   Variable label =  Diagnosed abnormal heart rhythm fed forward
* hedacar   Variable label =  Whether confirms abnormal heart rhythm diagnosiss
* hedimar   Variable label =  Cardiovascular disease: abnormal heart rhythm diagnosis newly reported (merged)
*Other heart disease
* hedaw95   Variable label =  Diagnosed other heart disease fed forward
* hedac95   Variable label =  Whether confirms other heart disease diagnosis

*--Diabetes
*Diabetes or high blood sugar
* hedawdi   Variable label = Diagnosed diabetes or high blood sugar fed forward
* hedacdi   Variable label = Whether confirms diabetes or high blood sugar diagnosis
* hedimdi   Variable label = Cardiovascular disease: diabetes or high blood sugar diagnosis newly reported (merged)
*--High blood pressure
*High blood pressure
* hedawbp   Variable label = Diagnosed high blood pressure fed forward
* hedacbp   Variable label = Whether confirms high blood pressure diagnosis
* hedimbp   Variable label = Cardiovascular disease: high blood pressure diagnosis newly reported (merged)
*--Stroke
*Stroke
* hedawst   Variable label = Diagnosed stroke fed forward
* hedacst   Variable label = Whether confirms stroke diagnosis
```

For peer review only - http://bmjopen.bmj.com/site/about/guidelines.xhtml

```
* hedimst   Variable label = Cardiovascular disease: stroke diagnosis newly reported
(merged)
*--Arthritis
*Arthritis
* hedbwar   Variable label = Chronic: diagnosed arthritis fed forwar
* hedbdar   Variable label = Whether confirms arthritis diagnosis
* hedibar   Variable label = Chronic: arthritis diagnosis newly reported
*--Osteoporosis
*Osteoporosis
* hedbwos   Variable label = Chronic: diagnosed osteoporosis fed forward
* hedbdos   Variable label = Whether confirms osteoporosis diagnosis
* hedibos   Variable label = Chronic: osteoporosis diagnosis newly reported
*--Dementia
*Dementia
* hedbwde   Variable label = Chronic: diagnosed dementia fed forward
* hedbdde   Variable label = Whether confirms dementia diagnosis
* hedibde   Variable label = Chronic: dementia diagnosis newly reported
*--Chronic lung disease
*Chronic lung disease
* hedbwlu   Variable label = Chronic: diagnosed lung disease fed forward
* hedbdlu   Variable label = Whether confirms lung disease diagnosis
* hediblu   Variable label = Chronic: lung disease diagnosis newly reported
*Asthma
* hedbwas   Variable label = Chronic: diagnosed asthma fed forward
* hedbdas   Variable label = Whether confirms asthma diagnosis
* hedibas   Variable label = Chronic: asthma diagnosis newly reported

****************************************************************************

*Data: wave_8_elsa_data_eul_v2.dta ((IFS derived databaset))

*Variables needed for health comparison from Wave 8
*Identifier
* idauniq   Variable label = Unique individual serial number
* idahhw8   Variable label = Analytical wave-specific individual
* w8xwgt    Variable label = Wave 8 cross-sectional weight

*Demography
* ageg5     Variable label = age band - 5 year bands  (8 way split)
* sex       Variable label = sex: copy of indsex/dhsex
* elsa      Variable label = Sampling status
* inst      Variable label = whether in an institution
* nonwhite  Variable label = ethic origin (white/non-white)
* marstat   Variable label = marital status - couple1 combined with dimar
* smoker    Variable label = whether current smoker
* smokerstat Variable label = smoker status (past or present)


****************************************************************************
version 15.1
clear all

cd "\Data" /* Change working directory */

*---REVIEWERS COMMENTS.

use "wave_8_elsa_data_eul_v2.dta", clear
tab indsex
/* 8445 */

keep idauniq idahhw8 perid samptyp w8xwgt indager indsex fqethnmr wpdes dimarr estwt
hedawan hedacan hediman hedawmi hedacmi hedimmi ///
        hedawhf hedachf hedimhf hedawhm hedachm hedimhm hedawar hedacar hedimar hedaw95
hedac95 hedia95 hedawdi hedacdi hedimdi ///
        hedawbp hedacbp hedimbp hedawst hedacst hedimst hedbwar hedbdar hedibar hedbwos
hedbdos hedibos ///
        hedbwde hedbdde hedibde hedbwlu hedbdlu hediblu hedbwas hedbdas hedibas


merge 1:1 idauniq idahhw8 using "wave_8_elsa_ifs_dvs_eul_v1.dta", keepusing(wgt ageg5 sex
elsa inst nonwhite smoker smokerstat)
tab _merge
drop _merge
save "ELSA Comparison.dta",replace
```

```
tab indsex
/* 8445 */

*------
*---EXCLUSION CRITERIA
*- 1. Noncore members (elsa=0 or samptyp=)
*- 2. Younger than 65 (age<65)
*- 3. Living in an institution (inst=1)

*N=8,445

*-CORE MEMBERS
tab1 elsa samptyp, m
tab samptyp elsa
*N=7,223

*-AGE
tab ageg5, m
*N=5,478


*-LIVING IN A INSTITUTION
tab inst, m
*N=58
*N=8,387

gen exclusion=1 if inst==1
replace exclusion=2 if ageg5<4
replace exclusion=3 if elsa==0
label var exclusion "Exclusion criteria"
label define exclusion 1 "Living in a institution" 2"Aged<65" 3"NoCore Members", replace
label values exclusion exclusion
tab exclusion, m


keep if exclusion==.
*N=5,065

***CLEANING AND TRANSFORMING VARIABLES

*ID
rename idauniq id
gen psu=id

*-AGE INTO 5 YEAR BANDS
tab ageg5, m

gen w8age4g=ageg5
recode w8age4g 4=1 5=2 6=3 7=4 8=4
label var w8age4g "Age into 4 groups"
label define w8age4g 1 "65-69" 2 "70-74" 3"75-79" 4"80 or more", replace
label values w8age4g w8age4g
tab w8age4g, m

tab w8age4g ageg5, m
drop ageg5

*-SEX
tab indsex

rename indsex w8sex

*1=male; 2=female
label var w8sex "Sex"

label define w8sex 1 "Male" 2 "Female", replace
label values w8sex w8sex
tab w8sex

*-ETHNICITY
tab fqethnmr nonwhite, m

rename fqethnmr w8white
recode w8white 1=1 2=0

```

```
*-MARITAL STATUS
tab dimarr, m
gen w8marstat=0
replace w8marstat=1 if  dimarr==2 | dimarr==3
replace w8marstat=. if  dimarr==-8
label var w8marstat "Current legal marital status - Married/civil partnership yes/no"
label define w8marstat 0"Other" 1"Married/civil", replace
label values w8marstat w8marstat
tab w8marstat

*-WORK STATUS
tab wpdes, m

gen w8retired=0
replace w8retired=1 if wpdes==1
label define w8retired 0"Other" 1"Retired", replace
label values w8retired w8retired
label var w8retired "Best description of current situation - Retired yes/no"
tab w8retired

*-WEIGHT
replace estwt=. if estwt<38 /***** CUT-OFF USED IN OPAL study *****/

rename estwt w8weightKg

*-SMOKE
tab smokerstat smoker
tab smokerstat, m

gen w8smokerstat=.
replace w8smokerstat=1 if smokerstat==0
replace w8smokerstat=2 if smokerstat==1 | smokerstat==2 | smokerstat==3
replace w8smokerstat=3 if smokerstat==4
lab define w8smokerstat 1"Never smoker" 2"Ex-smoker" 3"Current smoker", replace
label values w8smokerstat w8smokerstat
label variable w8smokerstat "Cigarette smoking status"

tab w8smokerstat, m

*--HEALTH-RELATED FACTORS

*Angina: ‘hedawan’, ‘hedacan’, ‘hediman’
*Heart attach: ‘hedawmi’, ‘hedacmi’, ‘hedimmi’
*Congestion heart failure: ‘hedawhf’, ‘hedachf’, ‘hedimhf’
*Heart murmur: ‘hedawhm’, ‘hedachm’, ‘hedimhm’
*Abnormal heart rhythm: ‘hedawar’, ‘hedacar’, ‘hedimar’
*Other: ‘hedaw95’, ‘hedac95’, 'hedia95'

*-ANGINA
tab1 hedawan hedacan hediman
gen w8angina=0
replace w8angina=1 if (hedawan==2 & hedacan==1) | hediman==1
replace w8angina=. if hediman<0 & w8angina!=1

*-HEART ATTACK
tab1 hedawmi hedacmi hedimmi
gen w8heartattack=0 if hedawmi==-1
replace w8heartattack=1 if (hedawmi==3 & hedacmi==1) | hedimmi==1
replace w8heartattack=. if hedimmi<0 & w8heartattack!=1

*-CONGESTION HEART FAILURE
tab1 hedawhf hedachf hedimhf
gen w8heartfailure=0
replace w8heartfailure=1 if (hedawhf==4 & hedachf==1) | hedimhf==1
replace w8heartfailure=. if hedimhf<0 & w8heartfailure!=1

*-HEART MURMUR
tab1 hedawhm hedachm hedimhm
gen w8heartmurmur=0
replace w8heartmurmur=1 if (hedawhm==5 & hedachm==1) | hedimhm==1
replace w8heartmurmur=. if hedimhm<0 & w8heartmurmur!=1

*-ABNORMAL HEART RHYTHM
tab1 hedawar hedacar hedimar
gen w8heartrhythm=0
```

```
replace w8heartrhythm=1 if (hedawar==6 & hedacar==1) | hedimar==1
replace w8heartrhythm=. if hedimar<0 & w8heartrhythm!=1

*-OTHER
tab1 hedaw95 hedac95 hedia95
gen w8otherheartproblem=0
replace w8otherheartproblem=1 if (hedaw95==95 & hedac95==1) | hedia95==1
replace w8otherheartproblem=. if hedia95<0 & w8otherheartproblem!=1

*-HEART PROBLEMS
gen w8hearttroubles=0
replace w8hearttroubles=1 if w8angina==1 | w8heartattack==1 | w8heartfailure==1 |
w8heartmurmur==1 | w8heartrhythm==1 | w8otherheartproblem==1
replace w8hearttroubles=. if w8angina==. & w8heartattack==. & w8heartfailure==. &
w8heartmurmur==. & w8heartrhythm==. & w8otherheartproblem==.

*-DIABETES
tab1 hedawdi hedacdi hedimdi
gen w8diabetes=0
replace w8diabetes=1 if (hedawdi==7 & hedacdi==1) | hedimdi==1
replace w8diabetes=. if hedimdi<0 & w8diabetes!=1

*-HIGH BLOOD PRESSURE
tab1 hedawbp hedacbp hedimbp
gen w8hbp=0
replace w8hbp=1 if (hedawbp==1 & hedacbp==1) | hedimbp==1
replace w8hbp=. if hedimbp<0 & w8hbp!=1

*-STROKE
tab1 hedawst hedacst hedimst
gen w8stroke=0
replace w8stroke=1 if (hedawst==8 & hedacst==1) | hedimst==1
replace w8stroke=. if hedimst<0 & w8stroke!=1
tab w8stroke

*-ARTHRITIS
tab1 hedbwar hedbdar hedibar
gen w8arthritis=0
replace w8arthritis=1 if (hedbwar==3 & hedbdar==1) | hedibar==1
replace w8arthritis=. if hedibar<0 & w8arthritis!=1

*-DEMENTIA
tab1 hedbwde hedbdde hedibde
gen w8dementia=0
replace w8dementia=1 if (hedbwde==9 & hedbdde==1) | hedibde==1
replace w8dementia=. if hedibde<0 & w8dementia!=1

*-OSTEOPOROSIS
tab1 hedbwos hedbdos hedibos
gen w8osp=0
replace w8osp=1 if (hedbwos==4 & hedbdos==1) | hedibos==1
replace w8osp=. if hedibos<0 & w8osp!=1

*-CHRONIC LUNG DISEASE
tab1 hedbwlu hedbdlu hediblu
gen w8lungdisease=0
replace w8lungdisease=1 if (hedbwlu==1 & hedbdlu==1) | hediblu==1
replace w8lungdisease=. if hediblu<0 & w8lungdisease!=1

*-ASTHMA
tab1 hedbwas hedbdas hedibas

gen w8asthma=0
replace w8asthma=1 if (hedbwas==1 & hedbdas==1) | hedibas==1
replace w8asthma=. if hedibas<0 & w8asthma!=1

*-CHRONIC LUNG DISEASE + ASTHMA
gen w8cld=0
replace w8cld=1 if w8lungdisease==1 | w8asthma==1
replace w8cld=. if w8lungdisease==. & w8asthma==.

/*****   ANALYSIS  WITH WEIGHTED DATA ******/

svyset, clear
svyset [pweight=w8xwgt], psu(psu)
```

```
/***** FEMALE ****/

unab xvars: w8white w8marstat w8retired w8smokerstat w8hearttroubles w8diabetes w8hbp
w8stroke w8arthritis w8dementia w8osp w8cld
foreach x of local xvars {
svy:tab `x' w8age4g if w8sex==2, col per ci
}

*---Weight (Kg)
forvalues i = 1/4 {
svy:mean w8weightKg if w8sex==2 & w8age4g==`i'
}

*---Unweighted N
tab w8age4g if w8sex==2


/***** MALE ****/
unab xvars: w8white w8marstat w8retired w8smokerstat w8hearttroubles w8diabetes w8hbp
w8stroke w8arthritis w8dementia w8osp w8cld
foreach x of local xvars {
svy:tab `x' w8age4g if w8sex==1, col per ci
}

*---Weight (Kg)
forvalues i = 1/4 {
svy:mean w8weightKg if w8sex==1 & w8age4g==`i'
}

*---Unweighted N
tab w8age4g if w8sex==1
```

review only
